# Accurate computational design of three-dimensional protein crystals

**DOI:** 10.1038/s41563-023-01683-1

**Published:** 2023-10-16

**Authors:** Zhe Li, Shunzhi Wang, Una Nattermann, Asim K. Bera, Andrew J. Borst, Muammer Y. Yaman, Matthew J. Bick, Erin C. Yang, William Sheffler, Byeongdu Lee, Soenke Seifert, Greg L. Hura, Hannah Nguyen, Alex Kang, Radhika Dalal, Joshua M. Lubner, Yang Hsia, Hugh Haddox, Alexis Courbet, Quinton Dowling, Marcos Miranda, Andrew Favor, Ali Etemadi, Natasha I. Edman, Wei Yang, Connor Weidle, Banumathi Sankaran, Babak Negahdari, Michael B. Ross, David S. Ginger, David Baker

**Affiliations:** 1 Department of Biochemistry, University of Washington, Seattle, WA, USA.; 2 Institute for Protein Design, University of Washington, Seattle, WA, USA.; 3 Graduate Program in Biological Physics, Structure & Design, University of Washington, Seattle, WA, USA.; 4 Department of Chemistry, University of Washington, Seattle, WA, USA.; 5 X-Ray Science Division, Advanced Photon Source, Argonne National Laboratory, Argonne, IL, USA.; 6 Molecular Biophysics and Integrated Bioimaging Division, Lawrence Berkeley National Laboratory, Berkeley, CA, USA.; 7 HHMI, University of Washington, Seattle, WA, USA.; 8 Molecular Engineering and Sciences Institute, University of Washington, Seattle, WA, USA.; 9 Medical Biotechnology Department, School of Advanced Technologies in Medicine, Tehran University of Medical Sciences (TUMS), Tehran, Iran.; 10 Molecular and Cellular Biology Graduate Program, University of Washington, Seattle, WA, USA.; 11 Medical Scientist Training Program, University of Washington, Seattle, WA, USA.; 12 Department of Chemistry, University of Massachusetts Lowell, Lowell, MA, USA.; 13 These authors contributed equally: Zhe Li, Shunzhi Wang, Una Nattermann.

## Abstract

Protein crystallization plays a central role in structural biology. Despite this, the process of crystallization remains poorly understood and highly empirical, with crystal contacts, lattice packing arrangements and space group preferences being largely unpredictable. Programming protein crystallization through precisely engineered side-chain–side-chain interactions across protein–protein interfaces is an outstanding challenge. Here we develop a general computational approach for designing three-dimensional protein crystals with prespecified lattice architectures at atomic accuracy that hierarchically constrains the overall number of degrees of freedom of the system. We design three pairs of oligomers that can be individually purified, and upon mixing, spontaneously self-assemble into >100 µm three-dimensional crystals. The structures of these crystals are nearly identical to the computational design models, closely corresponding in both overall architecture and the specific protein–protein interactions. The dimensions of the crystal unit cell can be systematically redesigned while retaining the space group symmetry and overall architecture, and the crystals are extremely porous and highly stable. Our approach enables the computational design of protein crystals with high accuracy, and the designed protein crystals, which have both structural and assembly information encoded in their primary sequences, provide a powerful platform for biological materials engineering.

The structures of hundreds of thousands of proteins have been determined by X-ray crystallography^1^. The protein crystals required for this process are typically generated by screening a wide range of crystallization solution conditions. The crystals are held together by numerous but weak non-covalent interactions (crystal contacts)^2^. Despite the huge amount of effort devoted to protein crystallography, it remains a poorly understood and highly empirical process^3,4^. There has been exciting progress in introducing metal-binding sites^5,6^, electrostatics^7,8^, DNA^9,10^ and aromatic–aromatic interactions^11^ to drive three-dimensional (3D) crystal formation, but it has not been possible to accurately design and program the atomic details of the assembled crystals. A 3D crystal has been computationally designed from short peptides^12^, but the design strategy is not generally applicable to proteins of higher structural complexity. Despite considerable advances in computational protein design^13^, the general problem of designing specific side-chain–side-chain interactions across protein interfaces that direct proteins to self-assemble into prespecified 3D crystal lattices with atomic-level accuracy remains unsolved^14–16^. The problem is challenging because multiple (typically three or more) non-covalent protein interaction surfaces with high specificity need to be designed into the monomeric subunits, and each designed interface must have high geometric precision to drive packing into the target crystal lattice. Small deviations in the interface geometry can add up to large deviations over multiple unit cells and, hence, disrupt assembly.

In this work, we develop a generally applicable hierarchical approach to 3D protein crystal design. We use the approach to design protein crystals that are programmable at atomic accuracy, highly porous and extremely robust. They constitute a new class of genetically encodable biomaterials for applications^17^ such as biocatalysis^18^, sensing^19^, chemical separation^20^ and drug delivery^21^.

## Hierarchical computational design strategy

**Fig. 1 |:**
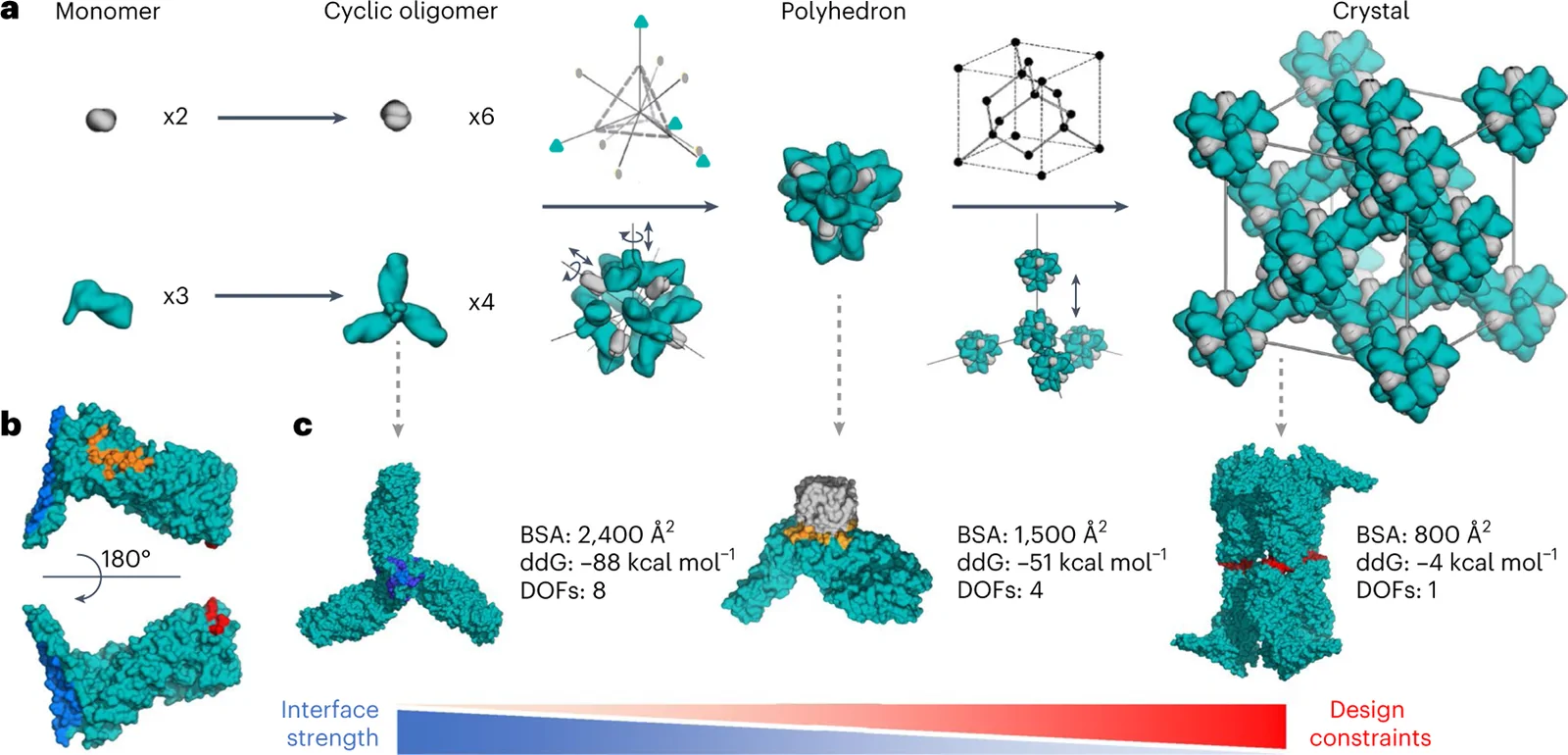
Hierarchical crystal design strategy. **a**, Schematic illustration of the three-step design hierarchy for a diamond lattice (*F*4_1_32 space group) formed from a tetrahedral polyhedron built from a C2 dimer (grey) and a C3 trimer (cyan). Monomers (first column) are docked into cyclic dimers and trimers (second column), which are docked into a two-component cage (third column), which is then arrayed in a 3D lattice (fourth column). **b**, Interfaces driving crystal assembly. For the designed crystal example in **a**, the three designed interfaces on the trimer component are shown that drive the assembly of the cyclic oligomer (blue), the tetrahedron (orange) and the crystal lattice (red). **c**, Interfaces mapped to the monomer in **b** are shown between interacting partners for the trimer (left), tetrahedral cage (middle) and crystal (right). To maximize assembly cooperativity, the interface size (BSA, buried surface area) and affinity (Rosetta calculated ddG^30^) decrease through the design hierarchy; the number of system degrees of freedom available for sampling at each step (Methods) decreases in parallel, making the design challenge more difficult.

We set out to develop a divide and conquer hierarchical approach to sequentially design the multiple interfaces required for crystal formation. Crystal space groups in three dimensions are generated from the combination of crystallographic point groups and Bravais lattices^22^. We reasoned that it should be possible to first design interfaces directing the assembly of protein monomers into assemblies with point group symmetry and then, following experimental validation, introduce a third interface constraining the translation of these assemblies to generate the desired Bravais lattice. Here, we focused on space groups containing high symmetry point groups (tetrahedral (T) and octahedral (O)) related by dihedral centres: *P*23, *P*432, *P*4_2_32, *I*432, *F*23, *F*432 and *F*4_1_32 (refs. 23,24; Supplementary Figs. 1 and 2). In the example shown in Fig. 1a, a diamond lattice of the *F*4_1_32 space group can be constructed by arranging tetrahedral cages formed from two distinct protein monomeric subunits (cyan and grey).

**Extended Data Fig. 1 |:**
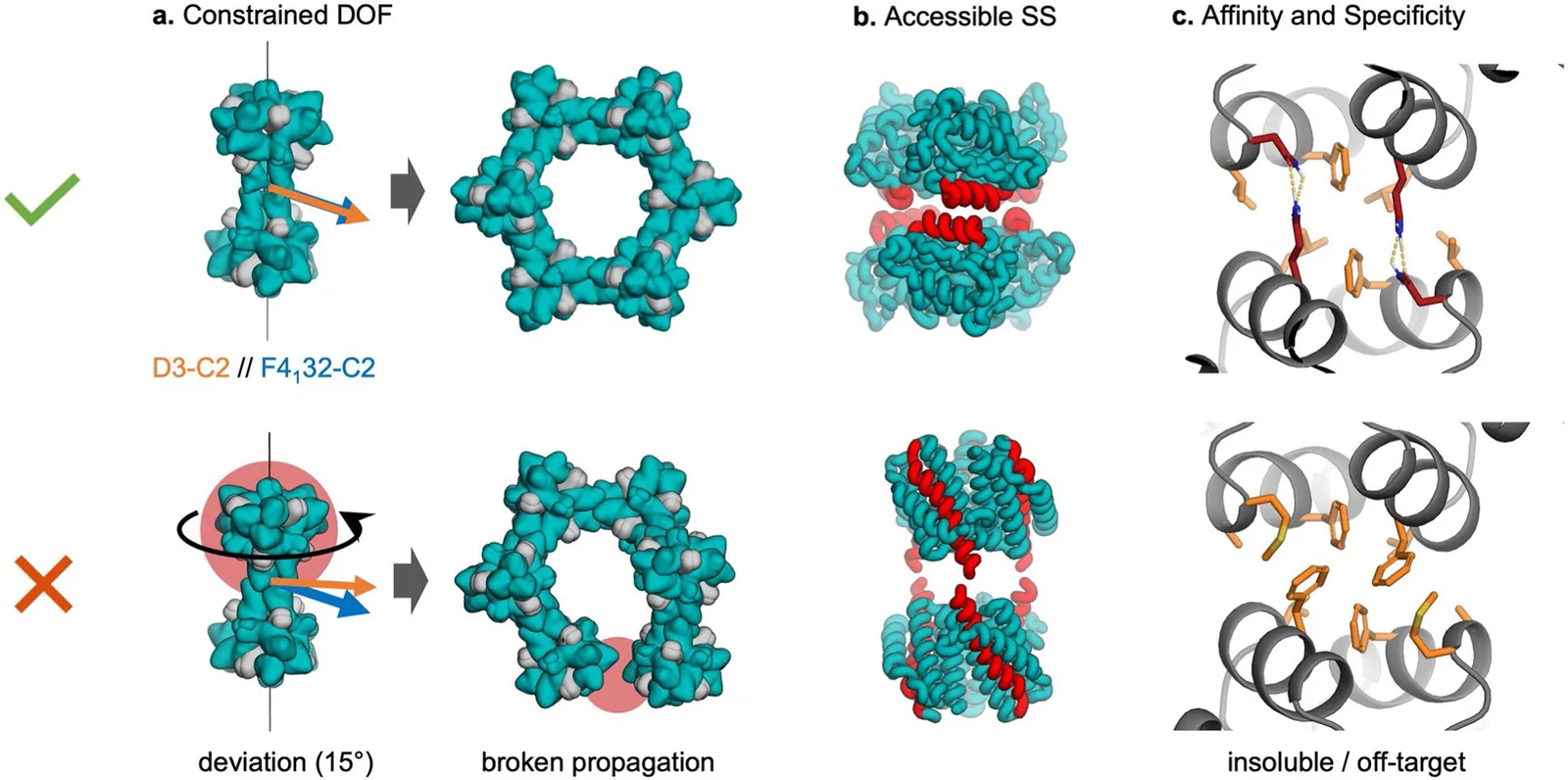
Design rules of 3D protein crystals. **a**, Constrained degree of freedom (DOF): The angle of rotation at the designed dihedral crystal interface (Fig. 1a, right panel) must be precisely specified by the design process, where the C2 axis of the dihedral needs to coincide with the C2 axis of the space group. In this example, the disruptive effect (highlighted in red) of a 15-degree error in alignment on crystal assembly is illustrated; similar crystal lattice breakdowns occur with all deviations from the target alignment angle. **b**, Accessible secondary structure (SS): Dihedral interfaces with helices perpendicular to the symmetry axis (docked from T33–15 cage) are more designable than those with helices parallel to the symmetry axis (docked from T33–21 cage^26^). Interacting secondary structures are highlighted in red. **c**, Affinity and Specificity: Working interfaces have sufficient hydrophobic packing with specific polar interactions at the boundary. Highly hydrophobic interfaces destruct the designed self-assembly, including insoluble components and off-target assemblies.

The successful implementation of this design strategy requires three orthogonal interfaces on the same protein component (Fig. 1b) with decreasing interface strength at each assembly step to maximize assembly cooperativity. The geometric precision must be high to satisfy the overall crystal lattice symmetry constraints (Fig. 1c). The hierarchical design of the first two interfaces has been achieved previously^25–29^, but the design of the third interface is an unsolved challenge as only a single translational degree of freedom (DOF) is available for sampling, making it difficult to generate favourable shape-complementary backbone arrangements for sequence design. The rightmost diagram of Fig. 1c shows the interface formed between two tetrahedra interacting through two trimers adopting a dihedral D3 arrangement. The targeted *F*4_1_32 space group requires that the C2 axes of the D3 dihedral and the crystal are coincident (Extended Data Fig. 1a). To address this issue, during the point group symmetry construction stage, we implemented geometric filters to ensure that the secondary structure interaction between the trimer building blocks along the direction of cage docking enabled the design of specific side-chain–side-chain interactions across the interface (Extended Data Fig. 1b); the Bravais-lattice-generating interface must specifically encode the desired crystal lattice and be orthogonal to the cage and oligomer interfaces so as not to interfere with cage assembly or promote off-target assemblies. In addition to proper backbone docking geometry, the crystal contact interface must be in a narrow window of interaction strengths (Extended Data Fig. 1c, Supplementary Fig. 3 and Supplementary Table 4). If it is too strong, there will be little dissociation between interacting cages and, hence, limited self-correction of assembly errors, resulting in kinetic traps. If it is too weak, there will be insufficient force to drive the assembly of the target crystal structure.

**Extended Data Fig. 2 |:**
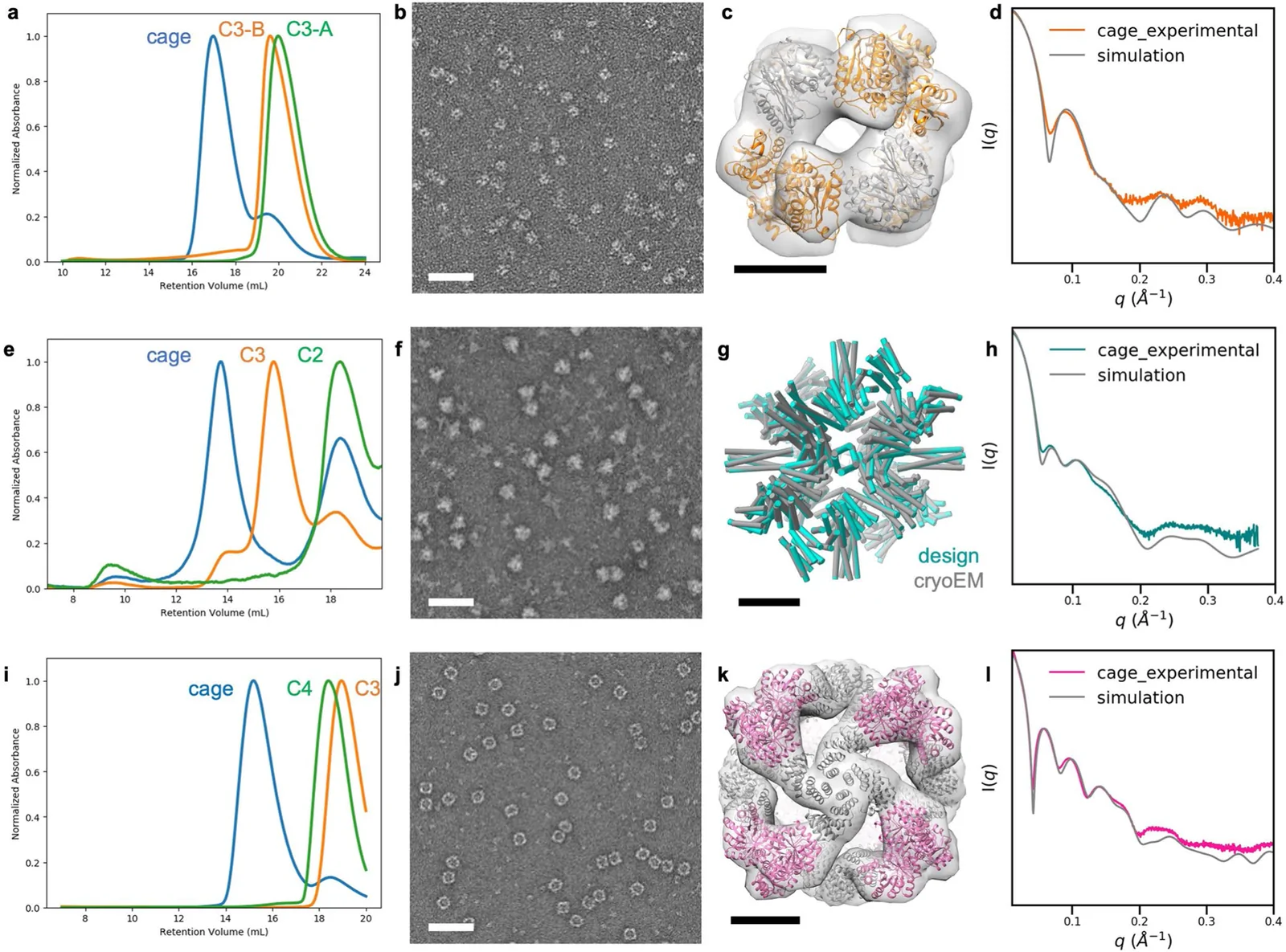
Characterizations of the constituent cages of designed crystals. **a**-**d**, T33–15-D3–4, **e**–**h**, T32–15, i-l, O43–2. **a**,**e**,**i**, SEC chromatograms of two oligomeric components (green and orange) and cages assembled via in-vitro mixing of components (blue). **b**,**f**,**j**, nsEM images (scale bars, 50 nm). **c**,**g**,**k**, overlay of the design model with 3D reconstructed nsEM density map/cryoEM model (scale bars, 5 nm). **d**,**h**,**l**, SAXS profile and simulation results of cages.

**Extended Data Fig. 3 |:**
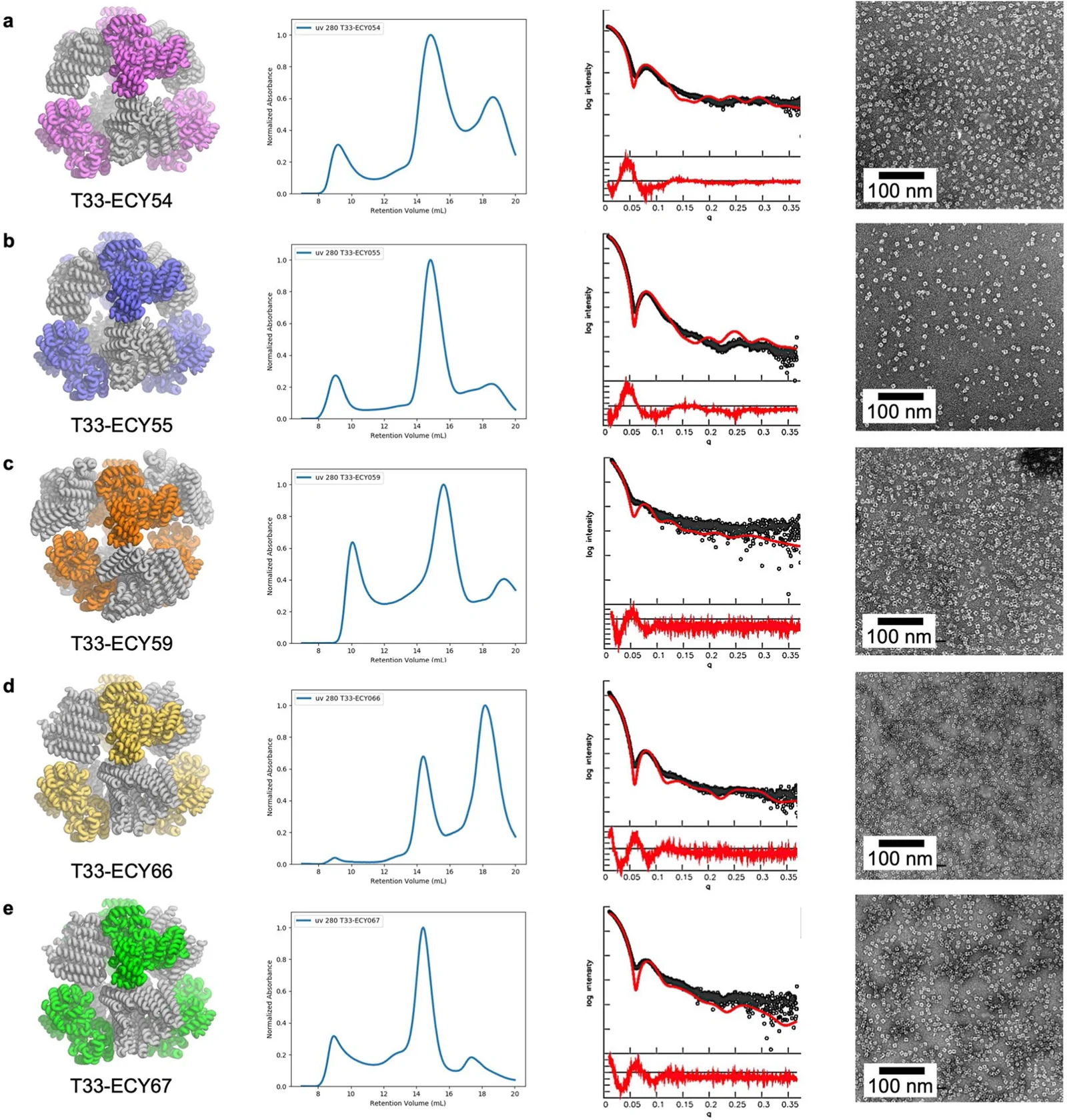
Characterizations of new tetrahedral cages for crystal design. **a**–**e**, from left to right, computational model, SEC chromatogram, SAXS profile, and nsEM images.

**Extended Data Fig. 4 |:**
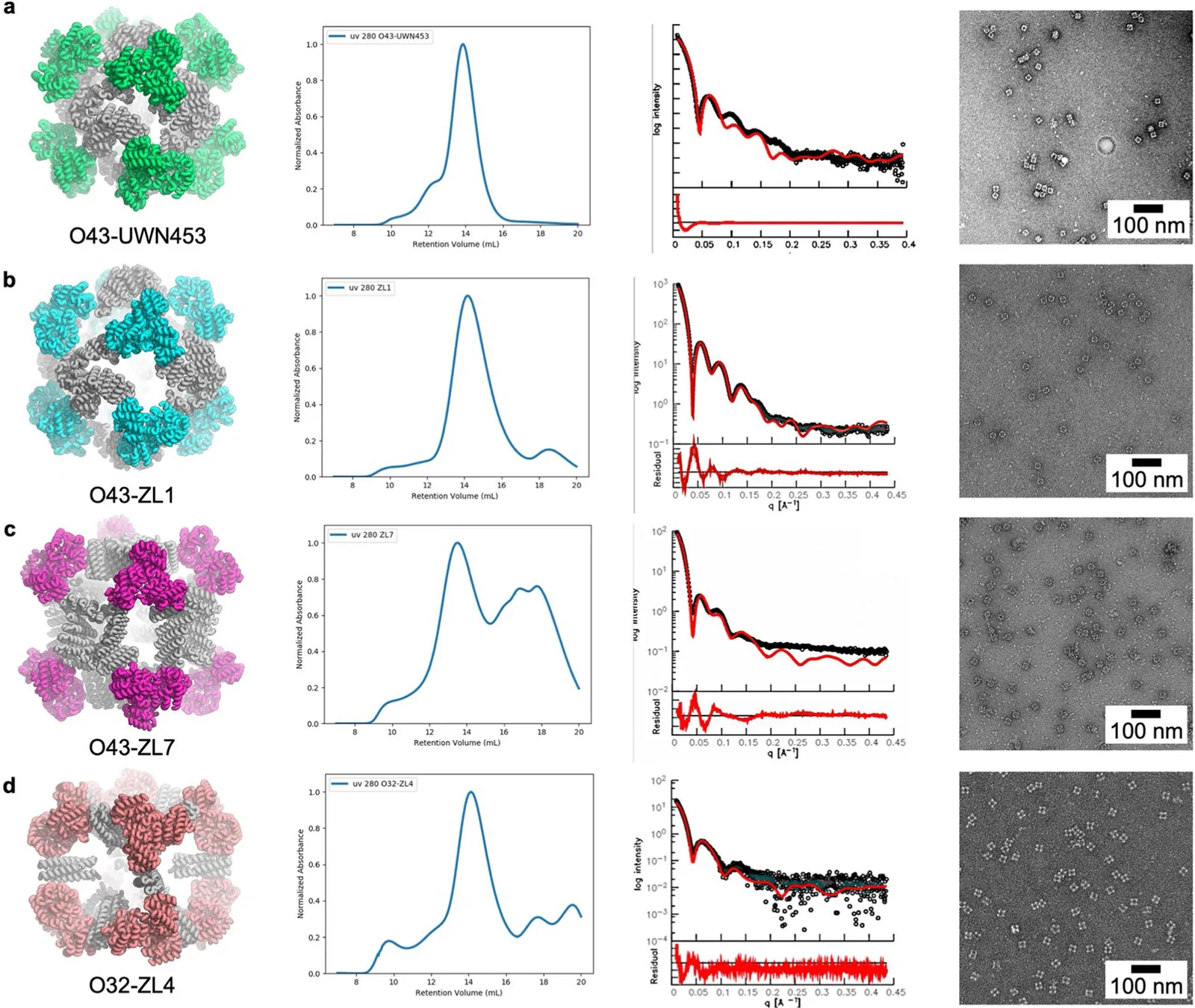
Characterizations of new octahedral cages for crystal design. **a**–**d**, from left to right, computational model, SEC chromatogram, SAXS profile, and nsEM images.

**Extended Data Fig. 5 |:**
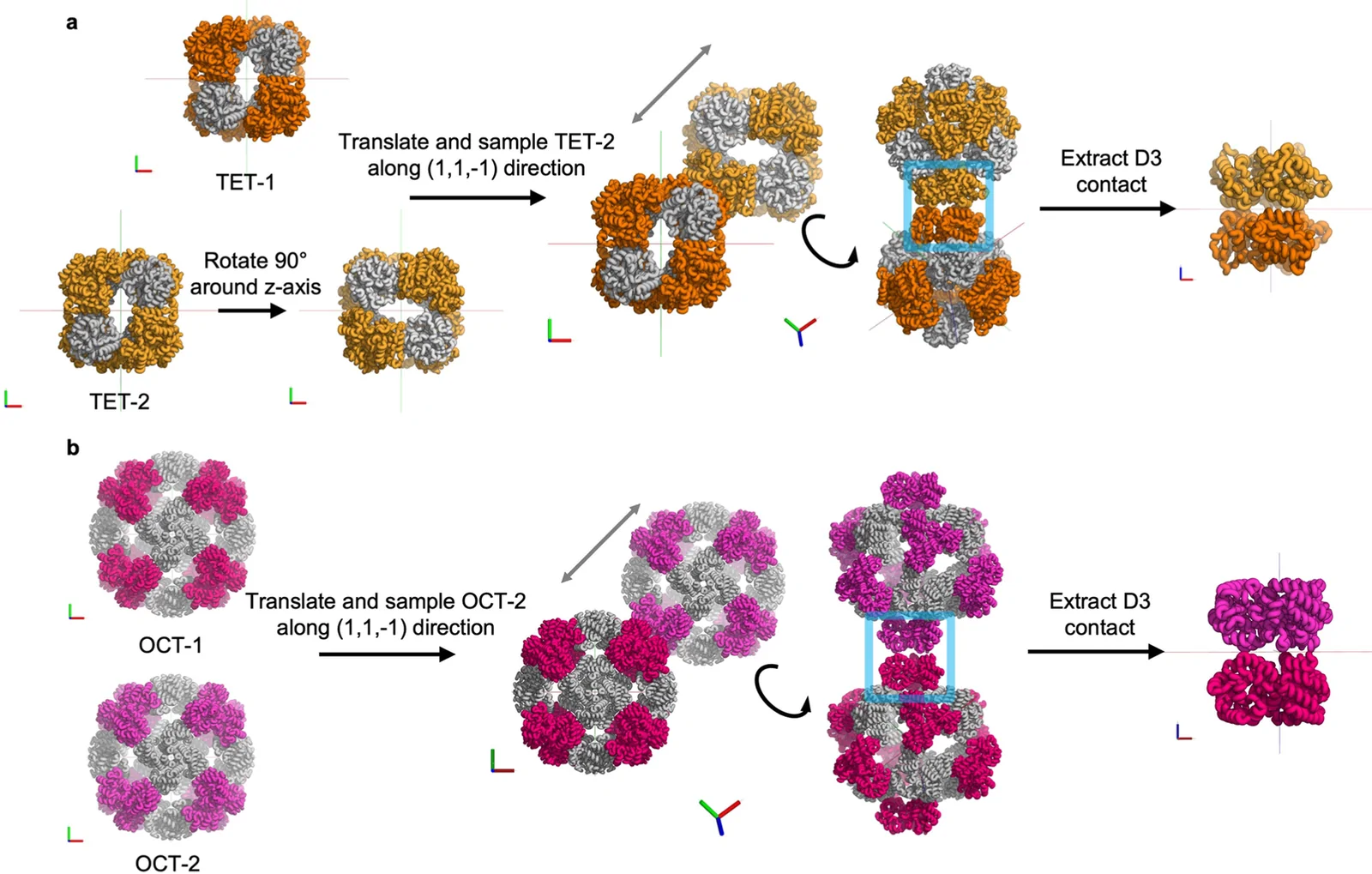
Symmetric dockings of tetrahedral and octahedral cages into crystal lattices. **a**, Two tetrahedral cages are docked along their C3 axis for crystal contacts of D3 dihedrals, which allow them to crystallize in the F4_1_32 space group. **b**, Two octahedral cages are docked along their C3 axis for crystal contacts of D3 dihedrals, which allow them to crystallize in the I432 space group. See methods for detailed docking protocol.

To implement this crystal design strategy, we designed new protein polyhedra favourable for assembly into crystals (Extended Data Figs. 2e–l, 3 and 4 and Supplementary Fig. 4) by using RPXDock^30^ to dock a wide range of designed oligomers^31–35^ with C2, C3 or C4 symmetry into T33, T32, O43, O42 and O32 assemblies. The two indices indicate the symmetries of the constituent oligomers; O43 indicates an octahedron generated from a cyclic tetramer and a cyclic trimer. We then designed sequences using Rosetta^36^. Following experimental validation, the polyhedra were placed into the corresponding Wyckoff sites in the target crystal lattice, with the translational spacing sampled finely around 3 Å of close contact (Methods and Extended Data Fig. 5). The crystal-forming interfaces were designed using Rosetta to be specific while having lower affinity than other interfaces within the polyhedra to increase assembly cooperativity.

## Design and characterization of *F*4_1_32–1 crystals

**Extended Data Fig. 6 |:**
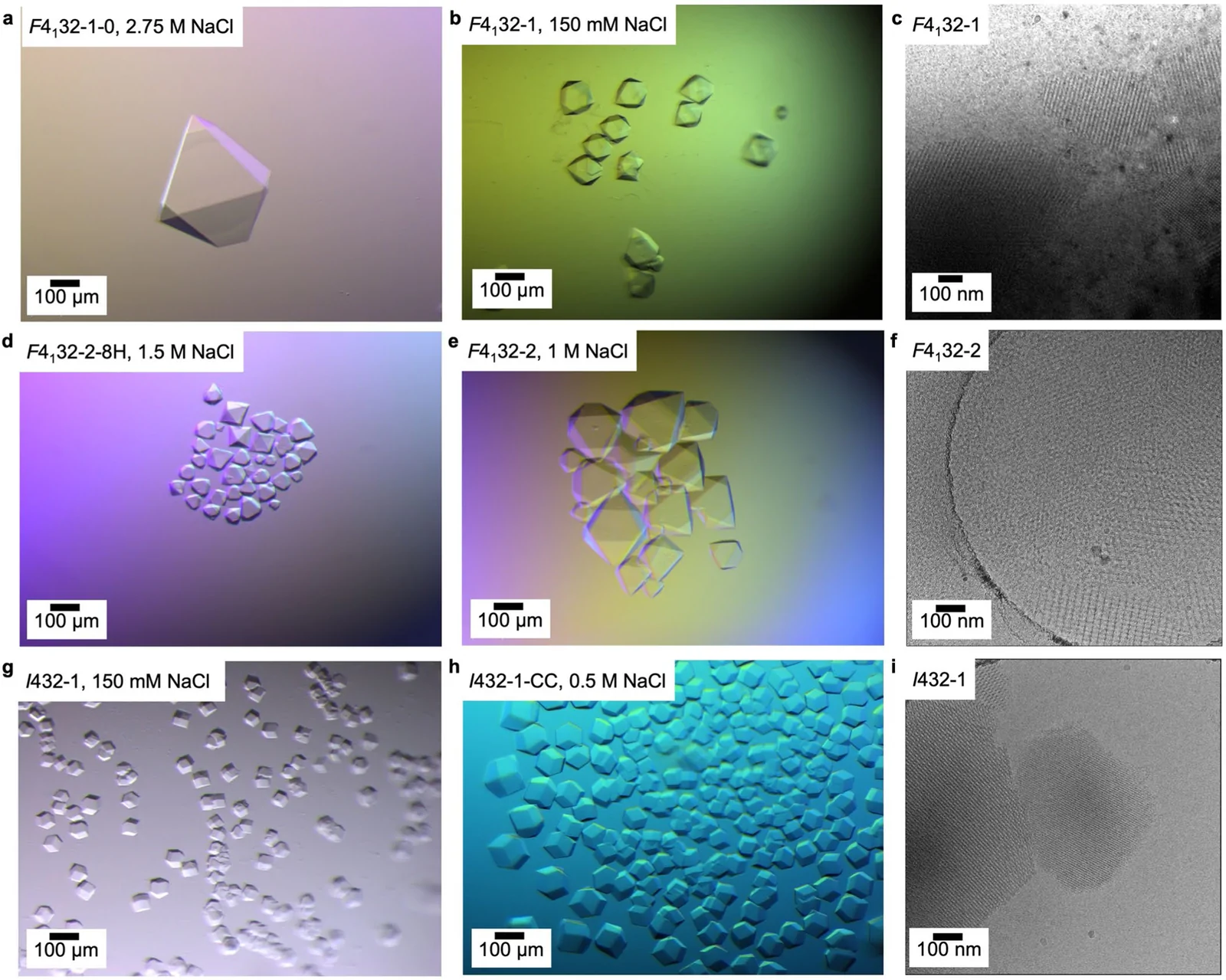
Optical microscopy and cryoEM characterization of designed protein crystals. **a**, Optical micrograph of F4_1_32–1-0 crystals. **b**, Optical micrograph of F4_1_32–1 crystals. **c**, CryoEM image of F4_1_32–1 crystals. **d**, Optical micrograph of F4_1_32–2-6H crystals. **e**, Optical micrograph of F4_1_32–2 crystals. **f**, CryoEM image of F4_1_32–2 crystals. **g**, Optical micrograph of I432–1 crystals. **h**, Optical micrograph of I432–1-CC crystals. **i**, CryoEM image of I432–1 crystals.

**Fig. 2 |:**
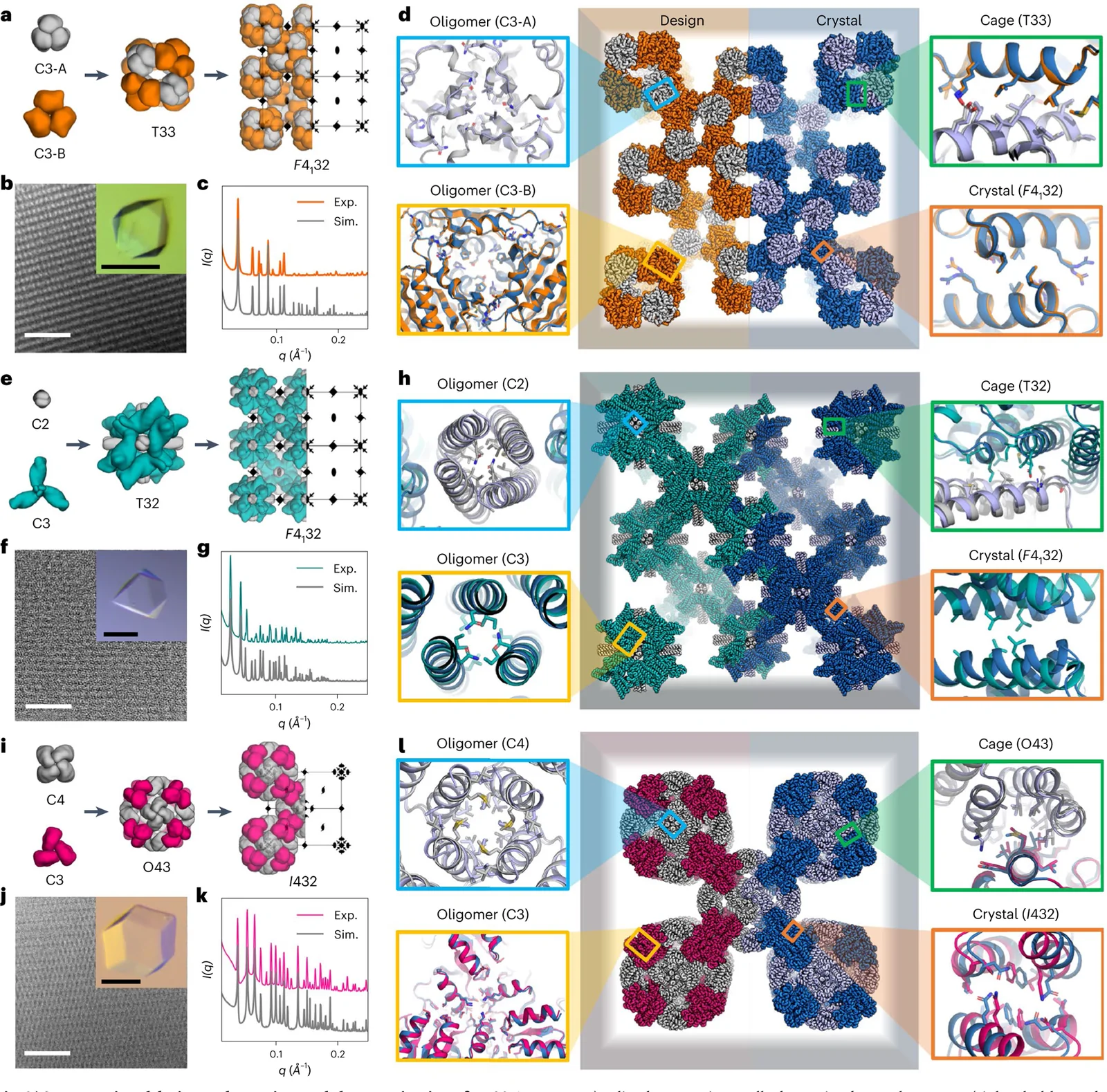
Computational design and experimental characterization of *F*4_1_32–1, *F*4_1_32–2 and *I*432–1 crystals. **a**, Construction of *F*4_1_32–1 crystals from cyclic oligomers. In the second step, symmetry elements of the cage are superimposed with corresponding symmetry elements of the unit cell. **b**, CryoEM images of *F*4_1_32–1 crystals (scale bar, 100 nm) and optical micrographs of single crystals (inset, scale bar, 100 µm). **c**, SAXS profiles of *F*4_1_32–1 microcrystals (orange) compared to simulated profiles computed from the design model (grey). **d**, Computational design model of *F*4_1_32–1 crystals (left, orange and grey) spliced to experimentally determined crystal structure (right, sky blue and light blue). The flanking panels are enlargements of the four designed interfaces, with the design model superimposed on the crystal structure. **e**, Construction of *F*4_1_32–2 crystals from cyclic oligomers. In the second step, symmetry elements of the cage are superimposed with corresponding symmetry elements of the unit cell. **f**, CryoEM images of *F*4_1_32–2 crystals (scale bar, 100 nm) and optical micrographs of single crystals (inset, scale bar, 100 µm). **g**, SAXS profiles of *F*4_1_32–2 microcrystals (teal) compared to simulated profiles computed from the design model (grey). **h**, Computational design model of *F*4_1_32–2 crystals (left, teal and grey) spliced to experimentally determined crystal structure (right, sky blue and light blue). The flanking panels are enlargements of the four designed interfaces, with the design model superimposed on the crystal structure. **i**, Construction of *I*432–1 crystals from cyclic oligomers. In the second step, symmetry elements of the cage are superimposed with corresponding symmetry elements of the unit cell. **j**, CryoEM images of *I*432–1 crystals (scale bar, 100 nm) and optical micrographs of single crystals (inset, scale bar, 100 µm). **k**, SAXS profiles of *I*432–1 microcrystals (pink) compared to simulated profiles computed from the design model (grey). **l**, Computational design model of *I*432–1-CC crystals (left, pink and grey) spliced to experimentally determined crystal structure (right, sky blue and light blue). The flanking panels are enlargements of the four designed interfaces, with the design model superimposed on the crystal structure. There is a close agreement in all three cases. **d**,**h**,**l**, Left top and bottom: the two cyclic oligomer interfaces. Top right: interface between cyclic oligomers that generates the polyhedral cage. Bottom right: interface between polyhedra that generates the crystal. All-atom RMSDs are calculated for each symmetry unit and summarized in Supplementary Table 5. Exp., experimental; Sim., simulated.

To independently evaluate the last step in our hierarchical approach, we first sought to assemble previously designed tetrahedral cages^25–28^ into *F*4_1_32 lattices through a newly designed D3 symmetry cage–cage interface. In a first round of design calculations, all high-scoring designs were found to employ the same building block, T33–15 (ref. 26), a 24-subunit tetrahedral cage composed of two distinct cyclic trimeric building blocks C3-A and C3-B, with C3-B forming the extended crystal interface (Fig. 2a). The T33–15 cage can be assembled in vitro from separately purified components^26^, which enabled facile screening of crystal designs by mixing C3-B containing the crystal interface with the unmodified C3-A in vitro at a 1:1 molar ratio in hanging drops (Methods). For the design T33–15-D3–4, we observed octahedral-shaped crystals up to 200 µm in diameter (F4_1_32–1-0) over a week (Extended Data Fig. 6a), which is the expected Wulff polyhedral crystal habit for diamond crystals (Supplementary Fig. 5)^37^. With a Thr to Arg substitution at the periphery of the crystal interface, crystals formed within 12 h of mixing without additional precipitant; we refer to this crystal as *F*4_1_32–1 (Fig. 2b inset and Extended Data Fig. 6b). To examine the structure of the constituent cage without the complications of rapid crystal assembly, we characterized T33–15-D3–4 before crystallization by size exclusion chromatography (SEC; Extended Data Fig. 2a), negative-stain electron microscopy (nsEM; Extended Data Fig. 2b,c) and small-angle X-ray scattering (SAXS; Extended Data Fig. 2d). The results were consistent with the designed cage structure. To study the crystal packing and molecular details of the *F*4_1_32–1 crystal interface, we characterized the *F*4_1_32–1 crystals by cryogenic electron microscopy (cryoEM; Fig. 2b and Extended Data Fig. 6c), SAXS (Fig. 2c) and X-ray crystallography (Fig. 2d). SAXS data collected at room temperature indicated single-phase *F*4_1_32 symmetry with a unit cell diameter of 295 Å, in close agreement with the designed lattice model (297 Å). The experimental scattering intensity profile closely matched that computed from the designed crystal lattice (Fig. 2c). A 2.80 Å resolution X-ray structure of the crystal showed that both the overall designed *F*4_1_32 lattice and the individual subunit–subunit interfaces were closely recapitulated (Fig. 2d). The all-atom root-mean-square deviation (RMSD) between the X-ray structure and computational design model over the full cage was 0.88 Å (Supplementary Fig. 6a), over the six monomers that form the dihedral tetrahedron–tetrahedron interface, 1.19 Å (Supplementary Fig. 6b), and over the two full contacting cages, 1.39 Å (Supplementary Fig. 6c). The crystal structure of the original T33–15-D3–4 design (before the Thr55Arg substitution, Supplementary Fig. 7) has a slightly higher RMSD (1.81 Å) than the design model, likely due to the formation of an unintended hydrogen bond by the Thr (Supplementary Fig. 8).

## Design and characterization of *F*4_1_32–2 crystals

Although the *F*4_1_32–1 crystal design demonstrated the capability of our computational protocol in programming assembly to prespecified crystal lattices with angstrom-level resolution, it also highlighted some remaining challenges. First, there is the fine balance between interface affinity and specificity required to drive the assembly process while avoiding off-target interactions (Extended Data Fig. 1c); many designs were insoluble or formed off-target assemblies likely due to overly extensive designed crystal interfaces (Supplementary Fig. 9). Second, the previously characterized polyhedral protein assemblies only allowed limited exploration of the crystal design space because the constituent oligomeric building blocks in most cases did not present designable secondary structure elements capable of forming the new crystal interface (Extended Data Fig. 1b). Hence, we designed a new set of polyhedral cages specifically for use as building blocks for crystals (Extended Data Figs. 2e–l, 3 and 4 and Supplementary Fig. 4).

**Extended Data Fig. 7 |:**
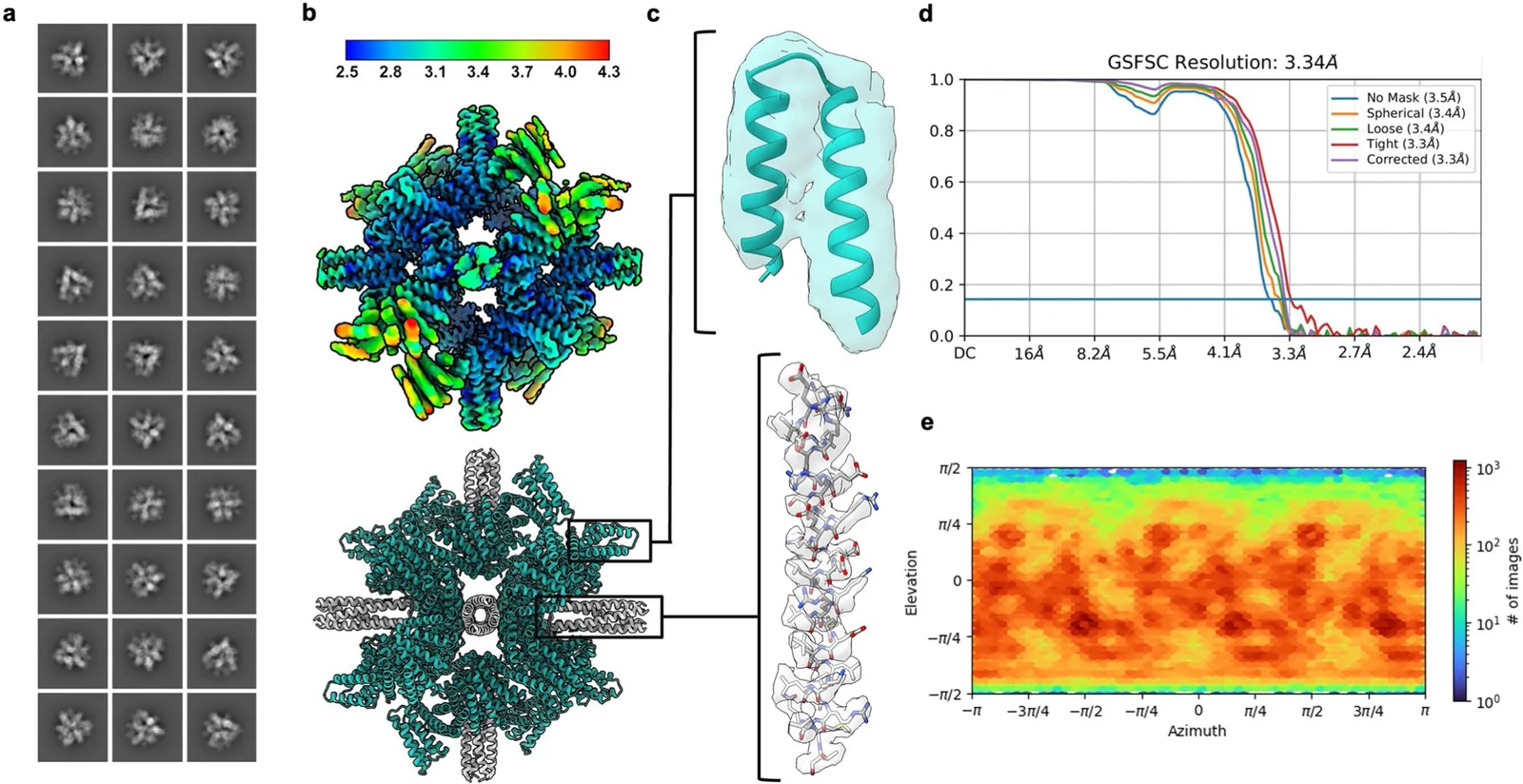
CryoEM data of the T32–15 cage. **a**, Representative 2D class averages of the T32–15 cage. **b**, CryoEM local resolution map of the T32–15 cage (top) and built atomic model (bottom). Local resolution estimates range from ~2.5 Å at the core to ~4 Å along the crystal-contact forming helices. **c**, Map-to-model comparison within a low-resolution region (top) and a high-resolution region (bottom). **d**, Global FSC. **e**, Orientational distribution plot demonstrating full angular sampling.

We set out to design a new diamond lattice using the new polyhedral building blocks (Fig. 2e). We first designed a T32 cage from a pH-responsive trimer^34^ rigidly fused^35^ to designed helical repeat proteins (DHRs)^31^ (HFuse_pH192_0046, J. M. Lubner, Y. Chang, R. Redler, G. Bhabha, D. Ekiert and D. Baker, manuscripts in preparation) and a helical bundle dimer (2L4HC2_23)^32^ (Supplementary Fig. 10a). We designed this cage, T32–15, such that when docked into a diamond lattice, it forms dihedral D3 crystal contact interfaces at the facets defined by the centres of three neighbouring trimers along the other C3 symmetry axis of the tetrahedron (Fig. 1c, right panel), unlike the vertices defined by a single trimer as in the *F*4_1_32–1 design (Extended Data Fig. 1b, top panel). We confirmed cage formation by immobilized-metal affinity chromatography (IMAC) pull-down followed by sodium dodecyl sulfate–polyacrylamide gel electrophoresis (SDS–PAGE; Supplementary Fig. 10b), SEC (Extended Data Fig. 2e), nsEM (Extended Data Fig. 2f and Supplementary Fig. 10c) and SAXS (Extended Data Fig. 2h) and determined the structure by cryoEM with a global 3.34 Å resolution (Extended Data Figs. 2g and 7). The cryoEM structure closely matched the design model over both designed components, with some deviation in the helix orientation of the C3 component at the periphery of the cage (Extended Data Fig. 2g). Six design variants of the crystal lattice interface were generated, and after mixing the independently purified components, design T32–15-D3–6 formed octahedral-shaped crystals (*F*4_1_32–2-8H, Extended Data Fig. 6d). We found that shortening the helices of the C2 component increased its solubility and the yield of cage assembly following mixing (Supplementary Fig. 11). This trimmed cage, T32–15-D3–6-6H, formed ~100 µm sized octahedral-shaped crystals (*F*4_1_32–2) in 3 to 4 days (Fig. 2f inset and Extended Data Fig. 6e). Crystals were characterized by cryoEM (Fig. 2f and Extended Data Fig. 6f), SAXS (Fig. 2g) and X-ray crystallography (Fig. 2h). Indexing of the experimental SAXS profile of *F*4_1_32–2 microcrystals indicated the desired *F*4_1_32 space group with a unit cell edge length of 412 Å, in close agreement with the designed lattice model (417 Å, Fig. 2g), with no evidence of mixed phases being present. We solved the crystal structure of the designed lattice to 4.40 Å using the cryoEM model of the cage (Extended Data Fig. 7) for molecular replacement (Fig. 2h). The experimentally determined crystal lattice is very similar to the computationally designed model both in overall structure and at the individual crystal lattice interfaces, with all-atom RMSDs of 2.80 Å over the full cage (Supplementary Fig. 6d), 3.00 Å over the full dihedral (Supplementary Fig. 6e) and 3.77 Å over two complete interacting cages (Supplementary Fig. 6f). The lower resolution of *F*4_1_32–2 than *F*4_1_32–1 likely reflects its intrinsic flexibility at the crystal contacts; the T32–15 cage cryoEM map had a lower local resolution (4–5 Å) at the periphery of the helical fusion region than the central part of the cage (Extended Data Fig. 7). With both the components and the interfaces designed entirely de novo, the *F*4_1_32–2 crystal is a macroscopic protein material computationally designed with high accuracy from the ground up.

## Design and characterization of *I*432–1 crystals

To investigate whether our crystal design approach could be applied to a broader range of space groups, we sought to design *I*432 crystals using designed octahedral cages to occupy the constituent Wyckoff sites (Fig. 2i). We designed cage O43–2 from a hyperthermophilic TIM barrel trimer (Protein Data Bank (PDB) accession number 1WA3) and a de novo designed helical repeat protein tetramer (tpr1C4_2)^33^ (Supplementary Fig. 10d); the 1WA3 trimer satisfies the design rule for presenting accessible secondary structures for the design of crystal contacts (Extended Data Fig. 1b). The two components could be readily expressed and purified separately, and they assembled in vitro to form cages at high yield, as evidenced by IMAC pull-down followed by SDS–PAGE analysis (Supplementary Fig. 10e), SEC (Extended Data Fig. 2i), nsEM (Extended Data Fig. 2j,k and Supplementary Fig. 10f) and SAXS (Extended Data Fig. 2l). One of the six ordered designs, O43–2-D3–6, formed rhombic dodecahedral shaped crystals (*I*432–1), the expected Wulff polyhedral crystal habit for body-centred cubic crystals^37^, overnight after in vitro mixing (Fig. 2j inset and Extended Data Fig. 6g). The crystals were further characterized by cryoEM (Fig. 2j and Extended Data Fig. 6i),SAXS(Fig. 2k) and X-raycrystallography (Fig. 2l). Indexing of the SAXS profile of *I*432–1 crystals indicated the *I*432 space group had a unit cell edge length of 237 Å, close to the designed lattice model (233 Å, Fig. 2k) with no sign of any mixed phases. Switching the purification tag to a chain terminus further from the crystal contact interface resulted in better-diffracting crystals (*I*432–1-CC). We solved the structure by X-ray crystallography at 3.66 Å in the designed *I*432 space group (Fig. 2l, slightly higher resolution than *I*432–1 crystals solved at 3.98 Å). The all-atom RMSD between the crystal structure and the computational design model was 1.34 Å over the full dihedral interface (Supplementary Fig. 6h) and 4.08 Å over two complete neighbouring cages (Supplementary Fig. 6i). In solving the designed crystal structure, we also validated the structure of the O43–2 cage, which has an all-atom RMSD to the cage design model of 1.91 Å (Supplementary Fig. 6g), providing an example of how crystal design can help address challenges in structural biology.

## Engineering the crystal lattice parameter

**Extended Data Fig. 8 |:**
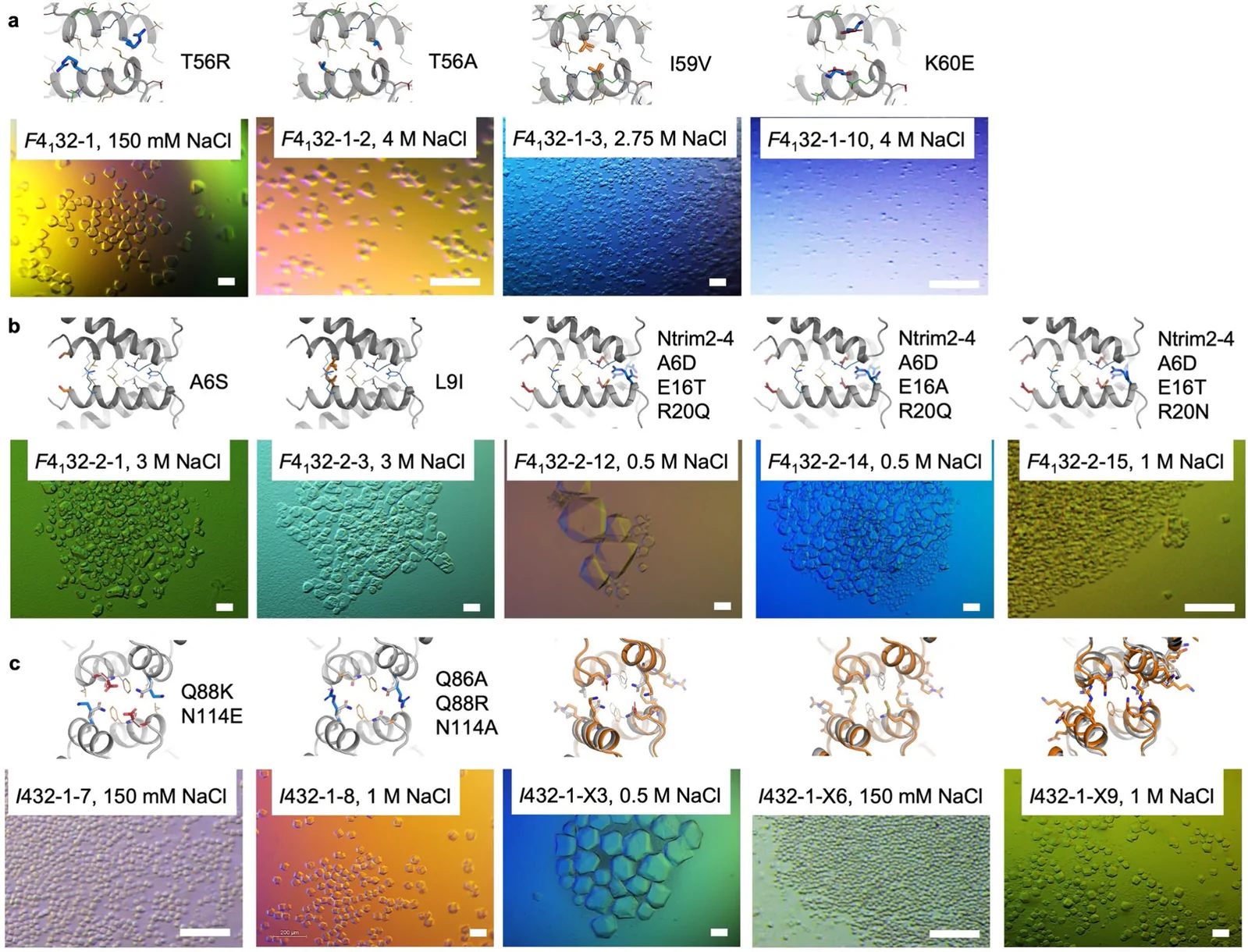
Tuning the crystallization behavior of designed crystals by mutagenesis. **a**, Mutations to the F4_1_32–1 crystals. **b**, Mutations of F4_1_32–2 crystals. **c**, Mutations and redesigns (orange) of I432–1 crystals. Top panels, crystal interface models based on X-ray structure. Interface side chains are hypothetically placed to demonstrate mutation sites. Bottom panels: optical micrographs of representative crystallization results. Scale bars, 100 µm.

**Extended Data Fig. 9 |:**
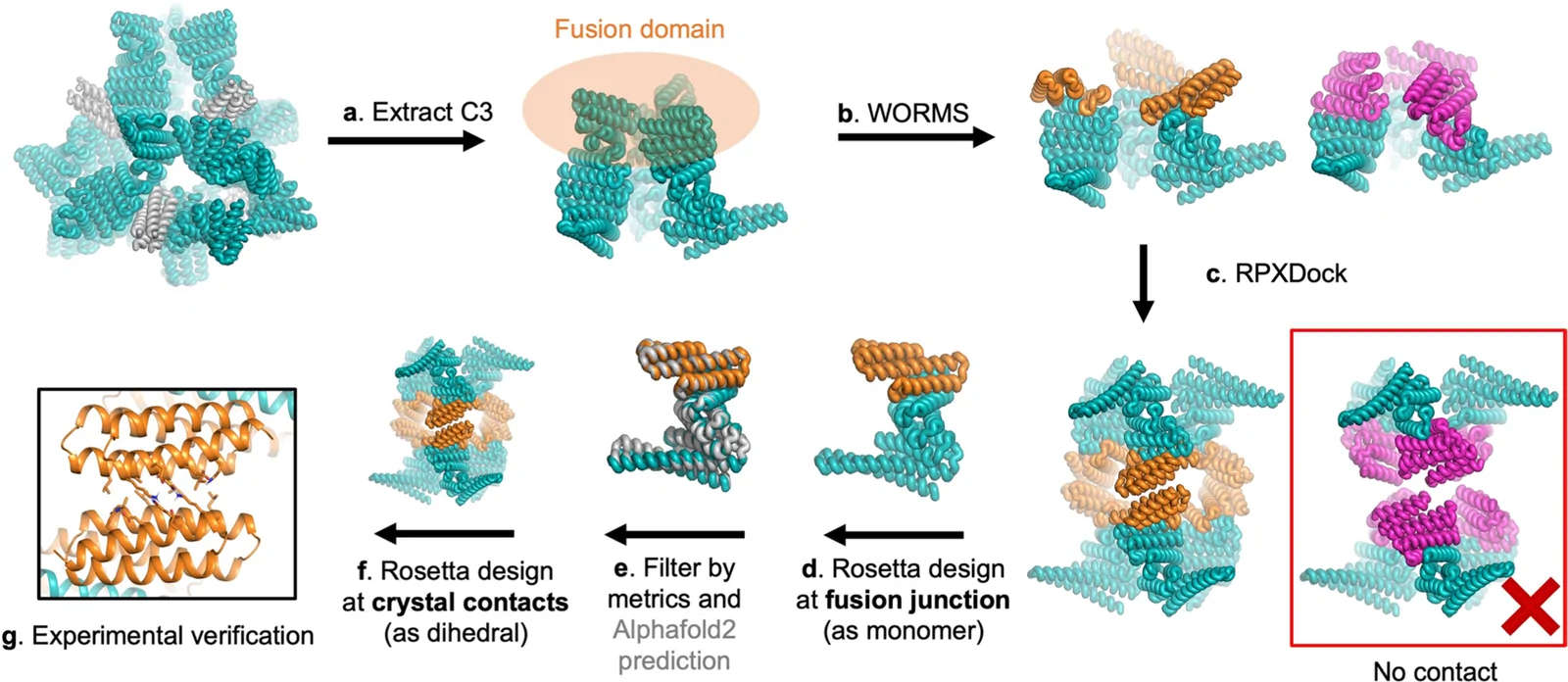
Design pipeline for engineering crystal unit cell dimension. The crystal contact of the F4_1_32–2 crystal was redesigned with different DHR arm fusion. See Methods for the details of step a-g.

**Fig. 3 |:**
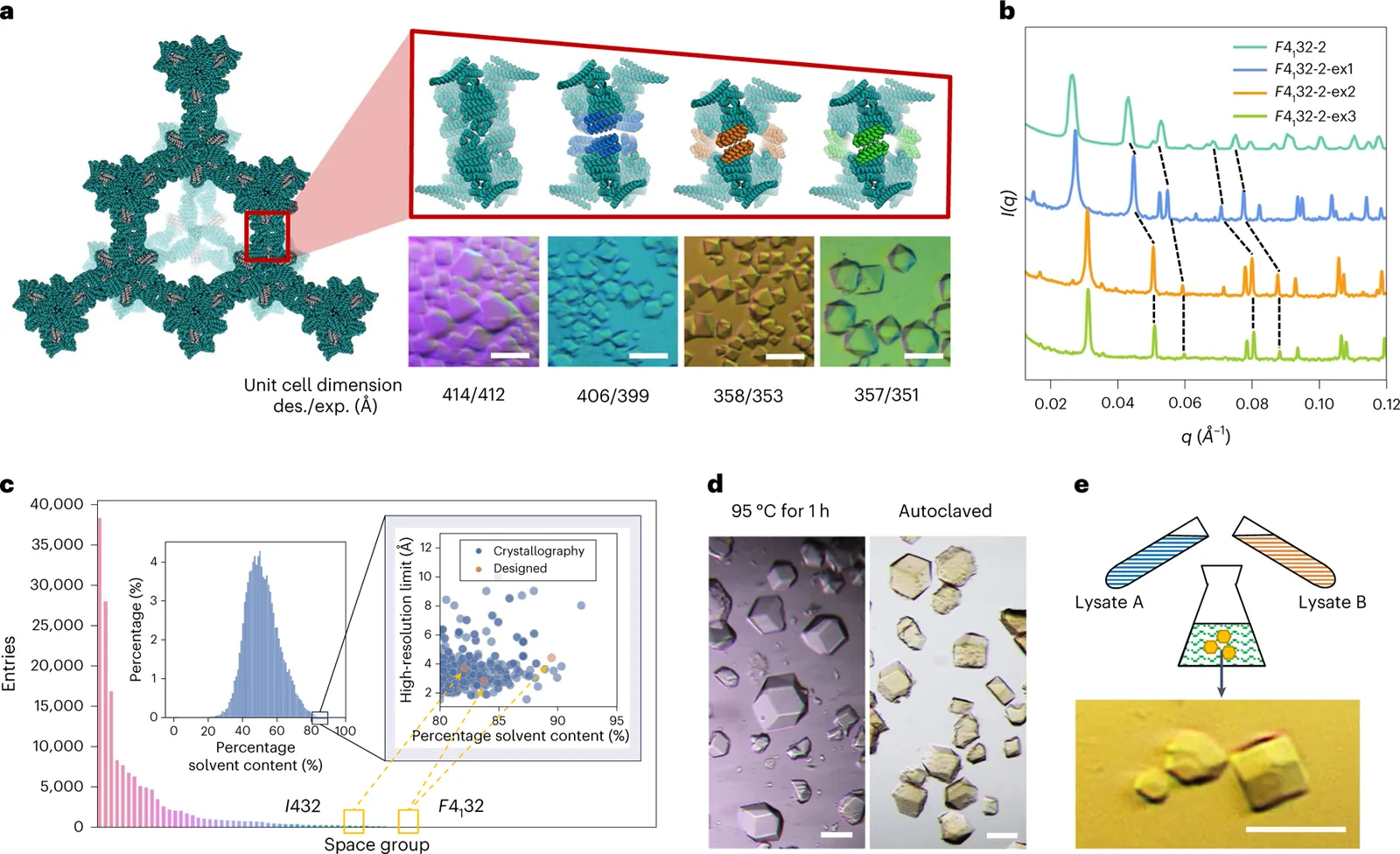
Engineering crystal properties. **a**,**b**, Tuning the unit cell dimensions of the *F*4_1_32–2 crystals by design. **a**, Design models and optical micrographs. Scale bars, 50 µm. **b**, SAXS profile. Peaks of the same index are connected by dashed lines. **c**, Space group distribution over all crystals in the PDB. Inset, distribution of the crystal solvent content with an enlargement of the high solvent content region versus crystal resolution. Crystals designed in this paper are highlighted in orange. **d**, *I*432–1-CC crystals incubated at 95 °C for 1 h (left panel) and autoclaved at 121 °C, 13 psi for 40 min (right panel). Scale bars, 100 µm. **e**, *I*432–1-CC crystals formed overnight by mixing of *E. coli* lysates. Scale bar, 50 µm. Des., designed.

An attractive property of our designed systems is that since the atomic structure is specified computationally and then genetically encoded, the properties of the crystals can be systematically modulated by further design. As a first illustration of this, we set out to tune the unit cell parameters and, thus, the porosity of the *F*4_1_32–2 crystal. We varied the length of the repeating arms^38^ on the C3 component of the *F*4_1_32–2 crystal (Fig. 1b) and redesigned the resulting new crystal contact interfaces, while holding the oligomer and cage assembly interfaces fixed (Fig. 3a, left and top panels, Extended Data Fig. 9 and Methods). We obtained three new *F*4_1_32 designs that readily formed crystals (Fig. 3a, lower panels) with predicted lattice parameters ranging from 351 to 412 Å. The lattice parameters of the actual crystals were determined from a SAXS profile (Fig. 3b), and they were remarkably close to the design values (Fig. 3a, bottom row). Further, we also observed that the solubility of the components and the crystallization behaviours could be systematically tuned (Supplementary Tables 1 and 2, Extended Data Fig. 8 and Supplementary Figs. 12 and 13) through interface residue substitutions; in contrast with natural crystals this often leads to unpredictable crystallization outcomes^38^.

## Crystallization behaviour and properties of the designed crystals

Compared with crystals found by screening^39^, our designed protein crystals have distinct crystallization behaviours and physical properties, as summarized in Extended Data Table 1. The crystals designed in this study occupy a unique structural space with remarkable thermal stability. They crystallize in the highly symmetrical cubic space groups (*F*4_1_32 and *I*432, Fig. 3c) and have among the highest solvent content of any in the PDB (as high as 90%, fifth highest among more than 160,000 recorded entries, Fig. 3c inset). Despite their high solvent content, the crystals exhibited high thermostability. *I*432–1-CC remained intact after 1 h of incubation at 95 °C and even after being autoclaved at 121 °C, 13 psi for 40 min (Fig. 3d), whereas crystals *F*4_1_32–1 and *F*4_1_32–2 were stable at up to 65 °C and 85 °C, respectively (Supplementary Fig. 14). Crystals found by screening usually require covalent crosslinking to achieve such stability^40^. The designed crystal assembly is sufficiently robust that it can occur in complex mixtures: the two components of *I*432–1-CC are produced at high levels when expressed separately (~0.1 mg protein per ml of *Escherichia coli* culture), and the *I*432–1-CC crystals formed with high yield upon mixing of crude cell lysates containing the two proteins (Fig. 3e and Supplementary Fig. 15a–c). This robustness enables scaled-up production and purification by crystallization: to illustrate this we developed a crystal production process using cleared lysates from cultures produced in 4 l bioreactors with an estimated yield of 340 mg of crystals per litre of culture (Supplementary Fig. 15d–f).

## Gold nanoparticle superlattices scaffolded in the designed crystals

**Fig. 4 |:**
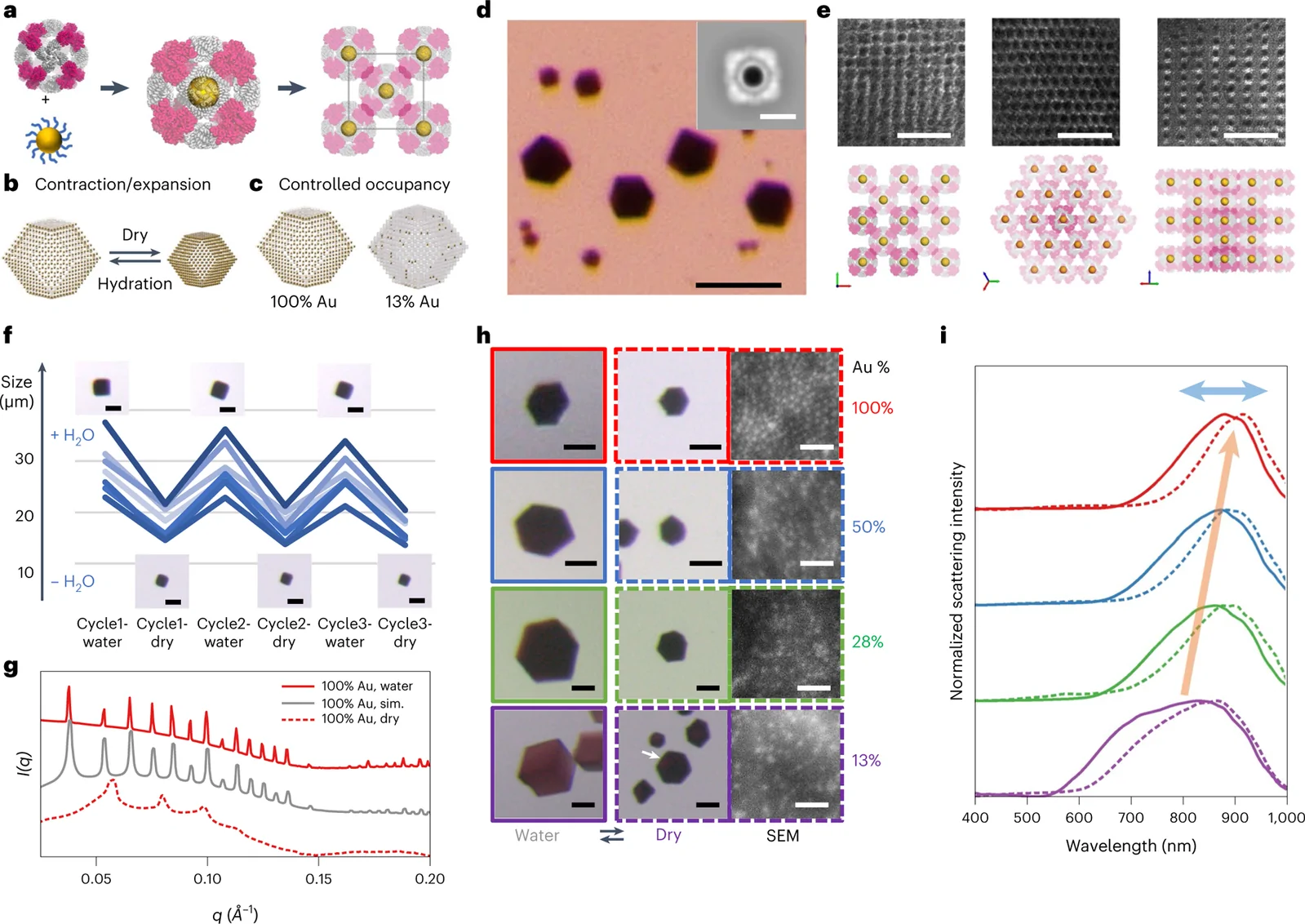
Scaffolding of 3D AuNP superlattices using the designed protein crystals. **a**, 5 nm AuNPs are encapsulated inside the *I*432–1-CC cages by a metal coordination interaction (Supplementary Fig. 16). The AuNP-encapsulated cages further self-assembly into designed *I*432–1 crystal lattices. **b**, AuNP superlattice single crystals exhibit reversible contraction and expansion during drying and rehydration cycles. **c**, Occupancy can be controlled for AuNP superlattice single crystals. 100% and 13% AuNP encapsulations are shown. Crystal scaffolds are in grey and AuNPs are drawn as golden spheres. **d**, Optical micrograph of crystals with AuNP encapsulation. Scale bar, 50 µm. Inset, representative 2D nsEM class average of AuNP-encapsulated O43–2 cage. Scale bar, 10 nm. **e**, NsEM micrographs (top panel) and 2 × 2 × 2 unit cell crystal model (bottom panel) of different crystals facets: ⟨100⟩ (left), ⟨111⟩ (middle) and ⟨110⟩ (right). Scale bars, 50 nm. **f**, Dehydration and rehydration cycles of AuNP superlattice single crystals. The size changes of seven crystals were measured and plotted.Representative optical micrographs of the crystals are shown for each hydration state. **g**, Experimental SAXS profiles of hydrated (solid red line) and dried (dashed red line) crystals with 100% AuNP encapsulation. Simulated patterns (solid grey line) of a superlattice formed by 5.6 nm diameter AuNPs with a lattice parameter of 236.3 Å closely match the experimental results. **h**, Optical micrographs of crystals with different AuNP encapsulation ratios (13%, 28%, 50% and 100%) in hydrated (solid lines) and dried states (dashed lines). Scale bars, 20 µm. Representative scanning electron microscope (SEM) images of dried crystals are shown on the right. Scale bars, 50 nm. **i**, Representative hyperspectral dark-field scattering spectra for crystals with AuNPs. The orange arrow shows the redshift of the scattering peak with increasing AuNP encapsulation ratio. The blue arrow illustrates the reversible shift of the scattering peak between dried crystals and hydrated crystals (dried crystals are redshifted). Colours and line styles correspond to samples in **g**.

We sought to take advantage of the robust self-assembly and large open volume of the designed crystals to template inorganic nanoparticle 3D superlattices^41–47^ (Fig. 4a). The *I*432–1-CC cages have inward-facing His-tags, and low-salt incubation of the cages with nickel (II) nitriloacetic acid (Ni-NTA) decorated gold nanoparticles led to the encapsulation of the AuNPs (Fig. 4d and Supplementary Figs. 16 and 17). These cages then assemble to form 20–50 µm single-crystalline nanoparticle superlattices (Fig. 4d,e and Supplementary Fig. 18b). The SAXS profile of the nanoparticle superlattice matches the prediction derived from the computational design model, with sharp peaks and high angle scattering that indicate considerable long-range order (Fig. 4g).

We explored two strategies to modulate the superlattice properties. First, we crosslinked the crystals to enable contraction and expansion during drying and rehydration (Fig. 4b). Second, we varied the AuNP encapsulation ratio to tune the superlattice occupancy (Fig. 4c). Both strategies adjusted the interparticle distance distribution, thus influencing the optical properties of the material. Glutaraldehyde crosslinked crystals^40^ were stable in water and underwent reversible contraction and expansion cycles (Fig. 4f). A 40% contraction, corresponding to an 80% volume change, was observed by optical microscopy and was reversible upon hydration (Supplementary Fig. 19). Appreciable long-range ordering was retained for dried-state crystals, as evidenced by multiple observed SAXS peaks (Fig. 4g and Supplementary Fig. 20). We varied the AuNP encapsulation ratio by assembling crystals from cages with and without AuNP encapsulation mixed at specified ratios (Supplementary Figs. 21–26). Finally, we combined the modulations by varying the hydration state and the AuNP encapsulation ratio simultaneously (Fig. 4h).

The hydration-dependent contraction and expansion and the varying AuNP encapsulation ratios impacted the near-infrared scattering profile of the superlattices (Fig. 4i). For superlattices with different AuNP encapsulation ratios, dehydration-induced shrinkage led to reversible redshifts in the photonic scattering spectra (Fig. 4i, solid lines versus dashed lines, and Supplementary Figs. 27–31). Regardless of their hydration state, increasing the AuNP encapsulation ratio from 13% to 100% resulted in 50–100 nm redshifts of photonic scattering peaks (Fig. 4i and Supplementary Fig. 29). By simultaneously varying the hydration state and the encapsulation ratio, the optical scattering profiles of the superlattices could be tuned over a broad range (Fig. 4i). The tunable refractive indices of the AuNP superlattices were modelled using Maxwell–Garnett effective medium theory, which describes the optical properties of small quasi-plasmonic nanoparticles in a finite-sized superlattice^48,49^. The simulations recapitulate the spectroscopic shifts accompanying variations in the AuNP encapsulation ratio and hydration state; nanoparticle density within a superlattice modulates the effective refractive index and, thus, the crystal’s optical properties (Supplementary Fig. 32). Thus, our designed protein crystals enable the patterning of crystalline nanoparticle arrays and tuning of their macroscopic properties.

## Conclusions

The ability to design protein crystallization and create macroscopic 3D protein materials with high accuracy by self-assembly is a substantial advance for protein design. Key to this design success is the hierarchical design of individually orthogonal interfaces that progressively constrained the system DOFs. Our approach goes beyond previous work^5–12^ on generating crystal lattices from cages by enabling the programming of the crystal lattice geometry with atomic accuracy through designed side-chain–side-chain interactions, and by fully encoding the crystal assembly information in the primary sequence of the component proteins. Computationally designed protein crystals are a new class of biomaterials that can be prepared and purified at large scale, are stable under extreme conditions (for example, at 95 °C and in lysate) and provide robust and tunable scaffolds for nanoparticle-superlattice-based optical materials. The exceptionally high porosity of the crystals with channels both within and between the cages, the tunability of the lattice dimensions and hence the inter-cage channel spacings, and the ability to trigger crystallization simply by mixing components, enable a wide range of biotechnology and medical applications.

## Methods

### Cage docking and design

A library of cyclic oligomer scaffolds (C2, C3 and C4) from crystal structures deposited in the PDB (http://www.rcsb.org/pdb/) and from previous de novo designs^32–35^ were docked into tetrahedral and octahedral cages (T32, T33, O32, O42 and O43). Cage dockings were carried out by RPXDock, which used the hierarchical sampling of residue pair transform scoring to find high designability docking^30^. The top 100 to 500 dockings of each symmetry were sequence designed by Rosetta^36^ using a protocol based on two-component protein–protein interface design methods implemented within the RosettaScripts framework^50^. Either beta_nov16 or a clash-fixed score function was used during the design. The protein–protein interfaces were designed with rigid protein backbones by packing rotamers with layer design restrictions at interacting residues. All cage designs were filtered by a number of metrics evaluating the designed cage interface, including methionine count ≤5, shape complementarity >0.6, the change in the change in the Gibbs free energy (delta delta *G* or ddG) less than −20 kcal mol^−1^, solvent-accessible surface area <1,600, clash check ≤2 and unsatisfied hydrogen bonds ≤2, before visual inspection of the hydrophobic packing for more than two pairs of intersected hydrophobic side chains. In the final filtering, the cages were placed into the corresponding Wyckoff sites in the target crystal lattice and the cage backbone was inspected to ensure there would be sufficient secondary structure interactions between cages (Extended Data Fig. 5, before the extraction of dihedrals). Cage designs with no or little interacting secondary structures were not verified experimentally.

### Crystal docking and design

Ten previously designed cages (T3–10, T32–28, T33–09, T33–15, T33–21, T33–23, T33–28, T33–31, T33–51 and T33–53)^25–27^ and experimentally verified new cages were used for the docking to target the *P*23, *P*432, *P*4_2_32, *I*432, *F*23, *F*432 and *F*4_1_32 space groups. Dockings were performed in Pymol^51^ by alignment and translations. For the docking of the *F*4_1_32 space group, two tetrahedral cages (TET-1 and TET-2) were aligned with every C2 symmetry axis along the *x*, *y* and *z* coordinate axes and centre of mass at the origin of the coordinate (Extended Data Fig. 5a, left panel). While keeping TET-1 fixed at the origin, TET-2 was rotated along the *z* axis for 90° and translated along the (1, 1, −1) direction to dock with TET-1 (until the distance between the backbones of the two cages was less than 6 Å with 1 Å increment translations, Extended Data Fig. 5a, middle panel). The hexamer between the two cages with D3 dihedral symmetry was extracted and aligned with its C3 axis along the *z* axis and its C2 axis along the *x* axis and with its centre of mass at the origin (Extended Data Fig. 5a, right panel). The docking of the *I*432 space group was similar between two octahedral cages (OCT-1 and OCT-2), except OCT-2 was translated only without any rotation (Extended Data Fig. 5b). In both cases, the D3 assembly units contained the crystal contact and were used for Rosetta sequence design. In the RosettaScripts framework^50^, input monomers were symmetrized into D3 dihedrals, and then we sampled the interface distances by translation along the *z* axis (dihedral axis) within ±1.5 Å of the docked conformation without a rotational DOF. For each translation, the dihedral interface was designed with rigid protein backbones by packing a rotamer with layer design restrictions at interacting residues. Then, designs were filtered by ddG <0, solvent-accessible surface area >200, clash check ≤2 and unsatisfied hydrogen bonds ≤2 before being visually inspected for hydrophobic packings. Working crystal designs usually have one to three pairs of intersecting hydrophobic side chains at each C2 branch of the D3 dihedral. The DOFs after each design step were as follows. Two cyclic oligomers have eight DOFs, as each monomer has four DOFs to assemble into cyclic oligomer^33^. The co-assembly of the cage has four, as each of the two cyclic oligomers has two DOFs^26^. The final crystal has only one DOF to be the translation between cages.

### Redesign of crystal unit cell dimension

To design the unit cell dimension of the *F*4_1_32–2 crystals, the DHRs at the periphery of the cage were engineered with new fusions to alter the cage spacing in the crystal lattice. The C3 symmetry unit was extracted from the cryoEM model of the T32–15 crystal (Extended Data Fig. 9a) and fused with a library of verified DHRs by the WORMS protocol^35^ (fusion region defined as residue 1–195, away from the oligomer and cage assembly interfaces; Extended Data Fig. 9b). The fusions with a new backbone were docked by RPXDock^30^ in D3 symmetry with only a translational DOF along the C3 axes of the dihedral (Extended Data Fig. 9c). The docking angle for crystal propagation was pre-implemented before C3 extraction, and fusions showing no contact by docking were filtered from the downstream design. The monomer of the docked dihedral was extracted and designed at the fusion region by Rosetta by packing rotamers with layer design restrictions and filtered by shape complementarity >0.5 and alanine percentage <40% at the junction (Extended Data Fig. 9d). The fusion designs were then filtered with AlphaFold2 predictions (model 4, RMSD < 2 design model versus prediction and predicted local distance difference test > 85; Extended Data Fig. 9e). As the final design step, filtered fusions were Rosetta designed in D3 symmetry by the same protocol as described above for crystal contacts (Extended Data Fig. 9f). Both the designs of the fusion junction and the crystal contacts were experimentally verified at the same time by crystal assembly (Extended Data Fig. 9g).

### Rotation correction for design and crystal model comparison

Because of the minimization of the virtual connections (‘jumps’) between the symmetry subunits in Rosetta design, the D3 dihedral design outputs could slightly deviate from the restricted rotation constrained by crystal symmetry. To construct a design model of the crystal unit cell to compare with the crystallographic model, rotation-corrected dihedral models were prepared by applying the same dihedral axis displacement as in the design model, while keeping the rotation angle fixed (before minimization). The rotation-corrected dihedral models were then used to propagate the design model of the cage into one crystal unit cell by alignment.

### Clash-fixed Rosetta score function

We observed that designs made with the beta_nov16 score function tend to have higher levels of steric clashing than observed in high-resolution crystal structures of native proteins. The parameters in the Rosetta score function were refitted to reduce this steric-clashing propensity, resulting in the ‘clash-fixed’ score function. We are preparing a manuscript that describes this problem and the new score function in more detail. The files in the ‘0_clash-fixed_scorefunction’ folder of the Supplementary Material encode all the parameter changes and can be used to implement the new score function in design.

### Protein expression and purification

Synthetic genes were optimized for *E. coli* expression and purchased from IDT (Integrated DNA Technologies) as plasmids in the pET29b vector with a hexahistidine affinity tag. For cage screening, genes for the two cage components were joined together by another ribosome binding site domain in between (gene sequence: TAAAGAAGGAGATATCATATG). The hexahistidine tag was added onto only one of the components. Plasmids were cloned into BL21* (DE3) *E. coli* competent cells (Invitrogen). Single colonies from an agar plate with 100 mg l^−1^ kanamycin were inoculated in 50 ml of Studier autoinduction media^52^, and the expression continued at 37 °C for over 24 h. The cells were harvested by centrifugation at 4,000*g* for 10 min, and resuspended in 35 ml lysis buffer of 300 mM NaCl, 25 mM tris pH 8.0 and 1 mM phenylmethylsulfonyl fluoride. After lysis by sonication and centrifugation at 14,000*g* for 45 min, the supernatant was purified by Ni^2+^ IMAC with Ni-NTA Superflow resins (Qiagen). Resins with bound cell lysate were washed with 10 ml (bed volume 1 ml) of washing buffer (300 Mm NaCl, 25 mM tris pH 8.0 and 60 mM imidazole) and eluted with 5 ml of elution buffer (300 mM NaCl, 25 mM tris pH 8.0 and 300 mM imidazole). Both soluble fractions and full cell cultures were checked by SDS–PAGE. Soluble designs with the correct molecular weight and cage designs with pull-down showing two separate bands were further purified by SEC. Concentrated samples were run in 150 mM NaCl and 25 mM tris pH 8.0 on a Superose 6 Increase 10/300 gel filtration column (Cytiva). SEC-purified designs were concentrated by 10 K concentrators (Amicon) and quantified by ultraviolet absorbance at 280 nm before further assembly and characterization.

### Crystallization of designs

For all crystallization experiments, the concentration of protein monomers was specified for simplicity. For example, 50 µM component A represents 50 µM of component A monomer, giving 50/*n* µM of the oligomer depending on the *n*-fold of A (*n* = 2, 3, 4). A 50 µM cage represents a cage assembled from 50 µM of component A and 50 µM of component B, giving a 50/12 = 4.2 µM cage assembly for tetrahedral cages (12 copies of each component) or a 50/24 = 2.1 µM cage assembly for octahedral cages (24 copies of each component).

Since our crystals were designed to crystallize with no dependence on specific precipitants or additives, all crystallization experiments were screened in NaCl solution. As initial tests, cage components were mixed in 150 mM NaCl and 25 mM tris pH 8.0 from overnight to days to observe cage or crystal assembly. For designs that did not crystallize, the mixed solution was set up for hanging drops with 0.5 M to 5 M NaCl. For each screened NaCl concentration, NaCl solutions of the target concentration were used as reservoir solution, and the hanging drop was a mixture of 2 µl of the solution of the mixed components and 2 µl of the reservoir solution. For some designs, like *I*432–1-CC, the hanging drop was set up with only the cage solution (no extra NaCl) against the NaCl reservoir solution. Crystal trays were checked for crystallization for up to two weeks and further optimized with the NaCl concentration. Designs were also examined for batch crystallization by mixing cages and NaCl solution at a 1:1 volumetric ratio according to the hanging drop conditions. Crystallization conditions for all designs are summarized in Supplementary Table 1.

### Thermal stability of designed protein crystals

The thermal stability study used *F*4_1_32–1 crystals by mixing, *F*4_1_32–2 crystals by hanging drop (diluted in 1.5 M NaCl) and *I*432–1-CC crystals by hanging drop (diluted in 0.5 M NaCl). Further, 0–20 µl of the crystals were intubated in a T100 Thermal Cycler (Bio-Rad) at 55 °C, 65 °C, 75 °C, 85 °C and 95 °C for 1 h. Crystals were checked before and immediately after incubation (within 5 min) under an optical microscope by pipetting the solution and applying it to a siliconized glass slide. Autoclaving experiments used 20 µl of crystal solution in an opened 1.5 ml Eppendorf tube at 121 °C, 13 psi for 40 min.

### Purification of *I*432–1-CC crystals from lysate

After sonication and centrifugation of the clarified lysate (Protein expression and purification), the two components of *I*432–1-CC crystals were mixed at 1:1 volumetric ratio. Then, 20–50 µm of crystals appeared in solution after overnight incubation. The crystals were collected by centrifugation at 4,000*g* for 10 min and washed twice with 300 mM NaCl and 25 mM tris pH 8.0. For recrystallization, the centrifuged crystals were dissolved in water, and set up as hanging drops in 0.5 M NaCl. Despite the high thermal stability of the designed crystals, recrystallization demonstrated that crystal assembly was reversible by controlling the ionic strength, providing flexibility for storage and applications.

### Scaffolding of 3D AuNP arrays

The constituent cage of *I*432–1-CC crystals has an interior void of ~10 nm diameter. The His-tags from both components face towards the inside of the cages to provide sites for host–guest interactions. We used the Ni-NTA His-tag interaction (*K*_D_ ≈ 10^−13^ M at pH 8.0)^53^ to guide the encapsulation of 5 nm AuNPs into the interior of the O43 cages. Then, 50 µM *I*432-CC-1 cages were assembled overnight in 150 mM NaCl and 25 mM tris pH 8.0. Next, 10 µl of cages, 2 µl 1 M imidazole and 88 µl of 5 nm AuNPs functionalized with Ni-NTA chelates (0.5 µM in 50 mM MOPS, pH 7.9, Nanoprobes) were mixed for nanoparticle encapsulation. Because of the low ionic strength (~15 mM NaCl) and excess amount of AuNPs (>4 times excess), the cages transiently self-assembled with oligomer pieces being disassembled and assembled^54^, giving the AuNPs time to bind with a partially disassembled cage and become encapsulated. The encapsulation was characterized by nsEM by diluting the solution 10 times in 150 mM NaCl, 25 mM tris pH 8.0 and 300 mM imidazole right before imaging. It took about two weeks for more than half of the cages to have encapsulated AuNPs. For purification, 15 µl of 1 M NaCl and 30 µl of 1 M imidazole were added to the solution right before centrifugation at 14,000*g* for 30 min. The pellets of free AuNPs and AuNPs encapsulated in cages were redispersed by 100 µl 150 mM NaCl, 25 mM tris pH 8.0 and 300 mM imidazole and centrifuged again at 14,000*g* for 30 min. The final pellet was dispersed in 20 µl of 150 mM NaCl, 25 mM tris pH 8.0 and 300 mM imidazole. The solution, now containing O43 cages with encapsulated and free AuNPs (which did not assemble with a cage), was set up for crystallization by hanging drop of 4 µl solution against 0.5 M NaCl. We also found that cages of *I*432–1 crystals with His-tags facing towards the outside of the cage co-assembled with the AuNPs into polycrystalline binary lattices upon mixing with the AuNPs (Supplementary Fig. 18a).

### Determination of crystal structure

Data were collected during synchrotron irradiation either at the Advanced Photon Source (APS) on beamline 24-ID-C or at the Advanced Light Source (ALS) on beamline 8.2.1/8.2.2. X-ray intensities and data reduction were evaluated and integrated using either X-ray diffuse scattering^55^ or HKL3000 (ref. 56) and merged and scaled using Pointless/Aimless in the CCP4 program suite^57^. Structure determination and refinement starting phases were obtained by molecular replacement using Phaser^58^ using the design model for the structures. Following molecular replacement, the models were improved using phenix.autobuild^59^; efforts were made to reduce model bias by setting rebuild-in-place to false, and using simulated annealing and prime-and-switch phasing. Structures were refined in Phenix^59^. The models were built using Coot^60^. The final model was evaluated using MolProbity^61^. The collected data and refinement statistics are recorded in Supplementary Table 2. The final structures were deposited in the PDB (http://www.rcsb.org/) under accession numbers 8CUU, 8CUV, 8CUW, 8CUX, 8CWS, 8CUS, 8CUT, 8CWZ and 8FAR.

### Transmission nsEM

Cage fractions from SEC traces or cages by in vitro mixing were diluted to 0.5 µM (monomeric component concentration) for nsEM characterization. For the crystal samples, crystals were first crushed into smaller pieces under an optical microscope by cryo-loops before applying them onto the electron microscope grids. A drop of 6 µl sample was applied on negatively glow-discharged, formvar/carbon-supported 400-mesh copper grids (Ted Pella, Inc.) for more than 2 min. A grid was blotted and stained with 3 µl of uranyl formate, blotted again and stained with another 3 µl of uranyl formate for 20 s before final blotting. We used 0.75% and 2% uranyl formate for different samples. The screening was performed on either a 120 kV Talos L120C transmission electron microscope (TEM; Thermo Scientific) or a 100 kV Morgagni M268 TEM.

### NsEM image processing

All nsEM datasets were processed by CryoSparc software^62^. Micrographs were imported into the CryoSparc web server, and the contrast transfer function was corrected. Around 100 particles were manually picked and classified in two dimensions. Selected classes were used as templates for particle picking in all images. All the picked particles were two-dimensionally classified for 20 iterations into 50 classes. Particles from selected classes were used for building the ab initio initial model. The initial model was homogeneously refined using C1 and the corresponding T/O symmetry.

### CryoEM sample preparation

To prepare the T32–15 cage samples, 2 µl of 5.3 mg ml^−1^ of the T32–15 cages in 150 mM NaCl and 25 mM tris pH 8.0 was applied to glow-discharged C-flat holey carbon grids. Vitrification was performed using manual blotting at ambient temperature and humidity. To prepare crystal samples, the crystals were first crushed into smaller pieces under an optical microscope by cryo-loops right before freezing. Samples in the corresponding crystallization buffer were frozen using glow-discharged 1.2/1.3-T C-flat holey carbon grids and a Mark IV Vitrobot with a wait time of 5 s, a blot time of 7.5 s and a blot force of 0 before being immediately plunge frozen into liquid ethane.

### CryoEM data collection

T32–15 data were collected automatically using Leginon^63^ and used to control a ThermoFisher Titan Krios 300 kV TEM (Pacific Northwest Center for Cryo-EM Krios 1) equipped with a standalone K3 Summit direct electron detector^64^ and operating in super-resolution mode. Random defocus ranges spanned between −0.8 and −2.2 µm using image shift, with one-shot per hole and nine holes per stage move. Altogether, 4,202 videos were recorded with a pixel size of 0.5144 Å with a total dose of 50 e^−^/Å^2^. Individual images of crushed crystals were manually collected on either a ThermoFisher Glacios 200 kV TEM equipped with a K2 summit direct electron detector using serial electron microscopy or a ThermoFisher Talos L120C TEM equipped with a BM-Ceta camera using EPU 2.0.

### CryoEM data processing

All data processing was carried out in CryoSPARC^62^. The video frames were aligned using Patch Motion with an estimated B factor of 500 Å^2^. The maximum alignment resolution was set to 3. Outputs were binned to a final pixel size of 1.0288 Å per pixel by setting the output F-crop factor to ½. Defocus and astigmatism values were estimated using the Patch contrast transfer function with the default parameters. In total, 727,160 particles were picked in a reference-free manner using Blob Picker and extracted with a box size of 320 Å. An initial round of reference-free two-dimensional (2D) classification was performed in CryoSPARC using 100 classes and a maximum alignment resolution of 6 Å. The best classes—each comprising 207,495 particles—were then used for 3D ab initio determination using the C1 symmetry operator. These same 2D classes were next low-pass filtered to 20 Å and used as templates for a second round of particle picking using Template Picker, resulting in a new set of 964,346 particle picks, which were extracted with a box size of 320 pixels. Following another round of reference-free 2D classification, the best 511,464 particles were submitted for non-uniform refinement in the presence of T symmetry for a final estimated global resolution of 3.34 Å. Local resolution estimates were made in CryoSPARC using a forward scatter threshold of 0.143. 3D maps for the two half-maps, the final unsharpened map and the final sharpened map were deposited in the Electron Microscopy Data Bank under accession number EMD-27031.

### CryoEM model building and validation

The design mode of the T32–15 cage was used as an initial reference for building the final cryoEM structure. The model was manually edited and trimmed using Coot^60,65^. We then further refined the structure in Rosetta using density-guided protocols^66^. This process was repeated iteratively until convergence and high agreement with the map was achieved. Multiple rounds of relaxation and minimization were performed on the complete cage, which was manually inspected for errors each time. Throughout this process, we applied strict non-crystallographic symmetry constraints in Rosetta^67^. Residues 1–124 for each chain were truncated to the Cβ carbon due to the low resolution of this region and low confidence in the determination of side-chain rotamers. Phenix real-space refinement was subsequently performed as a final step before the final model quality was analysed using MolProbity^61^ and EM ringer^68^. Figures were generated using either UCSF Chimera^69^ or UCSF ChimeraX^70^. The final structure was deposited in the PDB under accession number 8CWY.

### SAXS of protein nanocages

Protein nanocage samples were re-purified by SEC in 150 mM NaCl, 25 mM tris pH 8.0 and 2% glycerol and concentrated using thoroughly washed small spin concentrators with a 10 K molecular weight cutoff. The flowthrough was used as blanks for buffer subtraction. Solution scattering measurements (shown in Extended Data Figs. 2, 3 and 4) were performed at the SIBYLS 12.3.1 beamline at the ALS in the Lawrence Berkeley National Laboratory. The SIBYLS beamline parameters and collection procedures have been previously described^71,72^. The beamline has a fixed sample-to-detector distance of 2 m. Samples were placed in a transmission geometry. The monochromatic 10 keV X-ray beam converges to a rectangular 0.5 × 1 mm^2^ beam at the sample and becomes a 100 × 100 µm^2^ beam at the detector. The area detector was a Pilatus 2 M with 172 × 172 µm^2^ pixels. SAXS data from the 2D area detector were integrated using beamline-specific software to create X-ray intensity versus momentum transfer one-dimensional curves (*I*(*q*)). A series of exposures, in equal sub-second time slices, were taken of each well: 0.3 s exposures for 10 s resulting in 32 frames per sample. For each sample, data were collected for two different concentrations to test for concentration-dependent effects. ‘Low’ concentration samples were ~1 mg ml^−1^ and ‘high’ concentration samples were ~5 mg ml^−1^. The data were processed using the SAXS FrameSlice online server. FoXS^73^ was used to compare design models with experimental scattering profiles and to calculate the quality of fit (*χ*) values. In this work, all *I*(*q*) profiles were plotted on a logarithmic scale for clarity.

### SAXS of crystals

All crystal structures in this work were confirmed primarily by SAXS. SAXS characterization was carried out at the 12-ID-B beamline of the APS at Argonne National Laboratory following a literature report^74^. The wavelengths of X-rays used at the 12-ID-B beamline was 0.9322 Å (13.3 keV), and the system was calibrated using silver behenate as a standard. Two sets of slits were used to define and collimate the X-ray beam, and parasitic scattering was removed using a pinhole. Typical exposure times of between 0.1 and 0.5 s were used. The scattered X-rays were collected with a charge-coupled device, and one-dimensional scattering profiles were obtained by radially averaging the images into scattering intensities, *I*(*q*), against the scattering vector magnitude, *q*.

To study hydrated and dehydrated states of crosslinked crystals with encapsulated AuNPs, solutions of microcrystals were sent to the SIBYLS beamline (Supplementary Fig. 20). The collection parameters were identical to those described in the SIBYLS solution studies of nanocages. However, the sample handling was changed to optimize collection for crystals in solution.

To fully contrast the lattice parameters of hydrated and dehydrated crystals, care was taken to ensure that results were collected for the exact same material in both states. For the hydrated samples, a solution of microcrystals was transferred from a storage container to the SAXS sample cell using a liquid handler. The SAXS sample cell was a 1 mm sheet of aluminium with a U-shaped cutout for receiving samples. A thin piece of mica (0.02 mm thick) was glued to each side over the cutout to contain the liquid inside the U-shape. The liquid handler transfer needles were thin enough so that they could be present inside the SAXS sample cell during data collection. Using a mixing command, which causes the needle to dispense and aspirate a small volume in quick succession, the liquid in the sample was continuously mixed during collection. Microcrystals passed back and forth in front of the beam rather than settling at the bottom of the sample cell, where X-rays might scatter from the sample cell. This process allowed the microcrystals to pass through the X-ray beam in many orientations and generate rings of X-ray intensity on the detector. To subtract parasitic scattering and sample cell contributions, a water sample was also collected. The SAXS from the water was subtracted from the sample.

For the dehydrated samples, the loaded sample cell was tilted on its side so that the microcrystals would settle on the mica, and it was left to dry overnight. The dried cell was then placed in the beam. SAXS generated from a single spot in the sample cell was not sufficient to generate rings. The sample cell was translated in the beam during collection. This process allowed the collection of SAXS data from more dehydrated crystals. Data from the full translation series were integrated together. SAXS from an empty sample cell was used for the background.

### Modelling of a protein crystal from SAXS data

One-dimensional SAXS profiles were simulated and indexed following a literature report^75,76^. Crystallographic symmetries and lattice parameters were determined by indexing powder diffraction patterns. The full-atom representation of protein cages was located into their corresponding Wyckoff positions of the lattice, from the smallest multiplicity site to the highest. The average crystalline domain size, Debye–Waller factor and microstrain parameter were empirically adjusted to fit the peak width and relative peak intensities across the entire pattern.

In Supplementary Fig. 33, the calculated form factor of each cage is shown in blue and intensity profiles are shown in red and black for simulated and measured profiles, respectively. For all three curves, simulated peaks at around the form factor minima deviate from the measured ones unlike other peaks. This might indicate that the form factor minima in the real samples are not as sharp as the ones calculated or that they may have been shifted slightly. This is possible when there is a size distribution of the cages, for example due to different hydration of the cages, or when there are diffuse scatterings due to orientational or positional defects. We did not neglect non-perfect random orientations of the crystals in solution, which may have caused some random peaks to be stronger than others.

### Hyperspectral dark-field microscopy

Hyperspectral dark-field scattering data cubes were collected on a Photon Etc. IMA system using a Nikon Ni-U upright microscope and a ×60 objective lens (Nikon, numerical aperture 0.6). Full-field scattered light was passed through a tunable volume Bragg grating filter with a step size of 4 nm and integration time of 1 s per step. The scattered light at each wavelength step was collected by a charge-coupled device camera (Thorlabs, 1501M-US-TE). The hyperspectral data cube was built by accumulating each wavelength slice from 400 to 1,000 nm. Scattering spectra were normalized by dividing by a white light reference spectrum and then background corrected. We used the Photon Etc. PhySpec 2.27 software for the calculation.

### Scanning electron microscopy

The SEM micrographs were obtained using TFS Apreo-S with Lovac Scanning Electron Microscope operating at 2 kV and 13 pA under the Immersion Use case. The Beam Deceleration module was on, and a negative 4 V (bias) was applied to the stage. The working distance was 3.00 mm, and the micrographs were collected by the T1 detector. Further analysis was done using ImageJ software.

### Effective medium theory

For small quasistatic plasmonic spheres, the interactions are primarily dipolar when they spaced by greater than a diameter and the volume fractions are relatively low (<20%)^48,49^. In these cases, the optical properties are described well by effective medium theory, which includes the metallic dielectric function and the background refractive index environment weighted by the volume fraction occupied.

The effective material properties can be described by an effective dielectric function $\varepsilon_{\text{eff}}$:

$$\frac{{\text{ε}}_{\text{eff}}-1}{{\text{ε}}_{\text{eff}}+2}=f\frac{\text{ε}-1}{\text{ε+2}}$$

where ε is the dielectric function of the material and f is the volume fraction of the metal, given by:

$$f=N_{\text{inclusions}}\frac{V_{\text{nanoparticle}}}{V_{\text{unit}\;\text{cell}}}$$

where $N_{\text{inclusions}}$ is the number of nanoparticle inclusions per unit cell, $V_{\text{nanoparticle}}$ is the volume of each nanoparticle and $V_{\text{unit}\;\text{cell}}$ is the total volume of interest, in this case, a single unit cell. Here, we include a small-size correction to the dielectric function of gold to account for the broadening and damping observed in small nanoparticles due to defects and surface-scattering events. Specifically, we applied an analytic adjustment to the finite mean free path of electrons that is related to the surface area and volume of a sphere^48,49,77,78^.

## Extended Data

**Extended Data Table 1 |:** Comparison of properties between designed protein crystals and crystals from screening

	Designed Crystals (This work)	Screened Crystals
Crystal Packing	Sequence encoded	Both sequence and screening condition dependent
Crystal Contacts	Distinct, hydrophobic, specific	Continuous, polar, nonspecific
Space Group preference	Highly symmetrical (Cubic)	Lower symmetry (monoclinic etc.)
Solvent Content	High (80–90%)	27–65% on average^79^
Porosity	Pores within and between cages	Usually uniform pores
Crystallization Concentration	Down to 0.2 mg/mL	Usually above 10 mg/mL
Crystallization Precipitants	Can crystallize upon mixing without additional precipitants	Usually need high concentration of precipitants
Thermal Stability	High (stable up to 95 °C)	Usually unstable above room temperature

Comparison of properties between designed protein crystals and crystals from screening.

## Supplementary Material

S1

sample scripts

models

The online version contains supplementary material available at https://doi.org/10.1038/s41563-023-01683-1.

## Data Availability

All data are available in the main text or as supplementary materials. Design scripts examples and design models (cages including T32–15, T32–15-6H, O43–2, T33-ECY54, T33-ECY55, T33-ECY59, T33-ECY66, T33-ECY67, O43-UWN453, O43-ZL1, O43-ZL7 and O32-ZL4, and crystal contact dihedrals of *F*4_1_32–1-0, *F*4_1_32–2, *I*432–1, *F*4_1_32–2-ex1, *F*4_1_32–2-ex2 and *F*4_1_32–2-ex3) are available in the supplementary information files and through Zenodo^79^. SAXS and hyperspectral data are available as source data files. Crystallographic datasets have been deposited in the PDB (accession codes 8CUU, 8CUV, 8CUW, 8CUX, 8CWS, 8CUS, 8CUT, 8CWZ and 8FAR). CryoEM maps and corresponding atomic models have been deposited in the PDB (accession codes 8CWY and 8SZZ) and the Electron Microscopy Data Bank (accession codes EMD-27031 and EMD-40926). Source data are provided with this paper.
