# Supplementary material for: Accurate computational design of three-dimensional protein crystals
Source: Nat Mater. Author manuscript; Available in PMC 2025 Jul 11. (PMC12253981; doi:10.1038/s41563-023-01683-1)
Supplement: Supplementary file 1 [file NIHMS2005979-supplement-S1.pdf]

nature materials

Article https://doi.org/10.1038/s41563-023-01683-1

Accurate computational design of three-dimensional protein crystals

In the format provided by the authors and unedited

Table of Contents

Supplementary Figures 1-33
Supplementary Tables 1-7

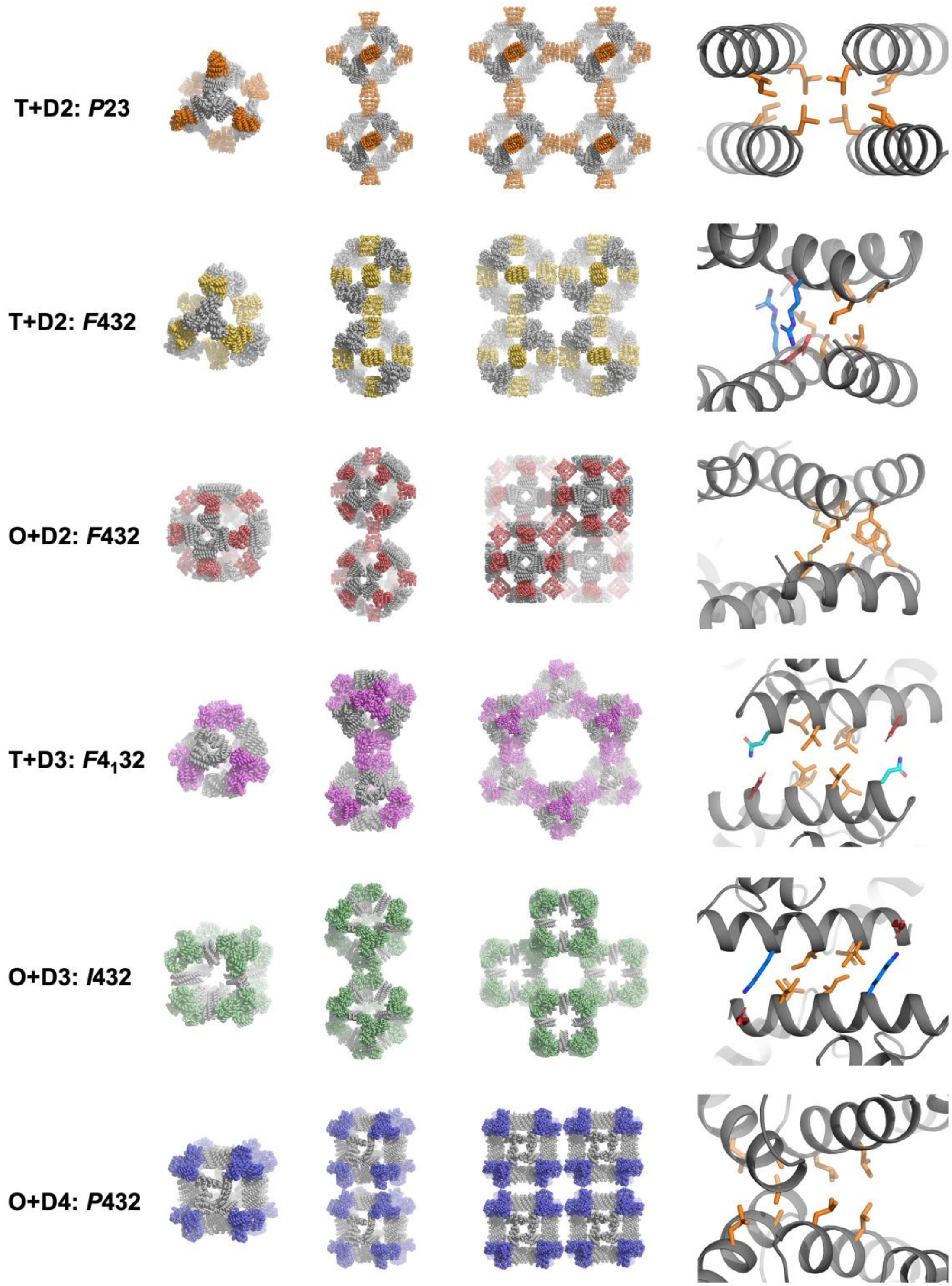

Supplementary Figure 1. *In-silico* 2-component 3D protein crystal designs by the hierarchical computational design approach. Combining tetrahedral/ octahedral symmetries with dihedral centers give rise to a series of lattices of cubic space groups. From left to right are the constituting cage building blocks, docked neighboring cages, representative fractions of the lattices and the atomic models of the designed crystal contact.

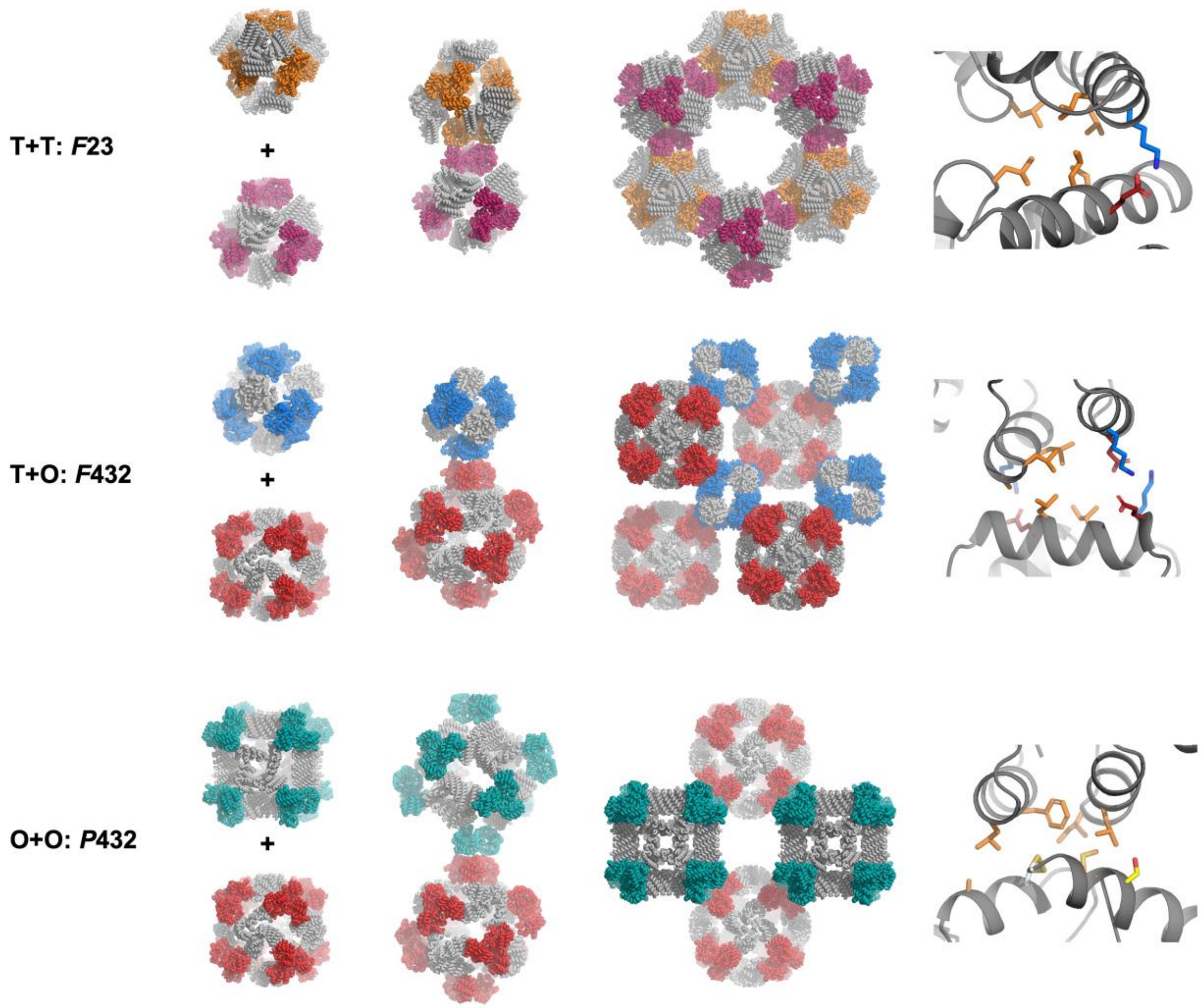

Supplementary Figure 2. *In-silico* 4-component 3D protein crystal designs by the hierarchical computational design approach. Combining tetrahedral and octahedral symmetries give rise to complex 4-component crystal designs. From left to right are the constituting cage building blocks, docked neighboring cages, representative fractions of the lattices and the atomic models of the designed crystal contact.

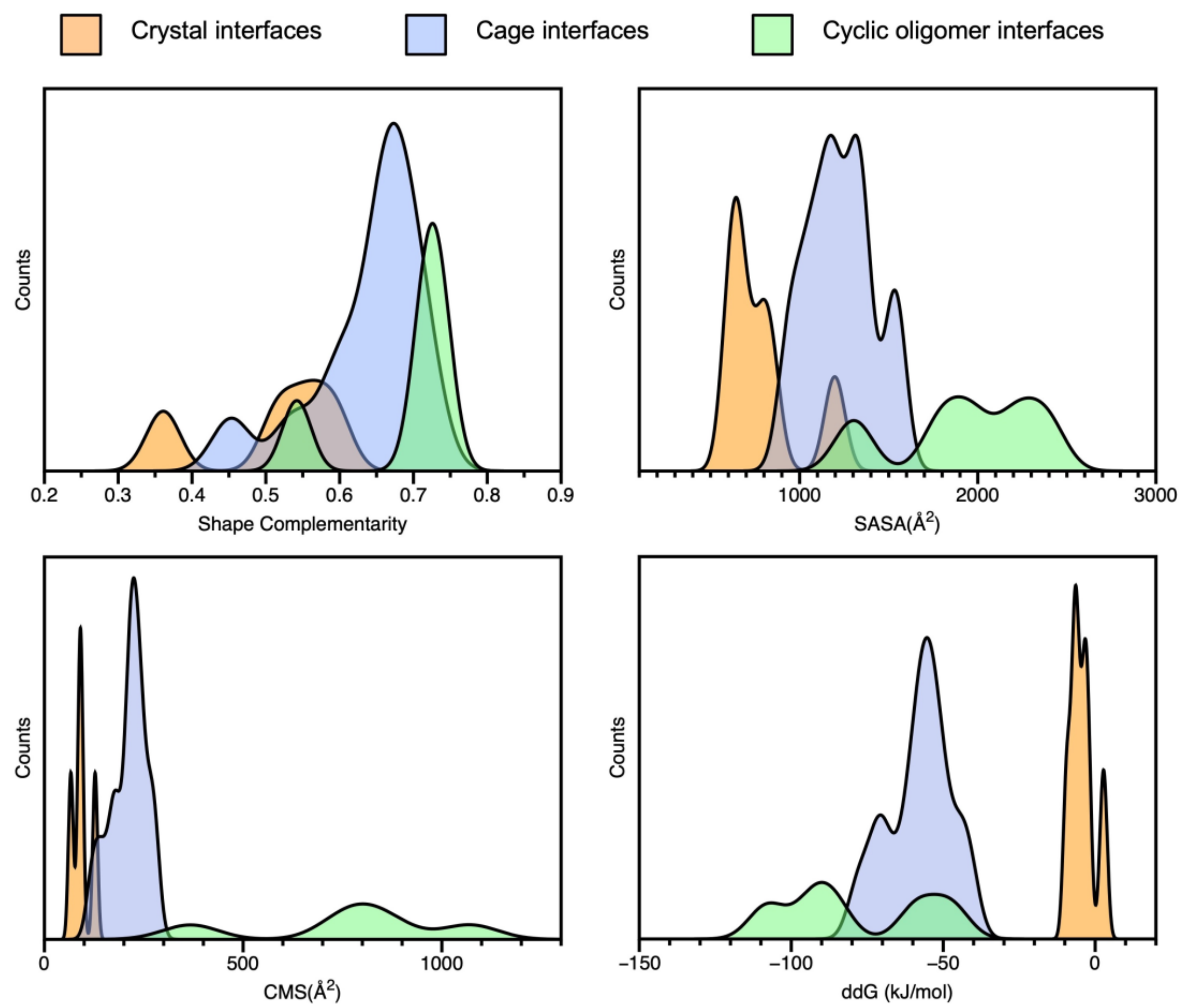

Supplementary Figure 3. Histograms of the Rosetta interface metrics for crystal, cage and cyclic oligomer interfaces. The crystal interfaces generally have lower shape complementarity, obviously smaller interface size (sasa < 1000 Å^2, cms < 100 Å^2) and higher interface free energy (>10 kcal/mol), indicating weaker and smaller interfaces than cage and cyclic oligomers.

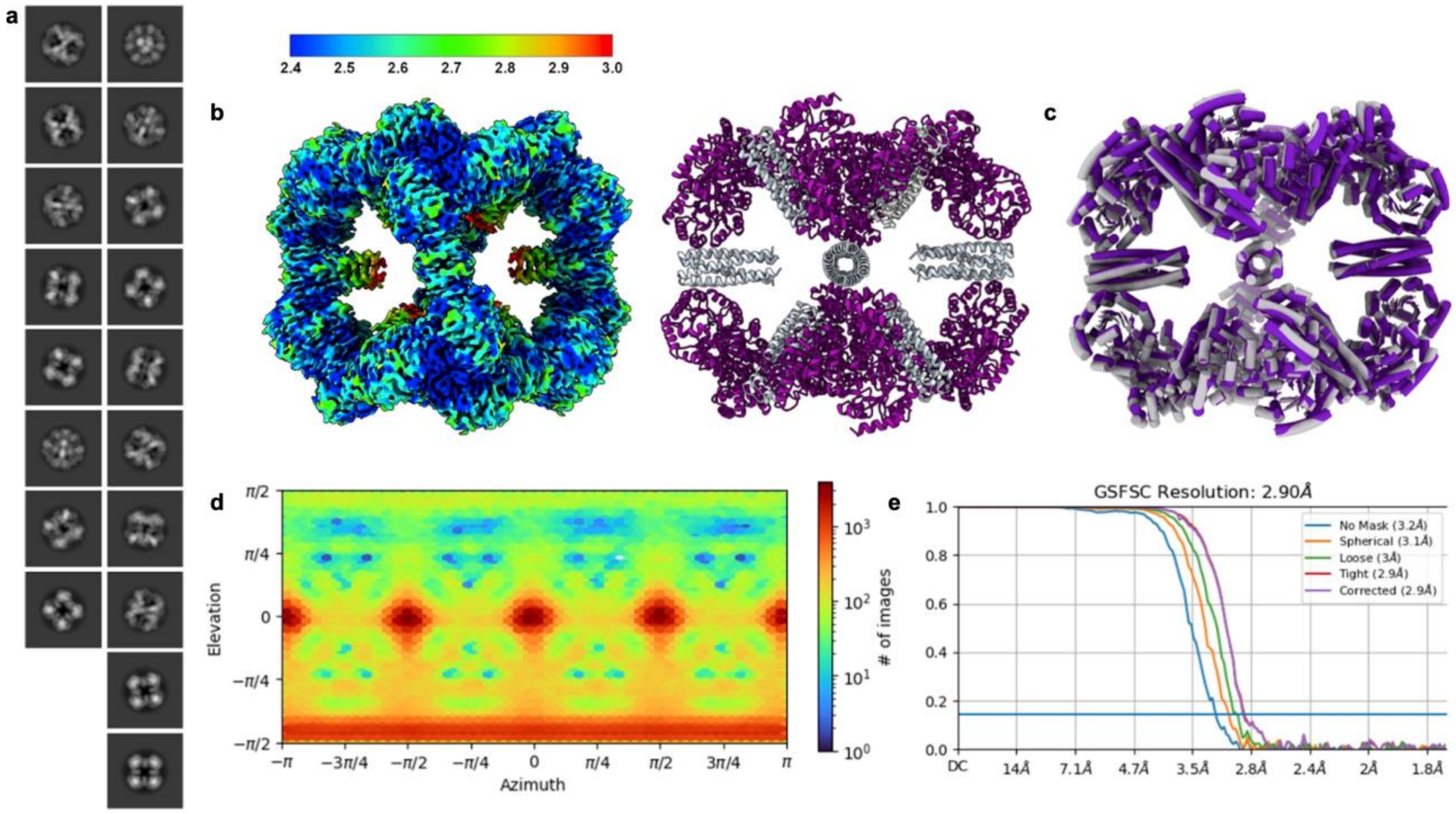

Supplementary Figure 4. CryoEM data of the O32-ZL4 cage. **a**, Representative 2D class averages. **b**, CryoEM local resolution map of (left) and built atomic model (right). **c**, Overlay of the design model with the cryoEM model. **d**, Orientational distribution plot demonstrating full angular sampling. **e**, Global FSC.

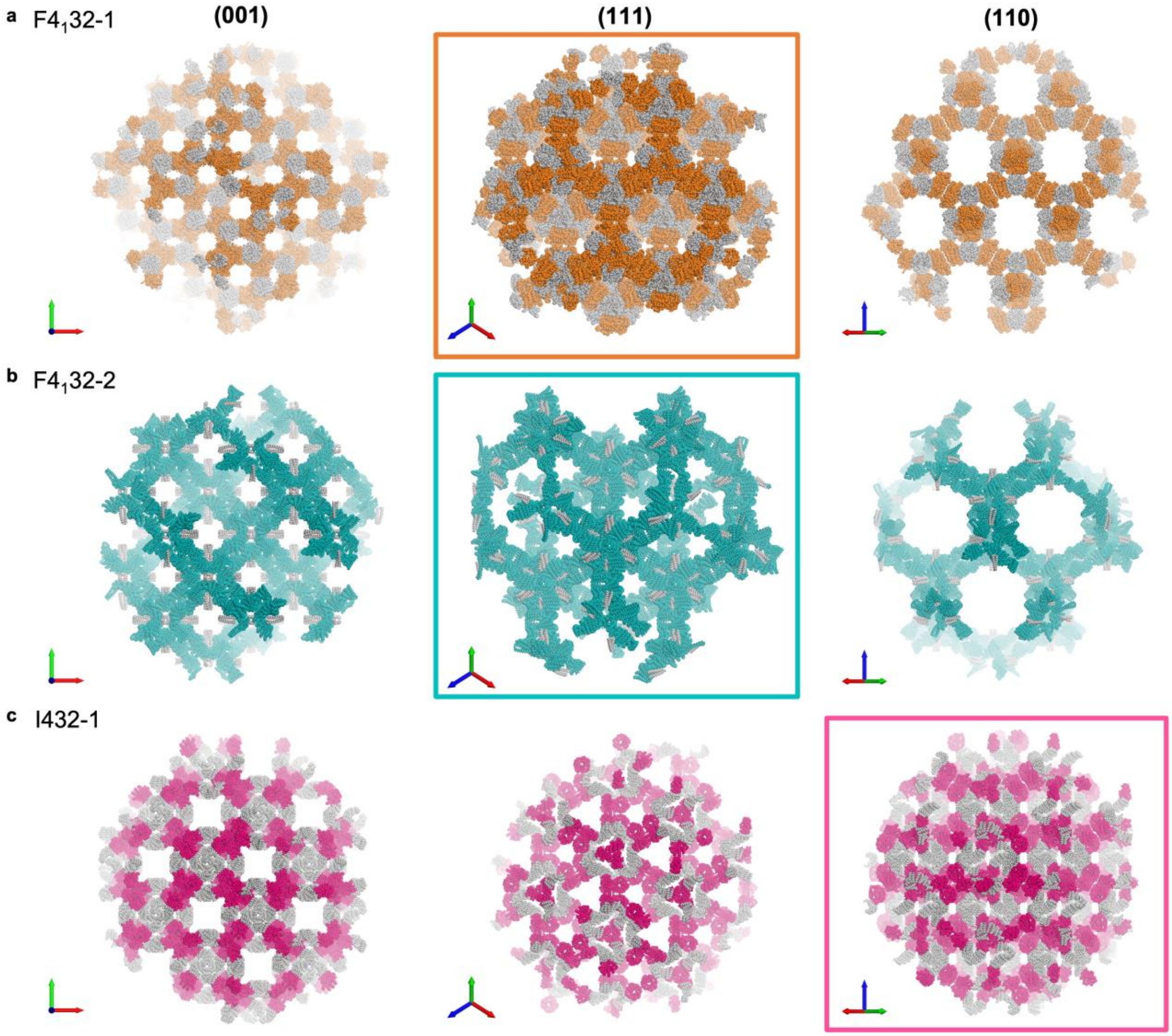

Supplementary Figure 5. Models of (001), (111) and (110) facets in designed crystals. The boxed facet has the highest packing density and the lowest surface energy. The relative energies of different crystal facets also determined the crystal habit in each case. When the nanocages assemble into a diamond lattice (as in $F4_132$-1 and $F4_132$-2 crystals), their (111) facet is the most densely packed plane of the lowest energy. When using the standard broken-bond model approximation for surface energy, exposing the closest-packed plane is thermodynamically favored: it requires breaking the smallest number of particle-to-particle interactions per unit area and thus exposes the lowest-surface-energy facet. Thus, basing on Wulff construction, the equilibrium shape of these crystals expose (111) facets, resulting in octahedral shape. Similarly, for *I*432-1 crystals, the crystals expose (110) facets of the lowest energy and grow into rhombic dodecahedral shapes.

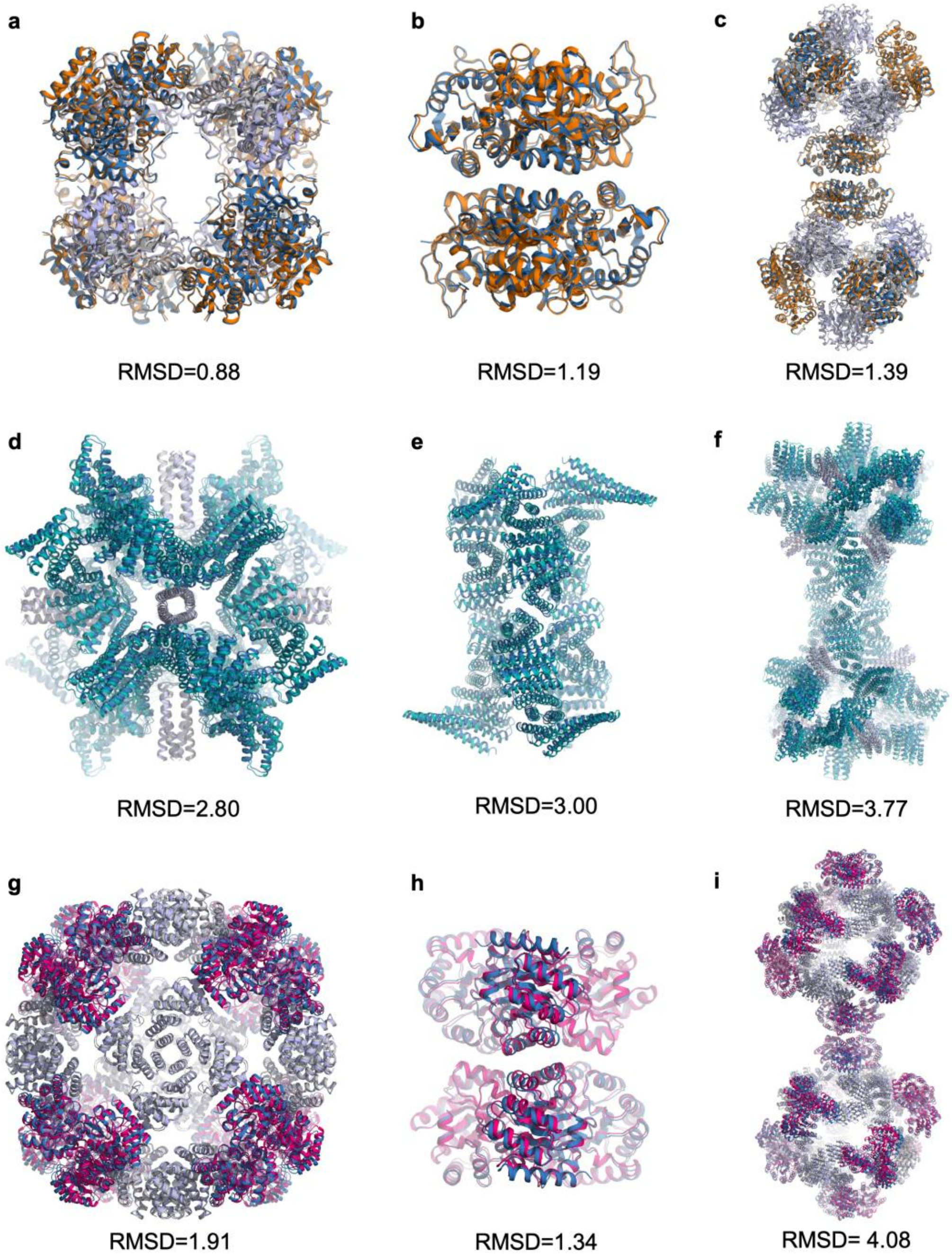

Supplementary Figure 6. The alignment of X-ray structure and computational design model (orange, teal, hot pink and gray) of designed protein crystals (sky blue and light blue). **a**, Alignment of T33 cages of $F4_132$-1 crystals. **b**, Alignment of D3 dihedrals of $F4_132$-1 crystals. **c**, Alignment of two neighboring cages of $F4_132$-1 crystals. **d**, Alignment of T32 cages of $F4_132$-2 crystals. **e**, Alignment of D3 dihedrals of $F4_132$-2 crystals. **f**, Alignment of two neighboring cages of $F4_132$-2 crystals. **g**, Alignment of O43 cages of *I*432-1 crystals. **h**, Alignment of D3 dihedrals of *I*432-1-CC crystals. **i**, Alignment of two neighboring cages of *I*432-1-CC crystals. All-atom RMSDs are shown below each alignment.

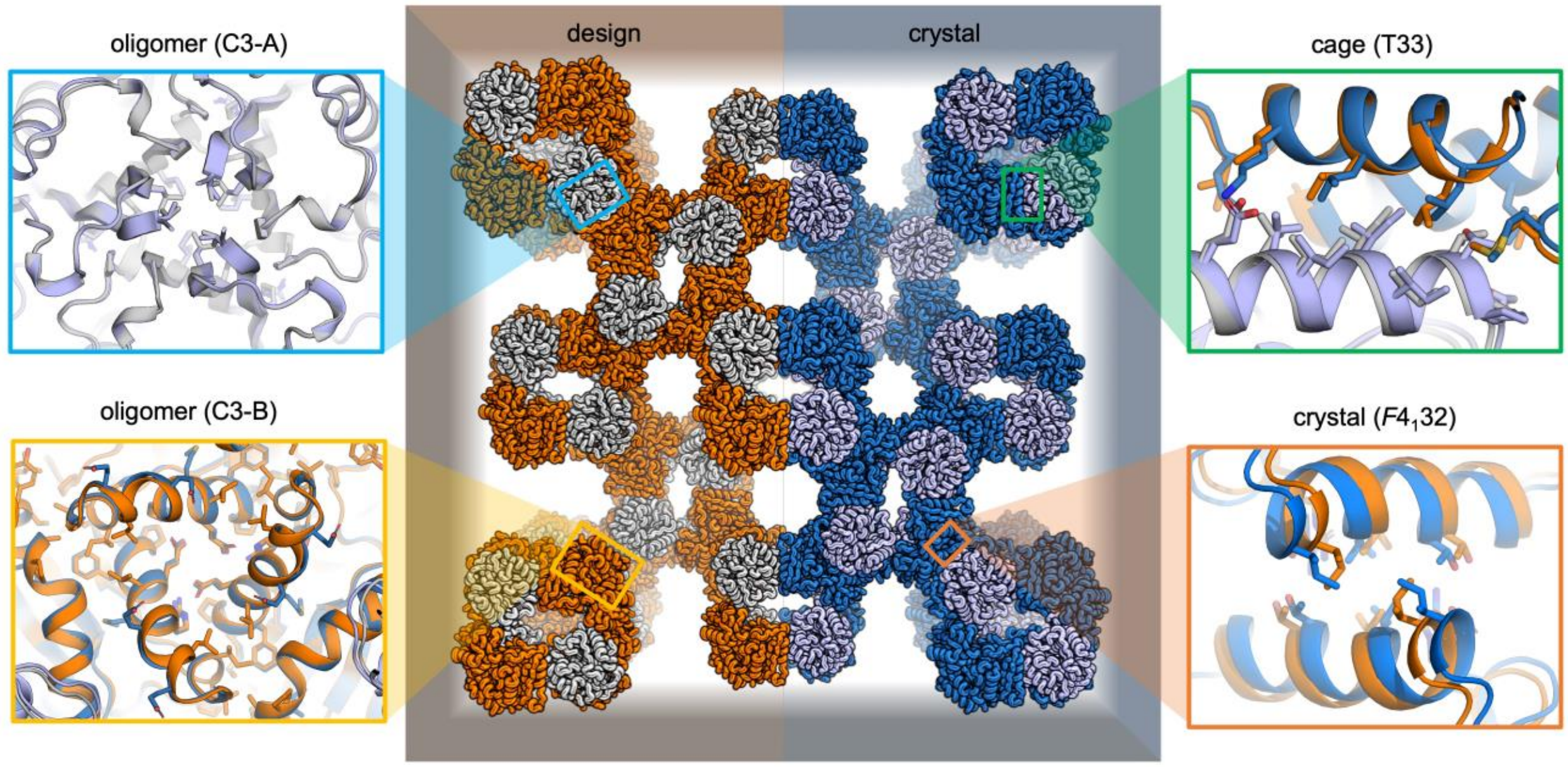

Supplementary Figure 7. X-ray crystal structure of *F*4$_1$32-1-0 crystal. Computational design model (rotation corrected (methods), orange and gray) spliced with X-ray crystallography model (skyblue and lightblue) for one unit cell of the crystal. On the corners are close-up views of the four interfaces present in the crystal, design superimposed with crystal model: oligomeric interface of the components on the left, cage interface at right top and crystal interface at right bottom. All-atom RMSD of models: C3-A: 0.69 Å; C3-B: 0.58 Å; T33: 1.06 Å; *F*4$_1$32: 1.81 Å.

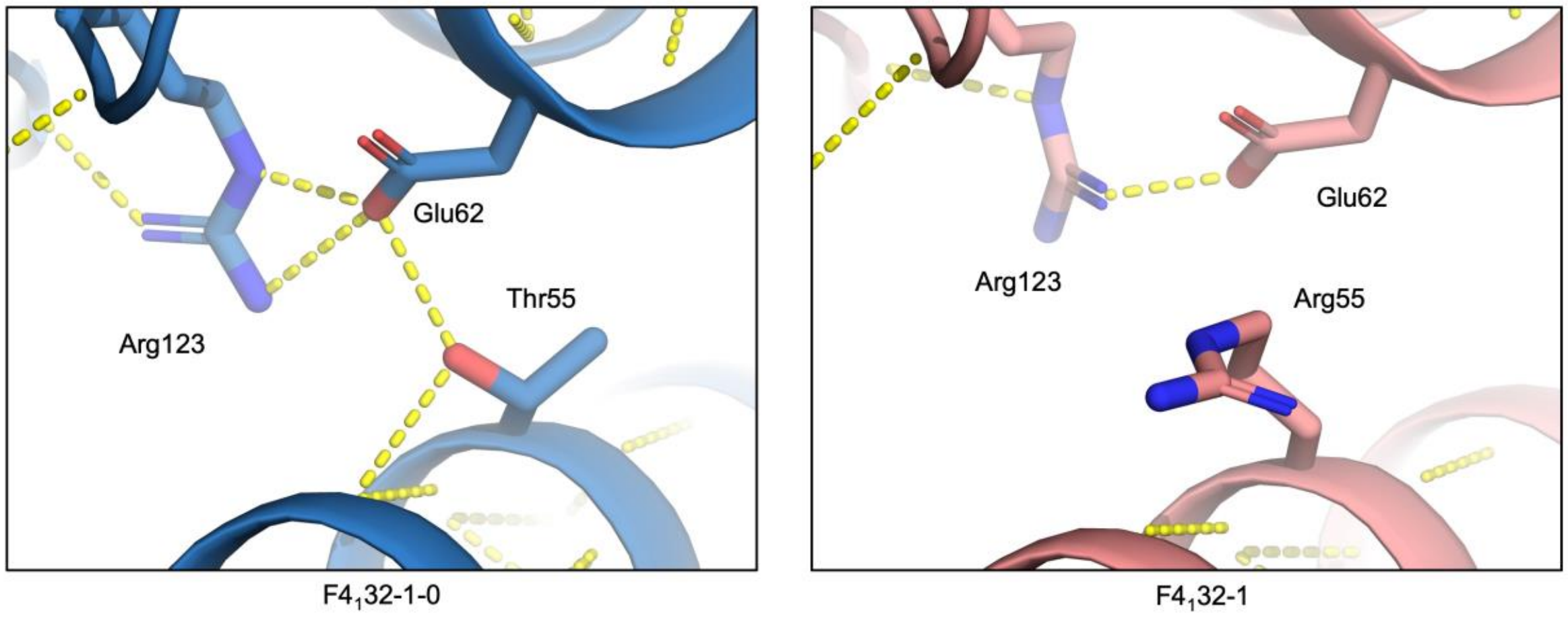

Supplementary Figure 8. A comparison of crystal structures of *F*4$_1$32-1-0 and *F*4$_1$32-1 around the T55R substitution. The observed shift in crystal contacts and deviation from the design model is attributed to an undesigned hydrogen bond network around Thr55.

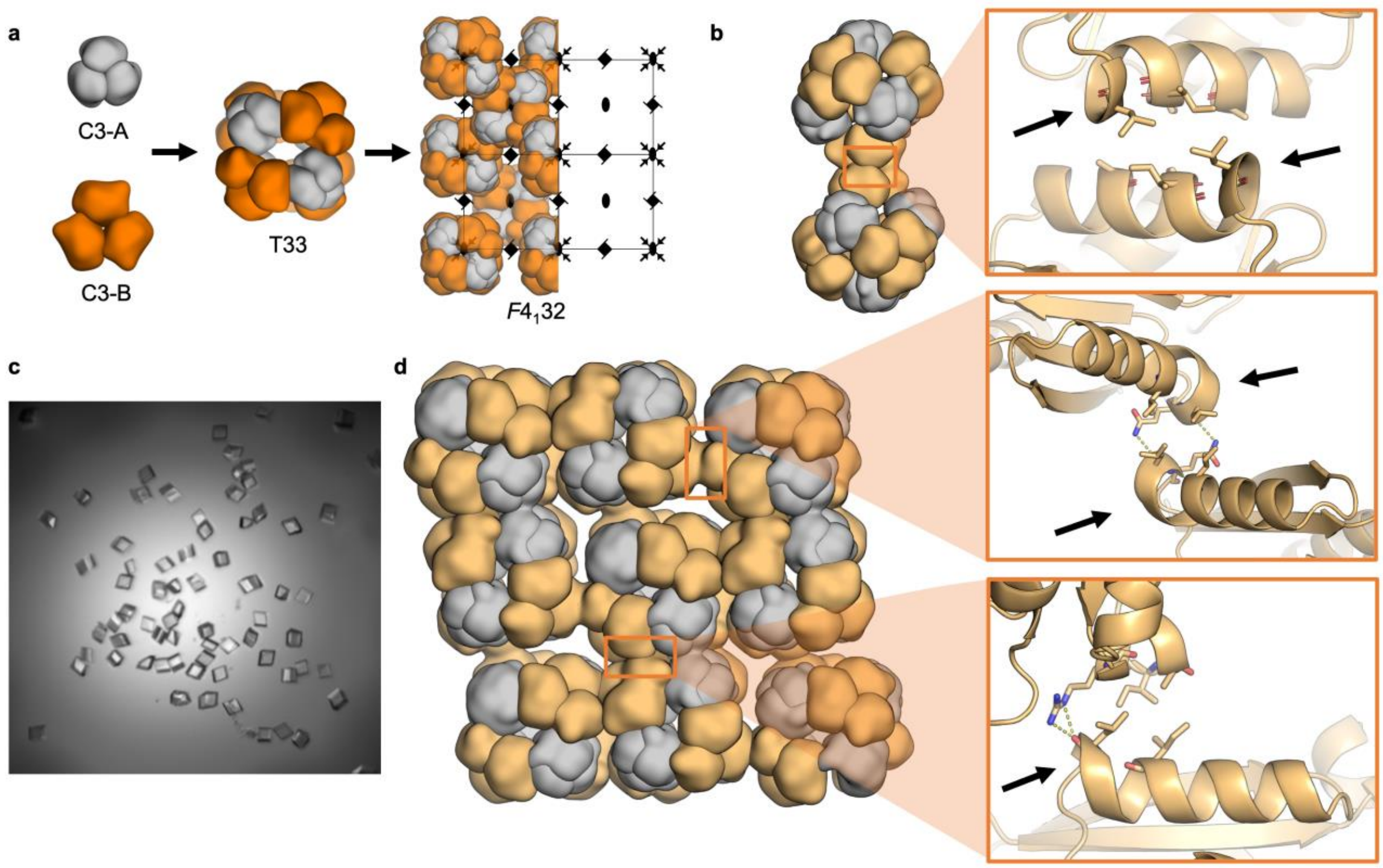

Supplementary Figure 9. Computational design and experimental characterization of *R*3-1. **a,** Overview of the hierarchical design. Symmetry elements of the cage are superimposed with corresponding symmetry elements of the $F4_132$ unit cell. **b,** Design model of adjacent docked T33-15-D3-6 cages and a close-up view of the designed crystal interface. **c,** Optical microscopy images of *R*3-1 crystals formed by mixing purified components at a 1:1 volumetric ratio in 300 mM NaCl, 25 mM Tris pH 8.0, 300 mM imidazole. **d,** Observed R3 crystal packing (light orange and gray) for one unit cell of the crystal. On the corners are close-up views of the two observed crystal contact interfaces present in the crystal. Black arrow points to a helical bulge present at both the designed crystal contact interface and the two observed off-target interfaces.

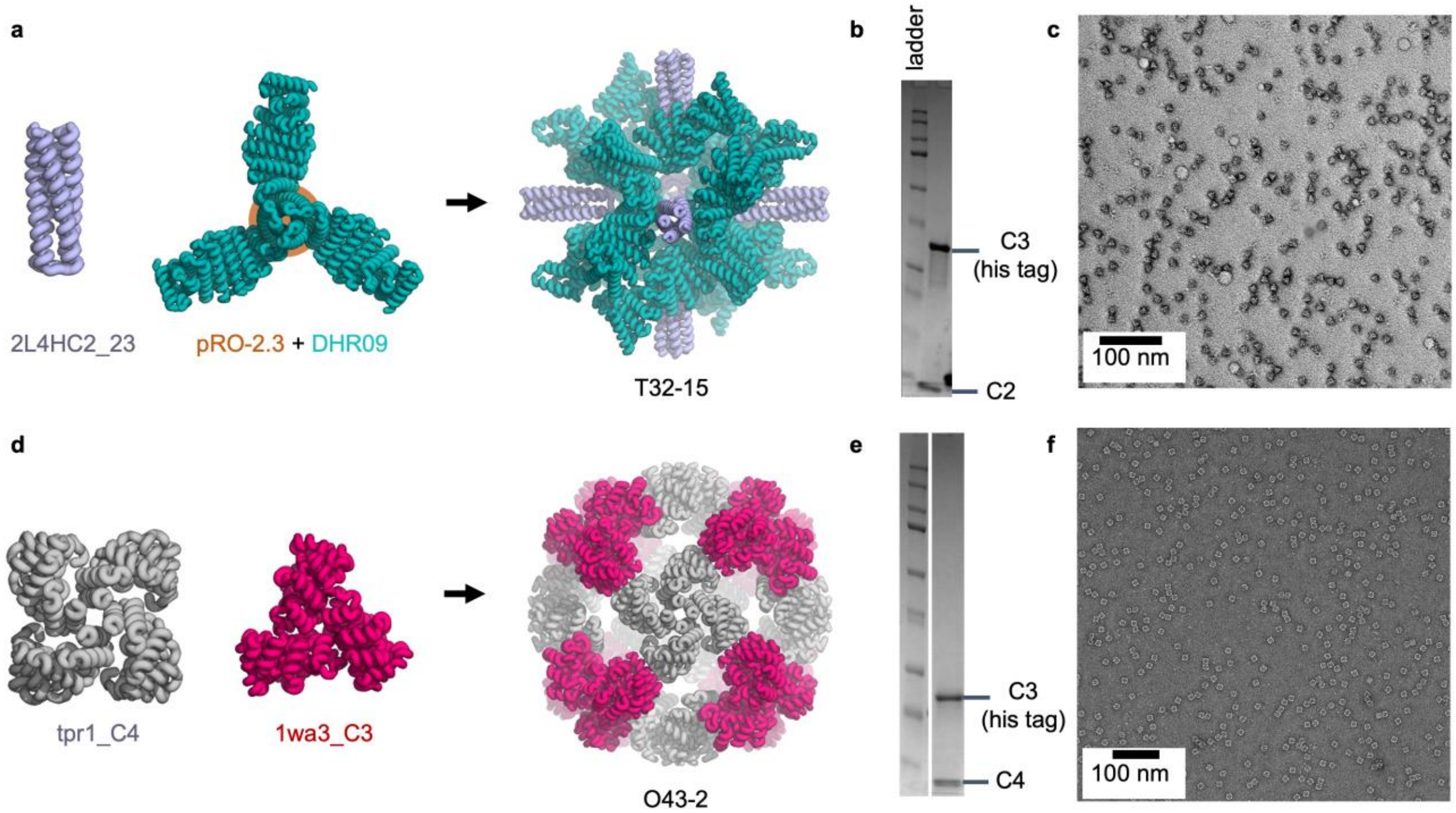

Supplementary Figure 10. Design scheme and experimental verification of the assembly of new cages. a, Design model and assembly scheme of T32-15 cage. **b**, SDS-PAGE of IMAC pull-down of protein co-expression of T32-15 cage. **c**, nsEM images of T32-15 cages from IMAC pull-down and SEC purification (assembled *in vivo*). **d**, Design model and assembly scheme of O43-2 cage. **e**, SDS-PAGE of IMAC pull-down of protein co-expression of O43-2 cage. **f**, nsEM images of O43 cages from IMAC pull-down and SEC purification (assembled *in vivo*).

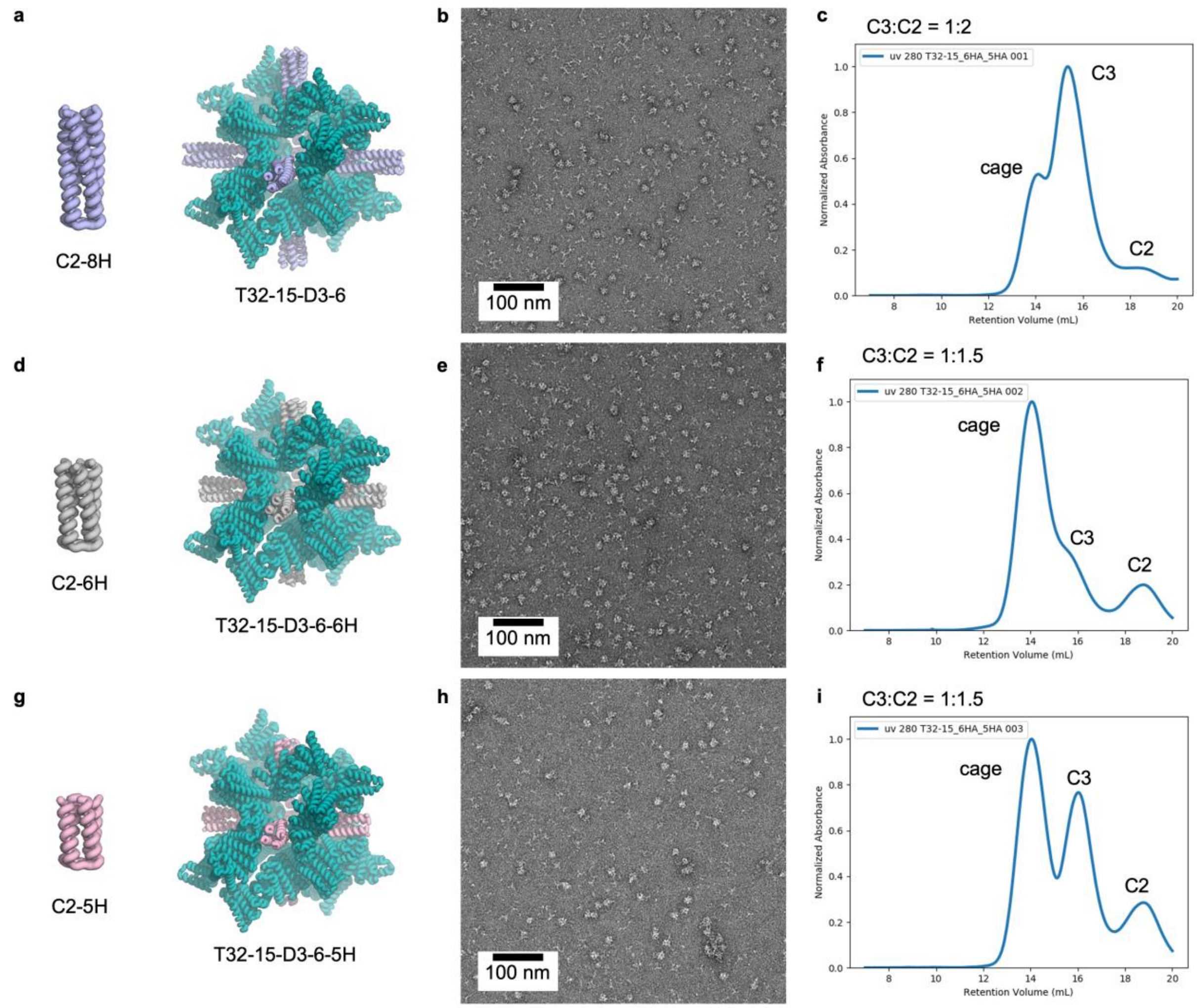

Supplementary Figure 11. T32-15-D3-6 cage with trimmed C2 component. a, The original T32-15-D3-6 design with C2 of 8 heptads (C2-8H), nsEM image of equivalent molar mixing and SEC traces. Cage assembly was not complete even with twice the equimolar amount of C2 components. **b,** T32-15-D3-6-6H design with C2 of 6 heptads (C2-6H), nsEM image of equivalent molar mixing and SEC traces. Cage assembly was almost complete with excess C2 components. **c,** T32-15-D3-6-5H design with C2 of 5 heptads (C2-5H), nsEM image of equivalent molar mixing and SEC traces. Incomplete cage assemblies were observed in the presence of excess C2 components.

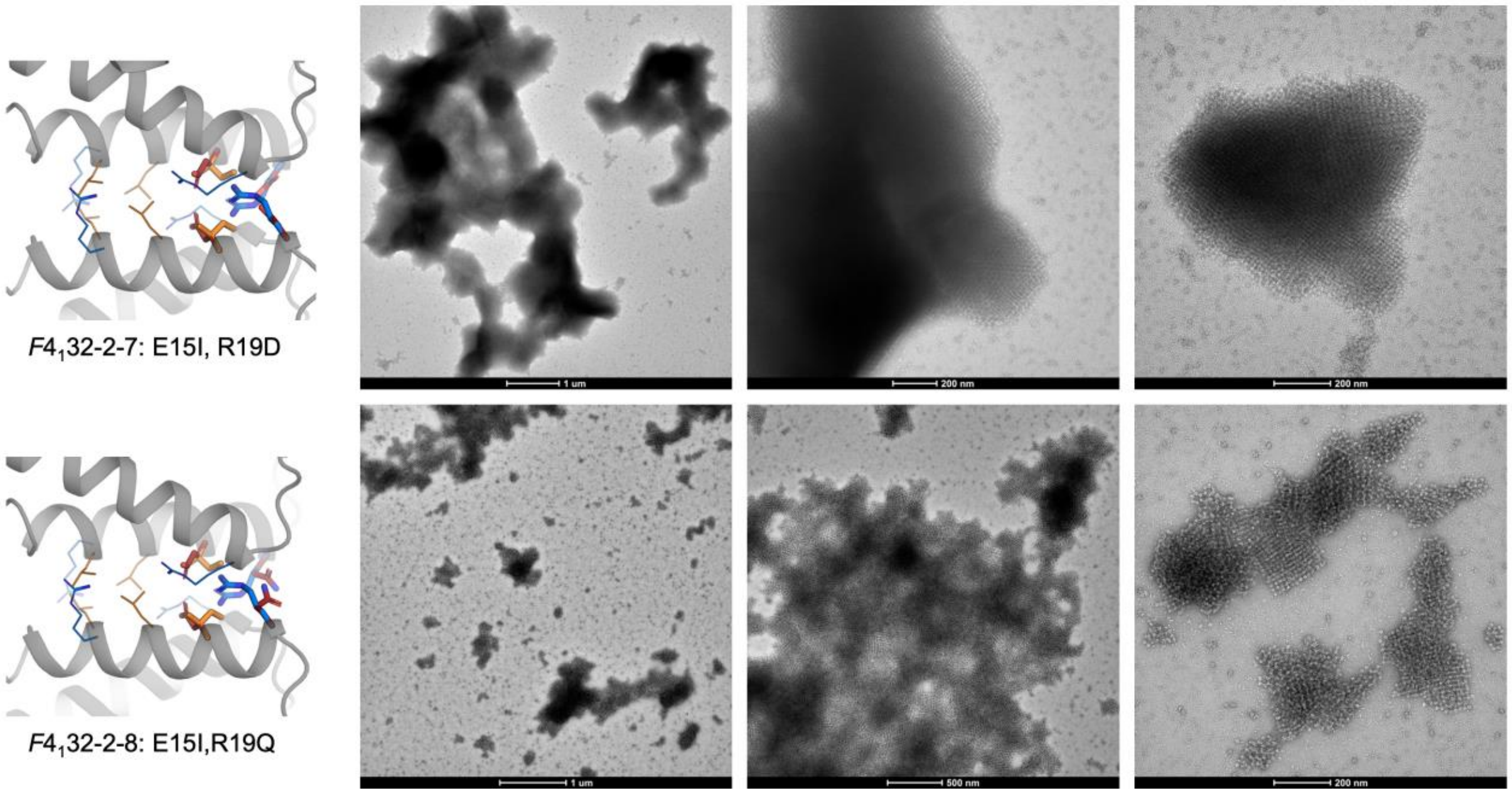

Supplementary Figure 12. Mutations of $F4_1$32-2 crystals into microcrystals and polycrystals. Models based on crystal structure (left) and their representative crystallization results characterized by nsEM (right).

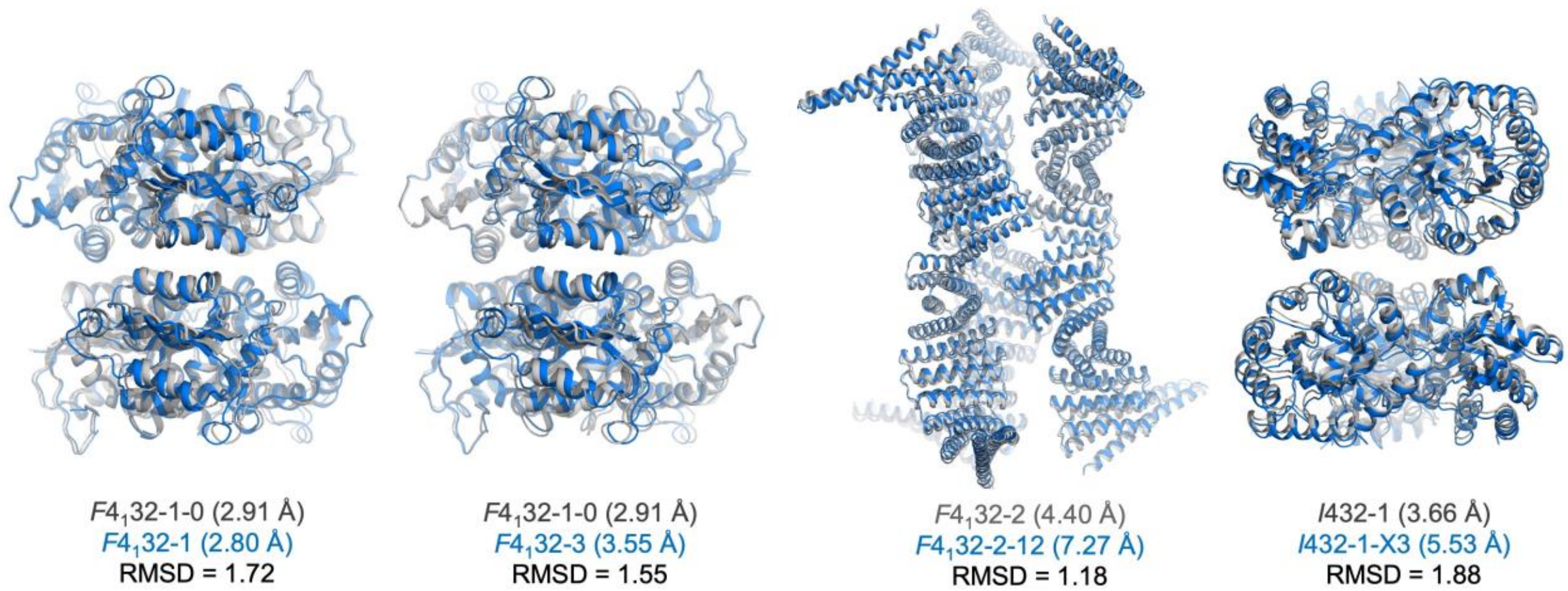

Supplementary Figure 13. X-ray structures of crystals with mutations as compared to their parent crystal aligned at the dihedral contacts. Small deviations and shifts of the backbones were observed, potentially due to the flexibility of the cages.

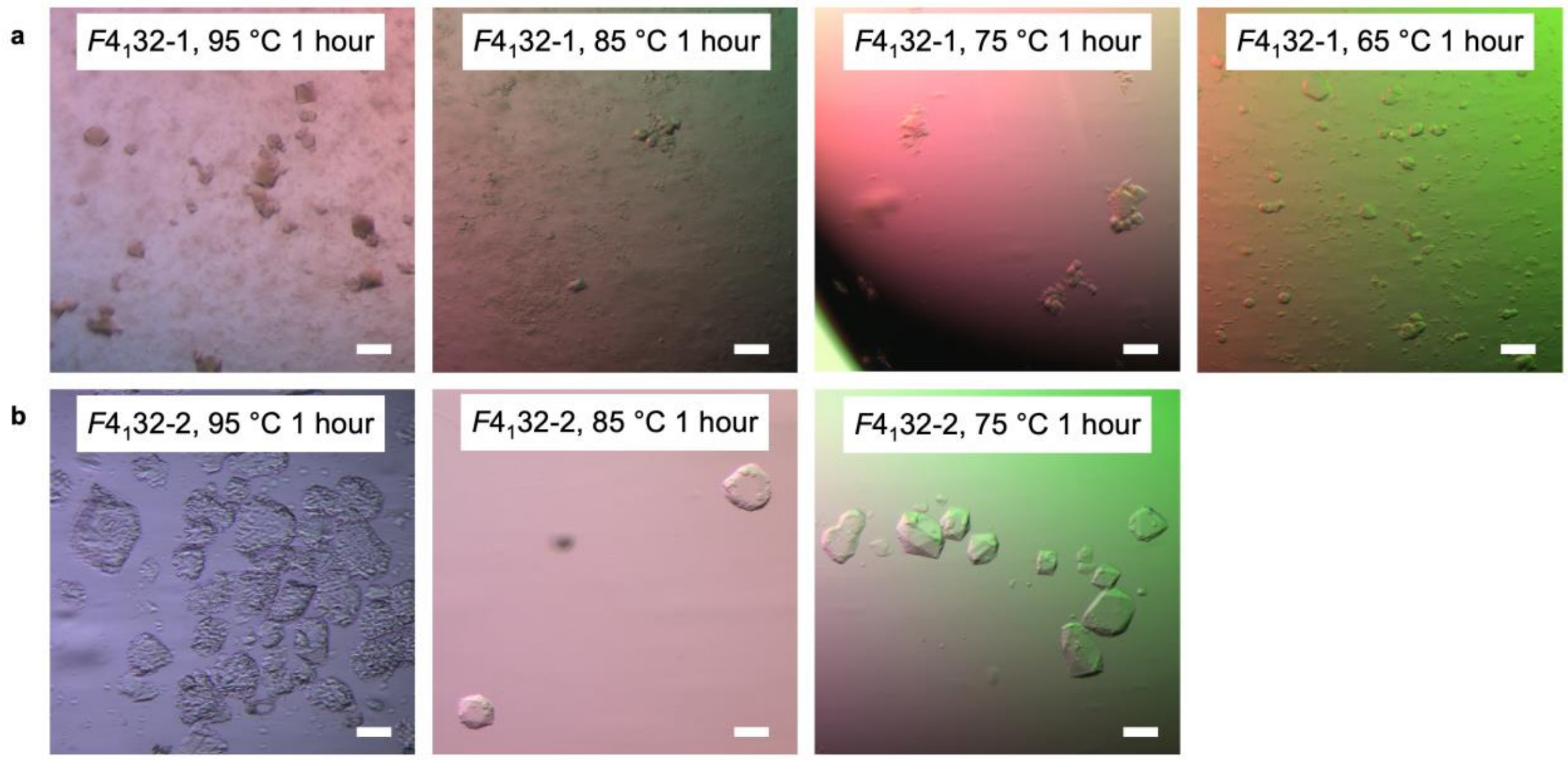

Supplementary Figure 14. Thermal stability of *F*4$_1$32-1 and *F*4$_1$32-2 crystals. a, Optical micrographs of *F*4$_1$32-1 crystals incubated at 95 °C, 85 °C, 75°C, and 65 °C for 1 hour. The crystals were stable up to 65 °C but started to clump together and the colloidal solution became turbid above 75°C. **b**, Optical micrographs of *F*4$_1$32-2 crystals incubated at 95 °C, 85 °C and 75°C for 1 hour. The crystals were stable below 85 °C while dissolved with visible pores at 95°C. See methods for experimental details. Scale bars, 100 µm.

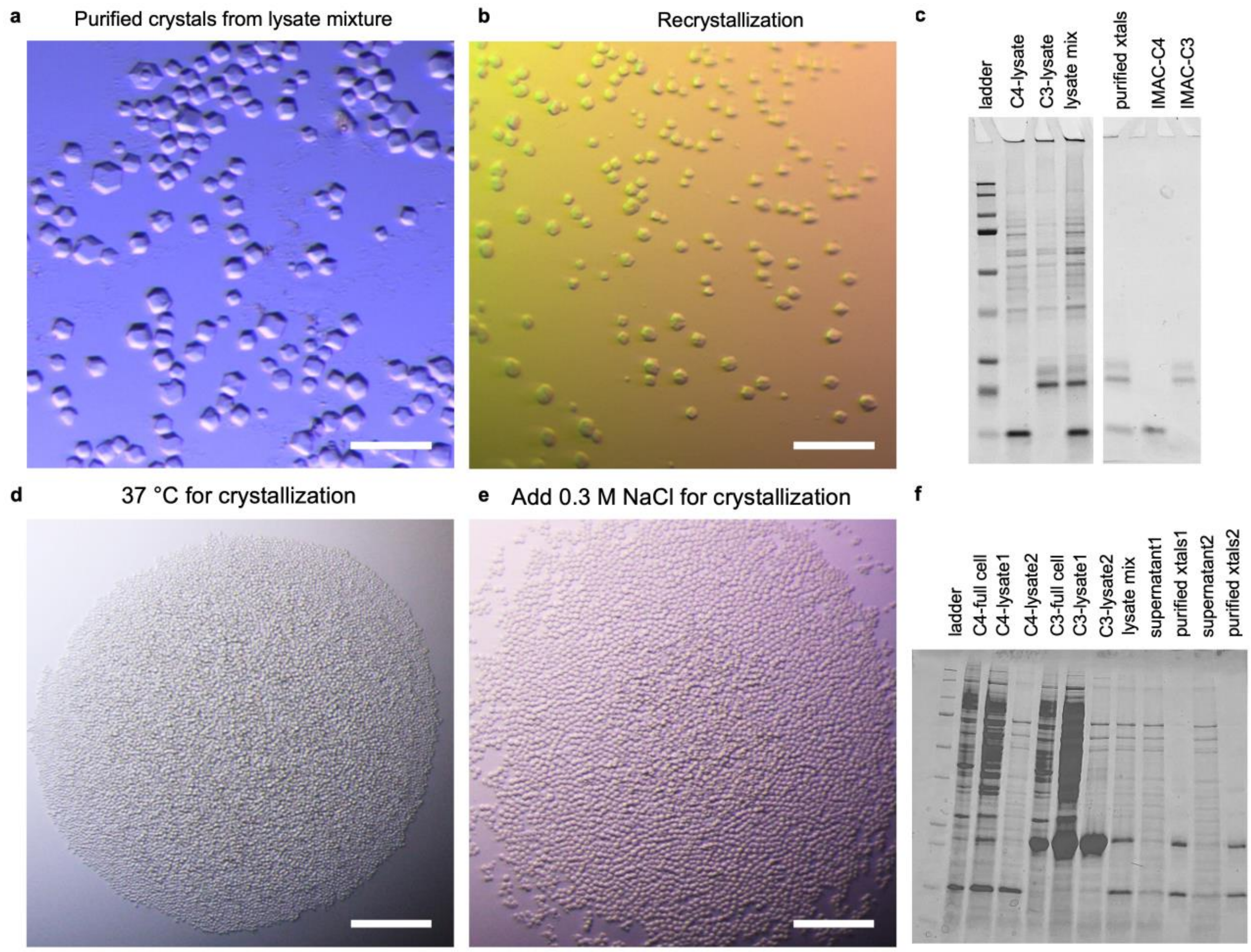

Supplementary Figure 15. Purification of *I*432-1-CC crystals from lysate mixture. a-c, Production from 50 mL of shake flask culture. **a,** Optical micrograph of crystals purified from mixing lysates overnight and centrifugation. **b**, Purified crystals dissolved in water and recrystallized in 0.5 M NaCl of hanging drop. **c**, SDS-PAGE of proteins from purified crystals. The protein yield is comparable to IMAC purification from the same amount of lysate. **d-f,** Production from 4L bioreactor culture. Cleared lysate solutions were boiled at 80 °C for 1 hour and cleared again by centrifugation. **d,** Optical micrograph of crystals from mixed lysates incubated at 37 °C overnight. **e,** Optical micrograph of crystals from mixed lysates incubated in 0.3 M NaCl overnight. **f**, SDS-PAGE of proteins from purified crystals. Lysate 1 and lysate 2 refer to proteins before and after the second clarification process by boiling. Supernatant 1 and crystals 1 are for samples in **d**. Supernatant 2 and crystals 2 are for samples in **e**. All scale bars, 100 μm.

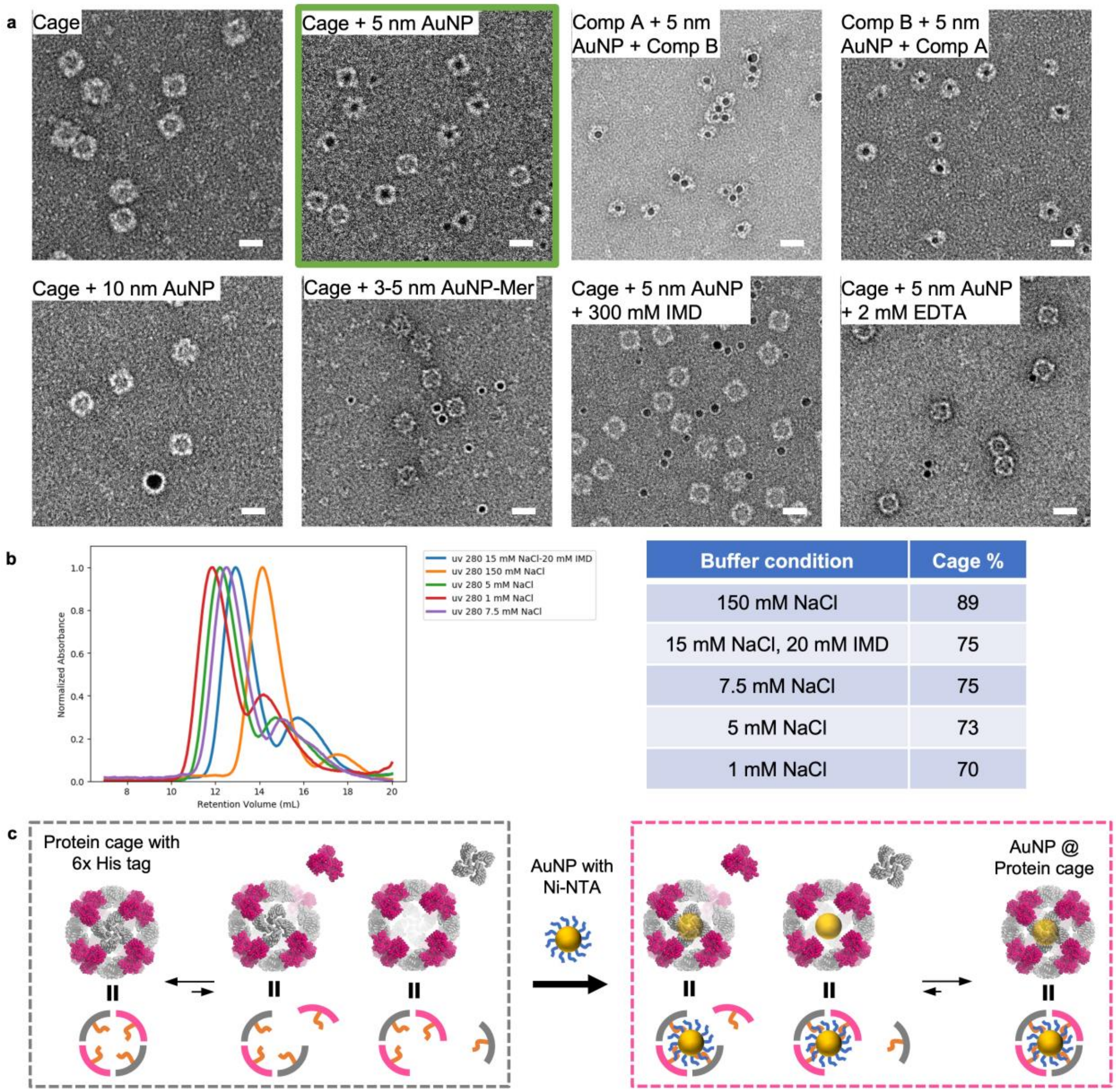

Buffer condition	Cage %
150 mM NaCl	89
15 mM NaCl, 20 mM IMD	75
7.5 mM NaCl	75
5 mM NaCl	73
1 mM NaCl	70

Supplementary Figure 16. Understanding the encapsulation of gold nanoparticles in protein nanocages. a, Control experiments for gold nanoparticle encapsulation in changing assembly order, gold nanoparticle size, surface modification and buffer conditions. **b,** Size exclusion chromatography for nanocage assembly under different low-salt conditions. The change of salt conditions slightly shifted the peak positions. The peaks of lower and higher retention volume in each trace are assigned as cage assembly and dissociated oligomers, and the area under the peaks were used to calculate the percentage of fully assembled cages as shown in the table on the right. **c,** Proposed encapsulation mechanism: gold nanoparticles are encapsulated during the nanocage assembly-disassembly equilibrium, driven by the coordination between inward-facing His tags of nanocages and the NTA ligands on nanoparticles. IMD, imidazole. EDTA, ethylenediaminetetraacetic acid. 5 nm and 10 nm AuNPs are modified with Ni-NTA. AuNP-Mer are 3-5 nm gold nanoparticles modified with (1-mercaptoundec-11-yl) tetraethylene glycol. All AuNPs are purchased from Nanoprobes. Scale bars, 20 nm.

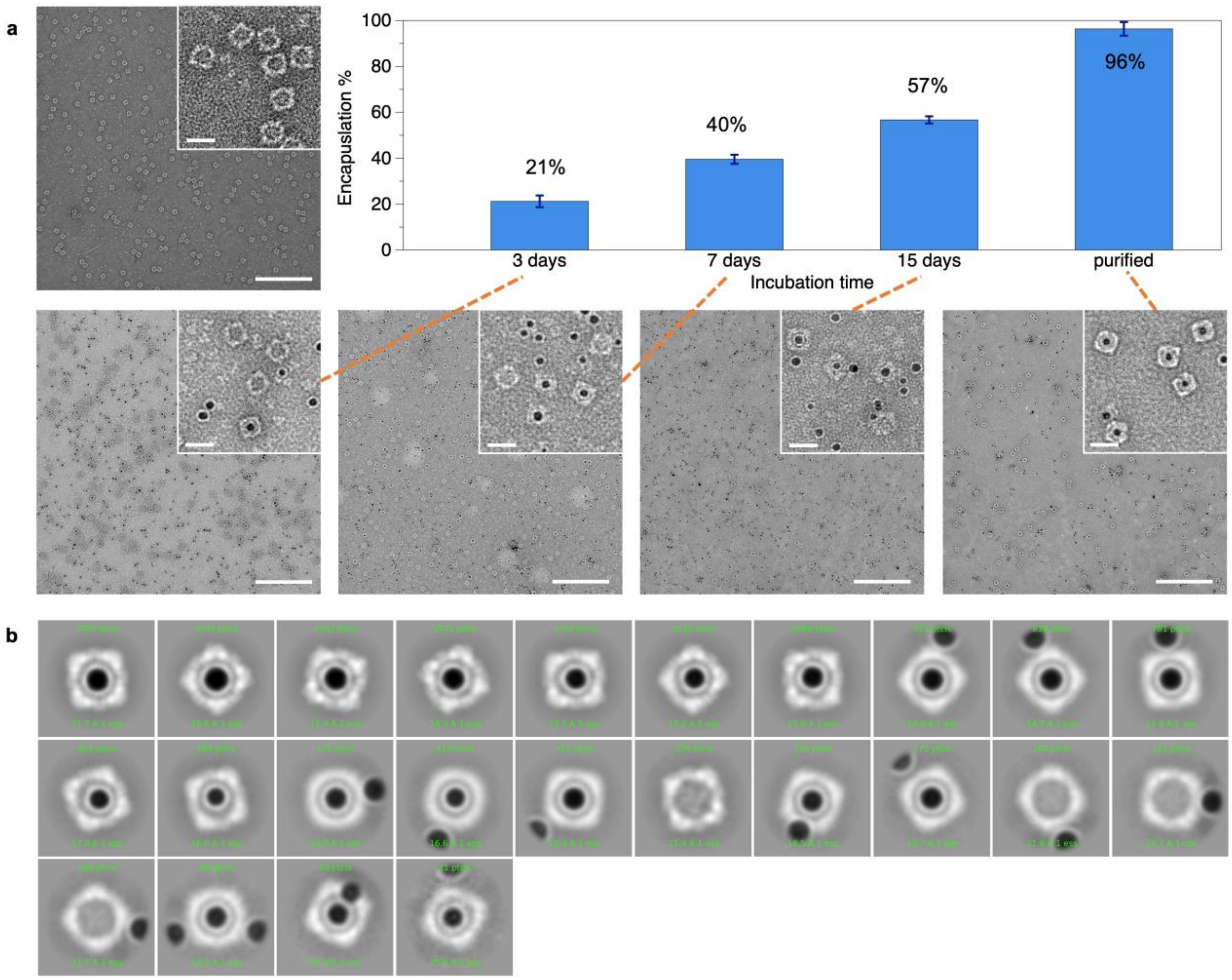

Supplementary Figure 17. Encapsulation of AuNPs in *I*432-1-CC octahedral cage. a, The yield of AuNP encapsulation over 15 days of incubation at room temperature. Yield for each sample was estimated by counting the number of empty and encapsulated cages (n = 400-800) in 3-5 nsEM images taken over the grid. Scale bar, 200 nm. Inset, scale bar, 20 nm. **b**, 2D class averages of the nsEM of purified AuNP@*I*432-1-CC cages by CryoSparc.

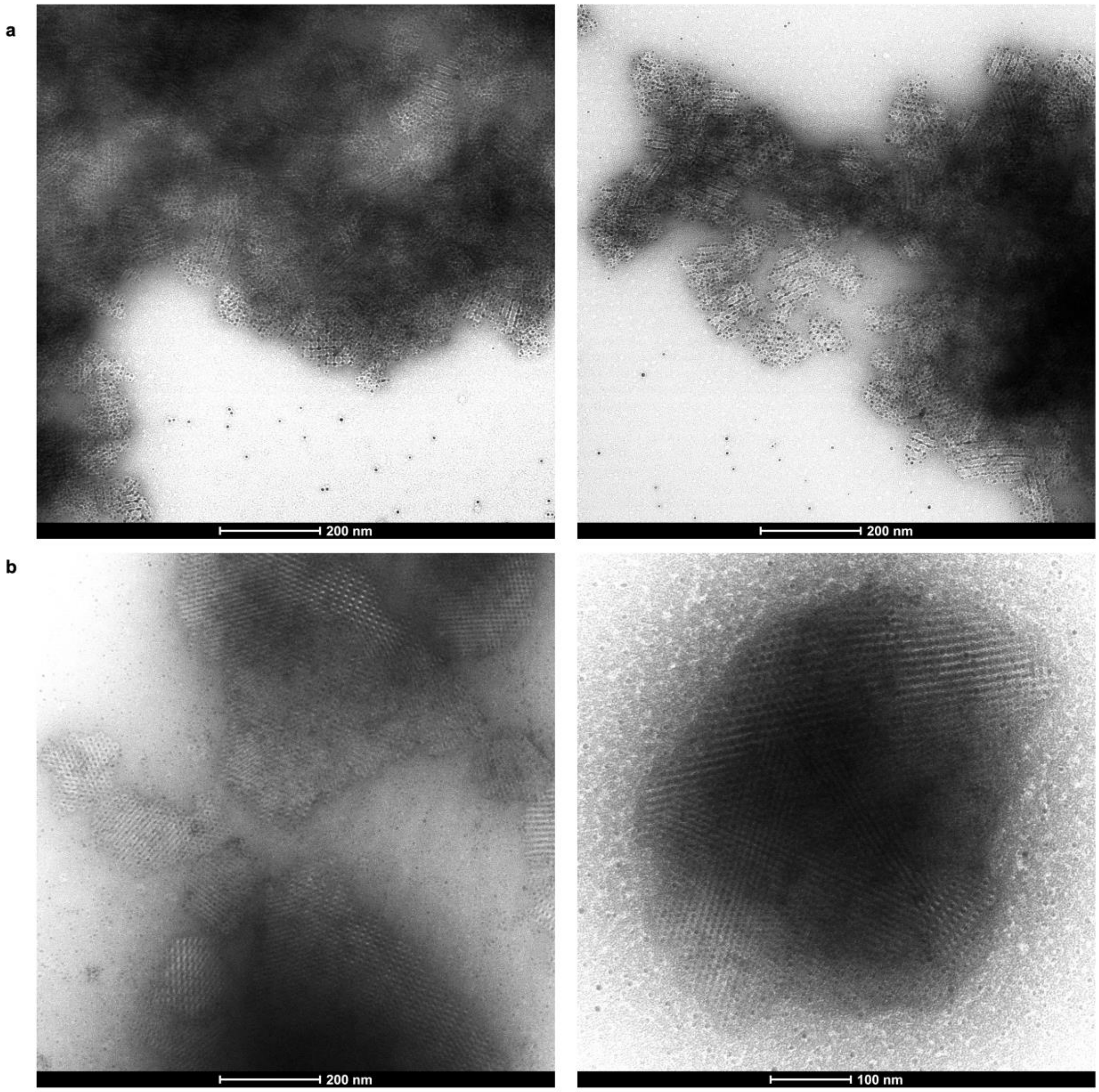

Supplementary Figure 18. nsEM micrographs of O43-2-D3-6 cage-AuNP coassembly (a) and crushed AuNPs@*I*432-1-CC crystals (b).

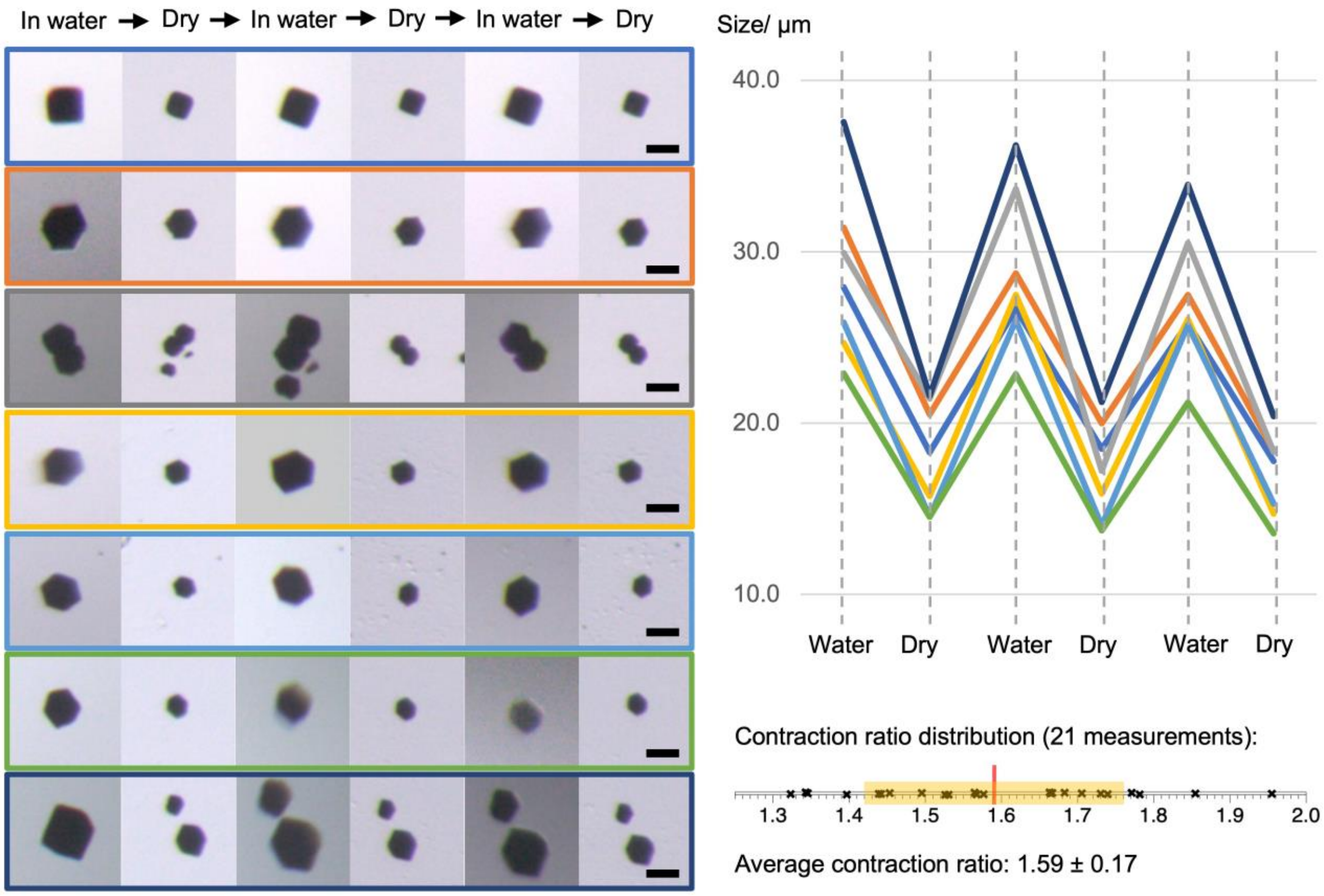

Supplementary Figure 19. Drying and rehydration cycles of crosslinked crystals in water with 100% AuNP encapsulation. During three cycles, different crystals were tracked for their size (measured as the diagonal distance) by the optical micrographs on the left. For all the contractions measured, the crystals contracted 1.59 ± 0.17 times on average. Scale bars, 20 μm.

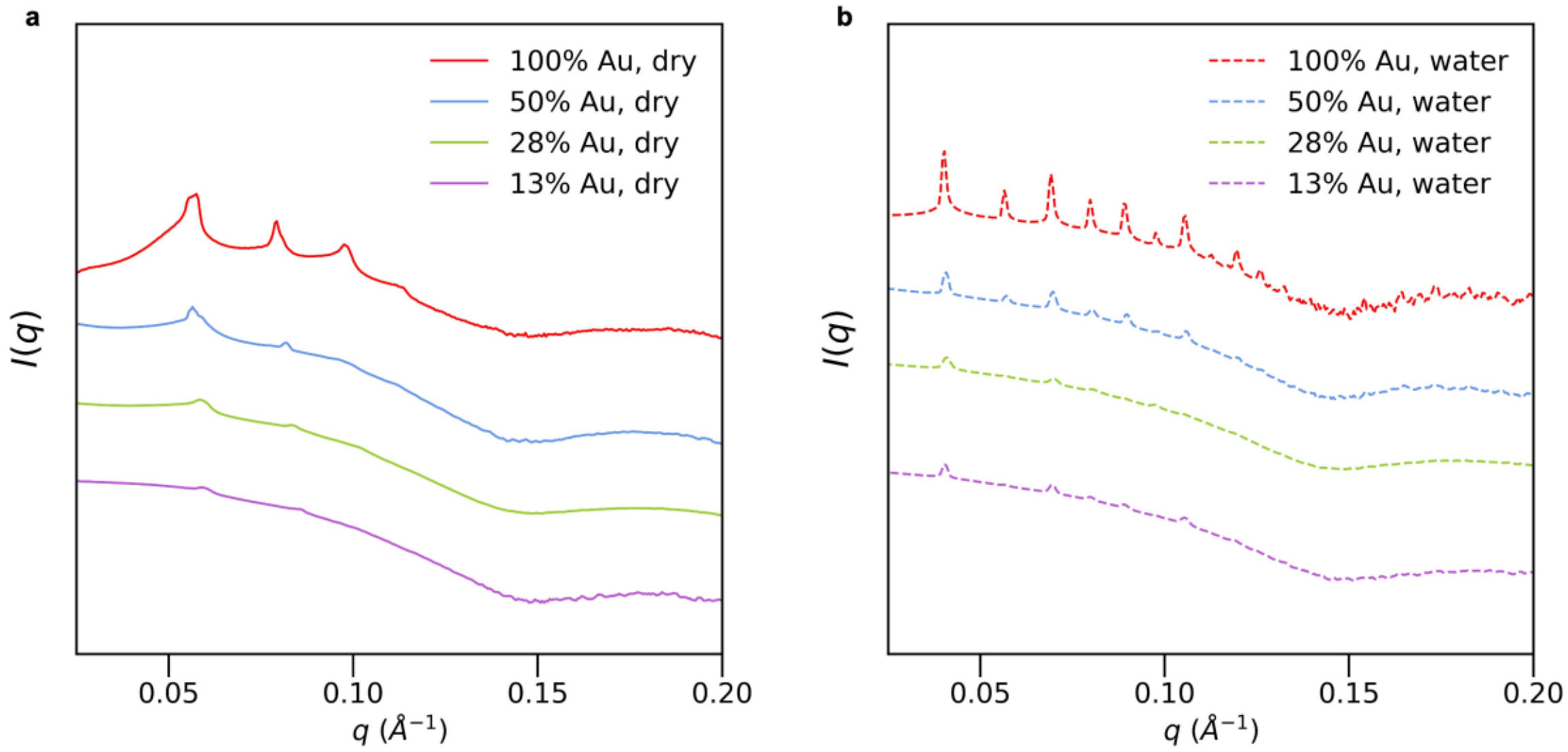

Supplementary Figure 20. SAXS profiles of AuNP encapsulated crystals in dry (a) and hydrated states (b), with different encapsulation ratios of AuNPs. Dried crystals have broader, fewer peaks shifted to higher angle, illustrating smaller and more deviated lattice spacing. Crystals with lower AuNP encapsulations show fewer peaks, possibly due to fewer scattering centers located at the lattice distance.

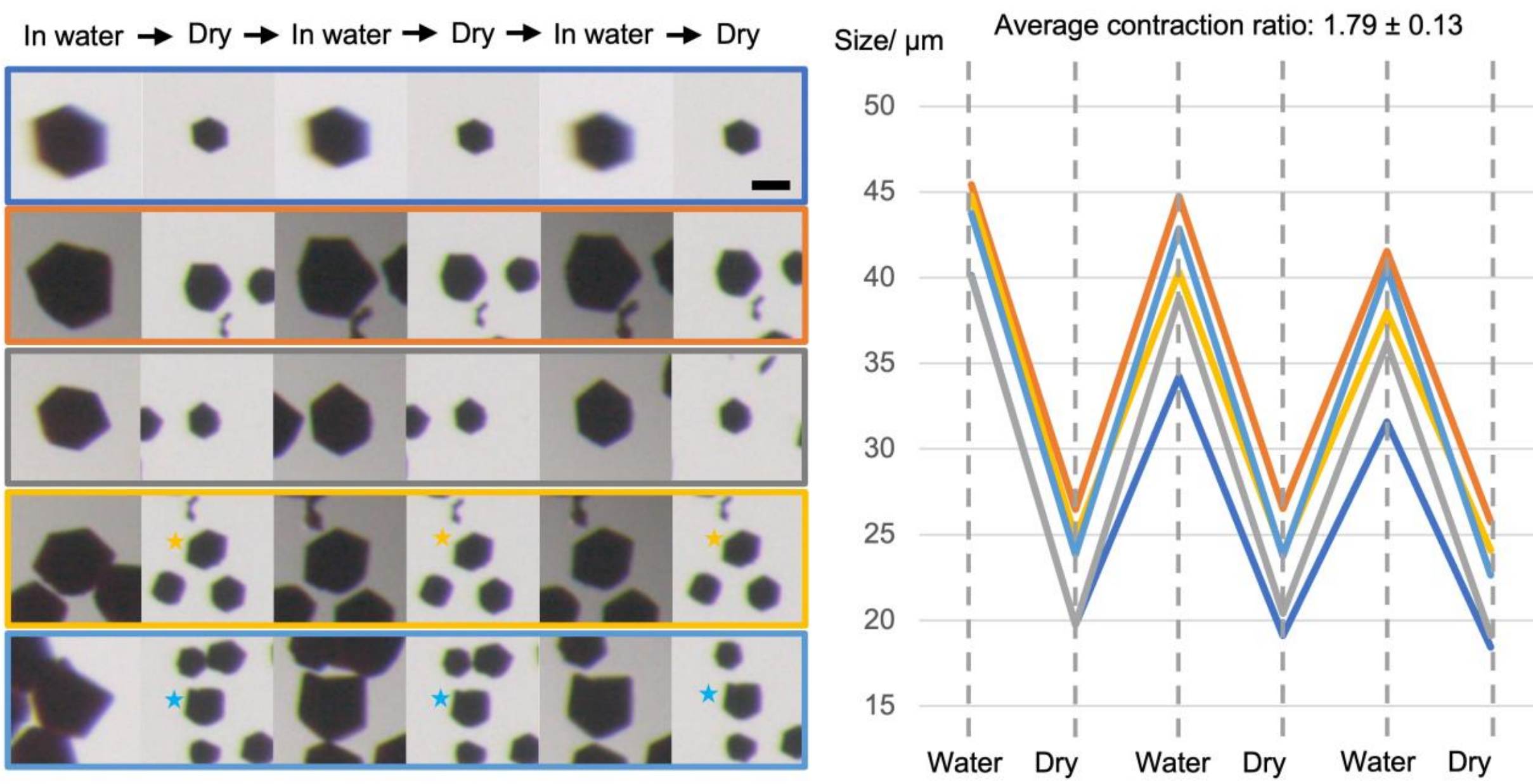

Supplementary Figure 21. Drying and rehydration cycles of crosslinked crystals in water with 50% AuNP encapsulation. During three cycles, different crystals were tracked for their size (measured as the diagonal distance) by the optical micrographs on the left. For all the contractions measured, the crystals contracted 1.79 ± 0.13 times on average. Scale bars, 20 µm.

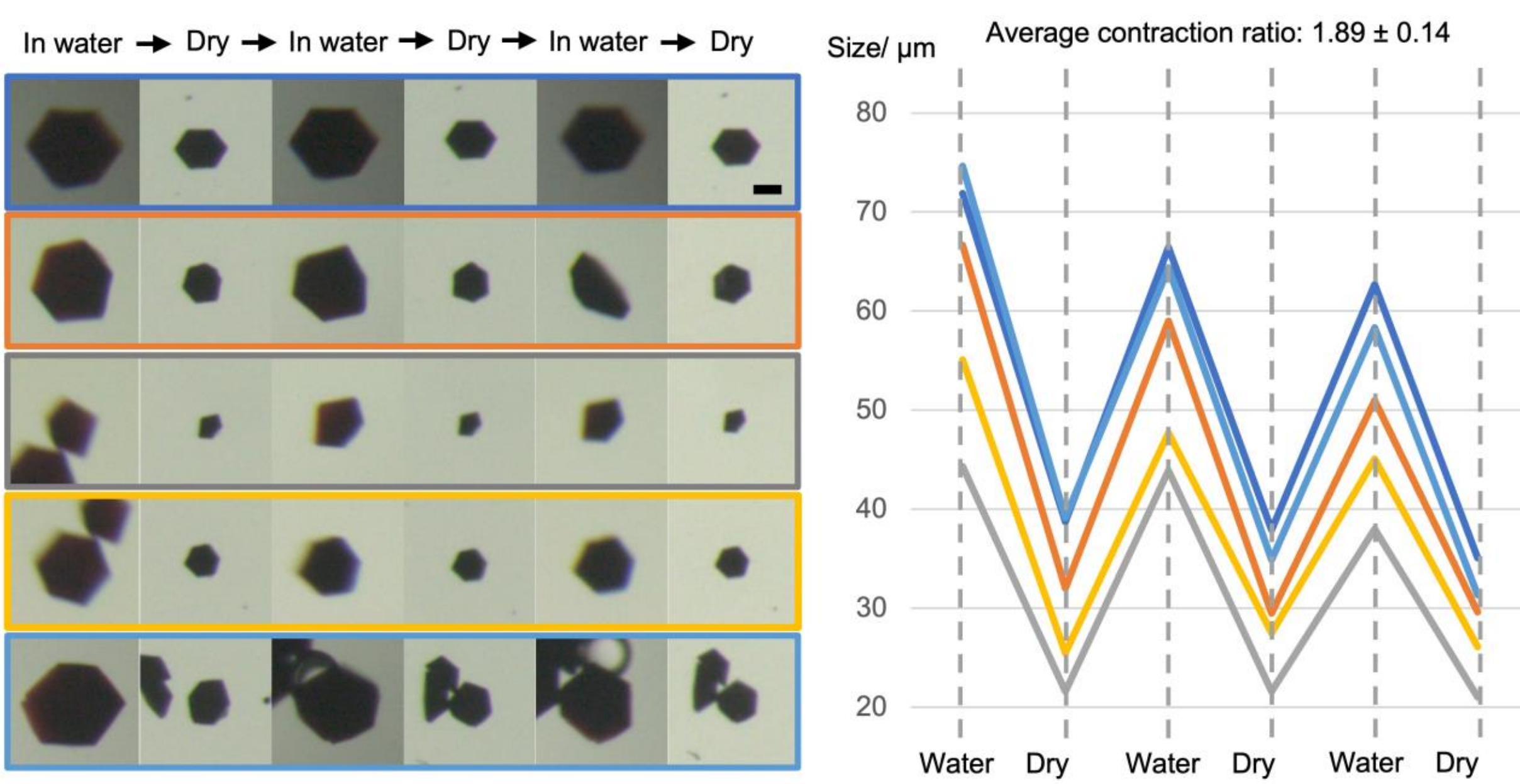

Supplementary Figure 22. Drying and rehydration cycles of crosslinked crystals in water with 28% AuNP encapsulation. During three cycles, different crystals were tracked for their size (measured as the diagonal distance) by the optical micrographs on the left. For all the contractions measured, the crystals contracted 1.89 ± 0.14 times on average. Scale bars, 20 µm.

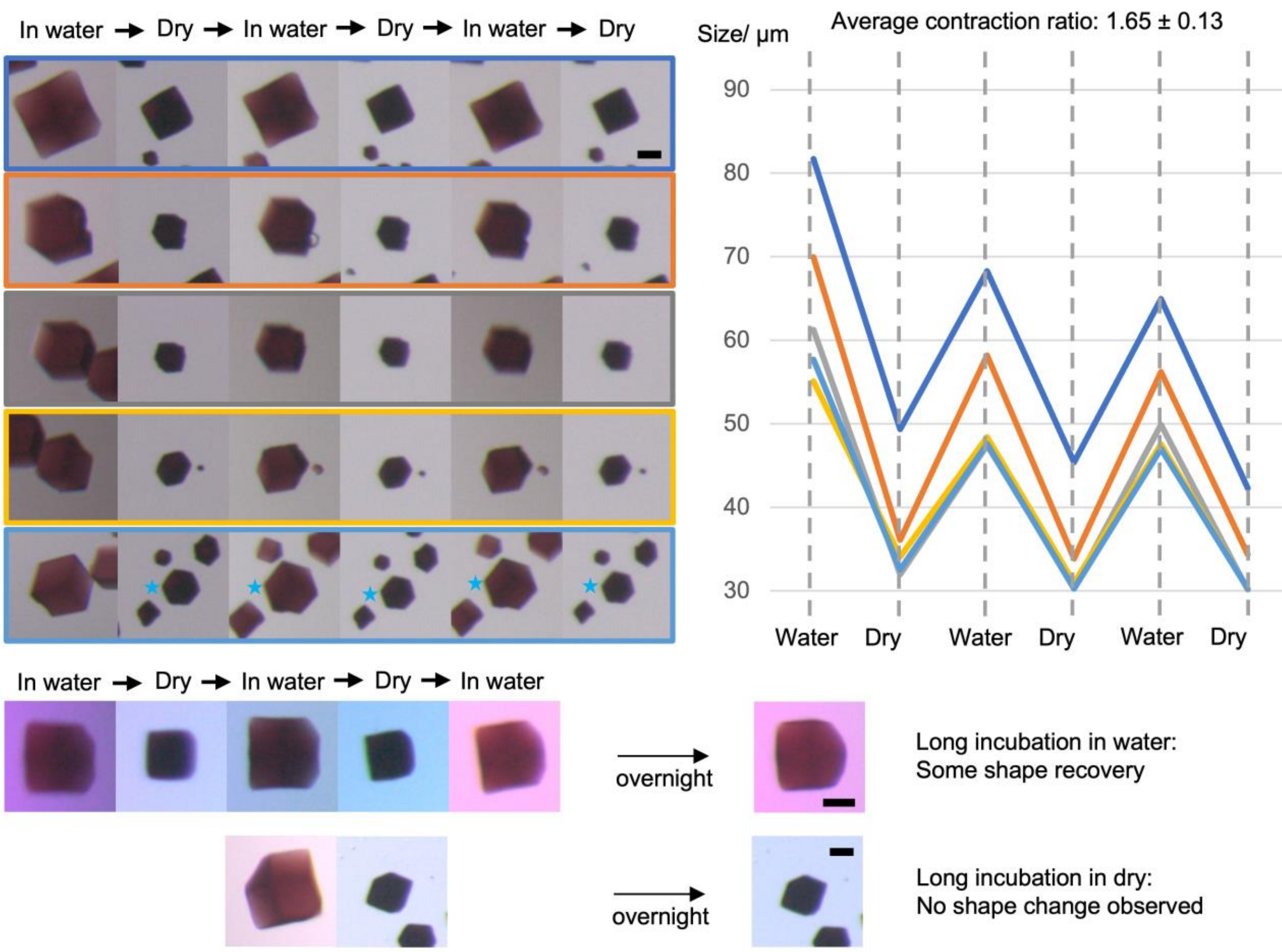

Supplementary Figure 23. Drying and rehydration cycles of crosslinked crystals in water with 13% AuNP encapsulation. During three cycles, different crystals were tracked for their size (measured as the diagonal distance) by the optical micrographs on the left. For all the contractions measured, the crystals contracted 1.65 ± 0.13 times on average. On the bottom panel, the crystals were observed for hydration and drying overnight. It took a relatively long time for the crystals to fully expand during the rehydration process, while the drying process completed in minutes. Scale bars, 20 µm.

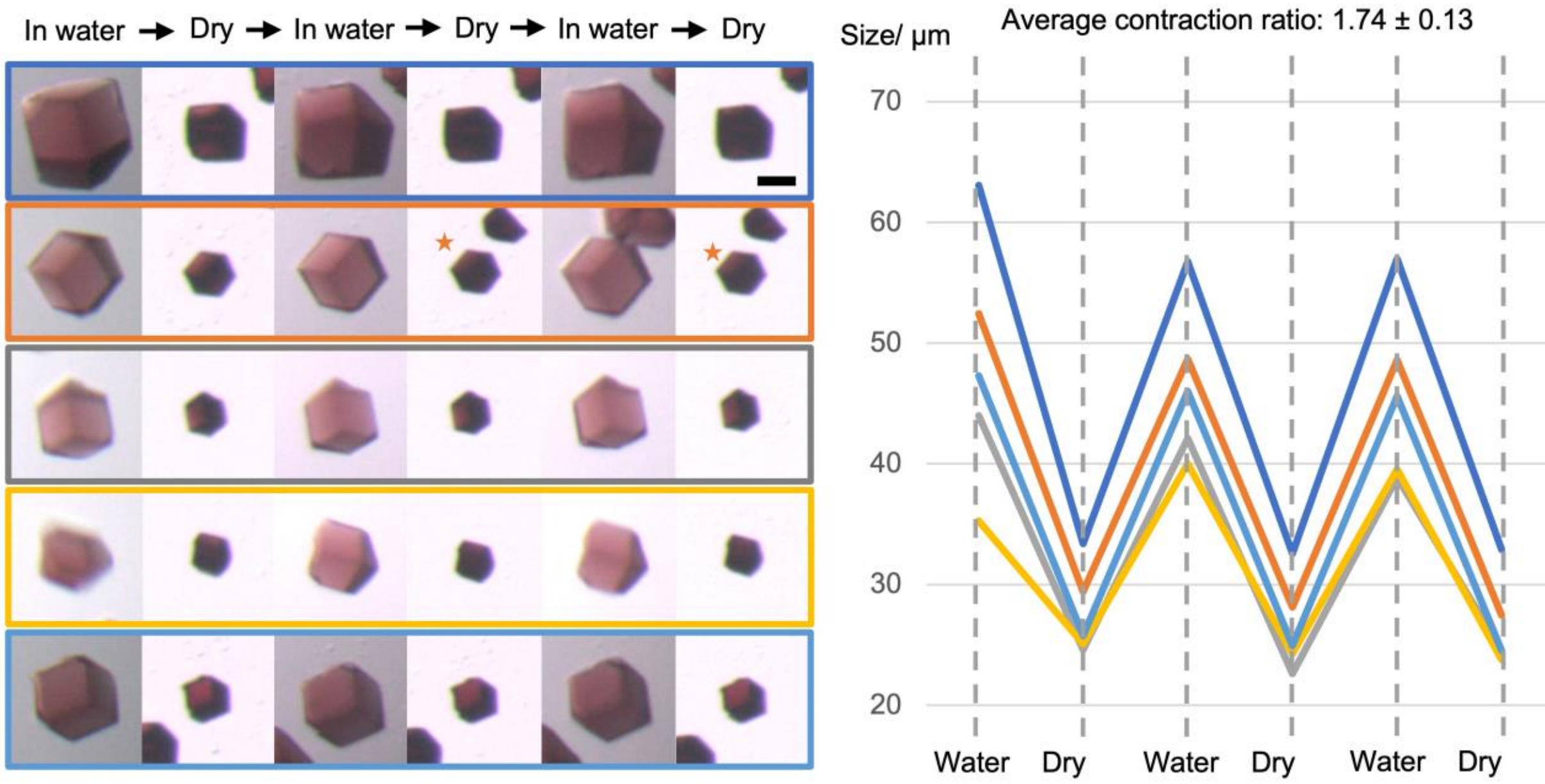

Supplementary Figure 24. Drying and rehydration cycles of crosslinked crystals in water with 5% AuNP encapsulation. During three cycles, different crystals were tracked for their size (measured as the diagonal distance) by the optical micrographs on the left. For all the contractions measured, the crystals contracted 1.74 ± 0.13 times on average. Scale bars, 20 µm.

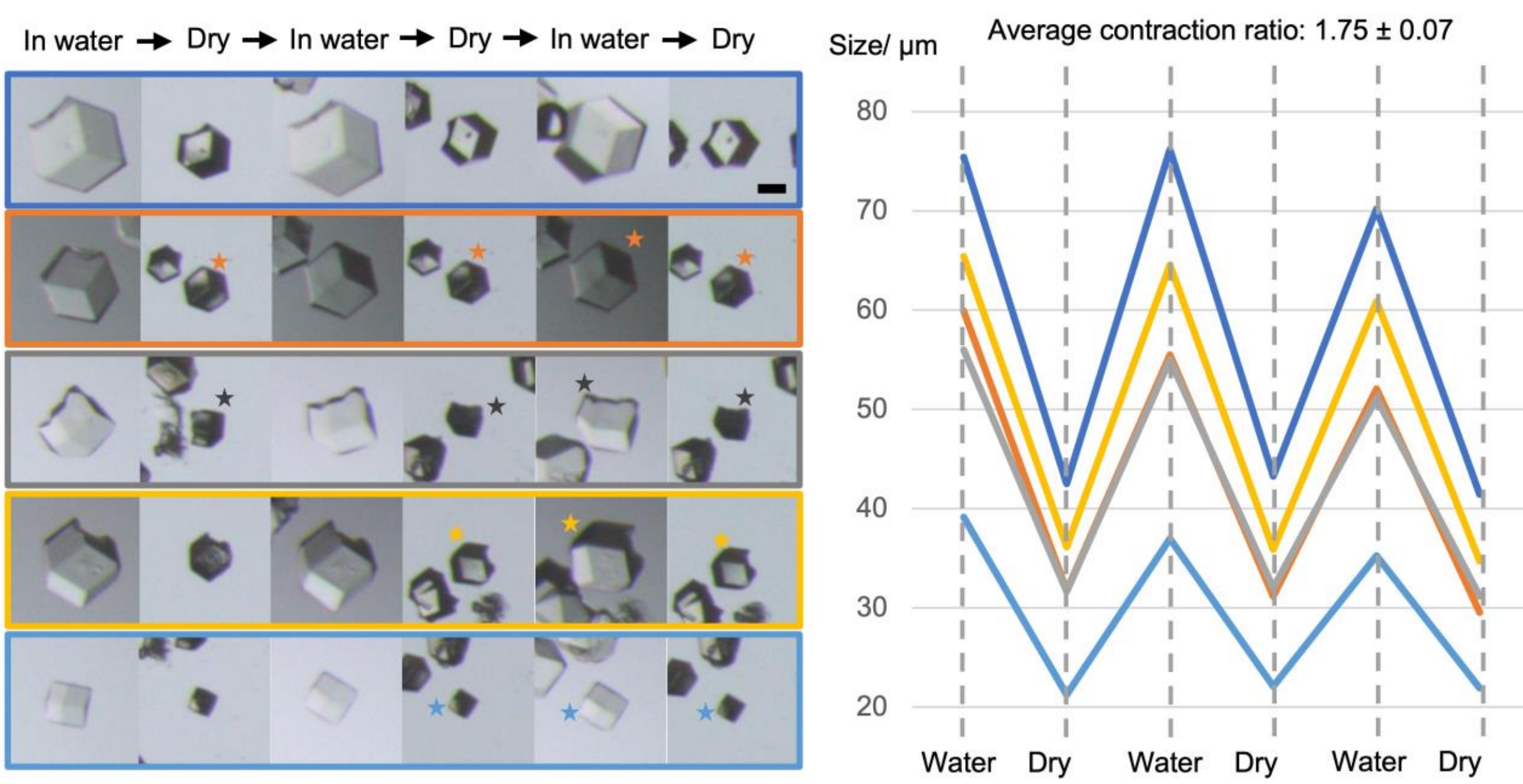

Supplementary Figure 25. Drying and rehydration cycles of crosslinked crystals in water without AuNP encapsulation. During three cycles, different crystals were tracked for their size (measured as the diagonal distance) by the optical micrographs on the left. For all the contractions measured, the crystals contracted 1.75 ± 0.07 times on average. Scale bars, 20 µm.

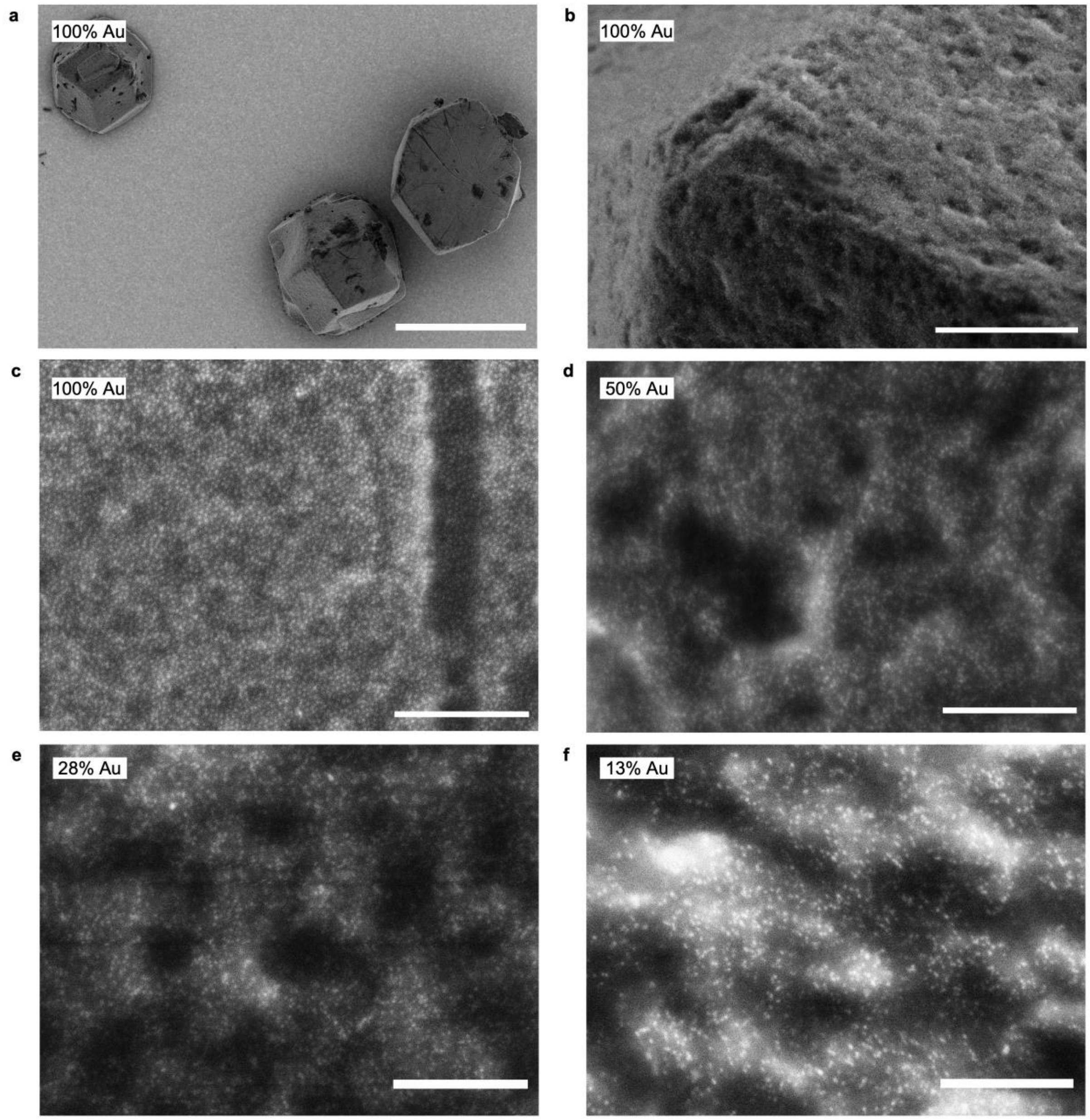

Supplementary Figure 26. Scanning electron micrographs of dried, crosslinked crystals with gold nanoparticle encapsulation. a-c, Micrographs of 100% gold encapsulated crystals with different magnifications. The scale bars are 20 mm, 1 mm and 200 nm, respectively. **c-f,** Micrographs of gold nanoparticle patterns on crystals with different nanoparticle encapsulation ratios. Individual nanoparticles are visible and their apparent density in the micrographs decreases with lower encapsulation ratio. Scale bar, 200 nm.

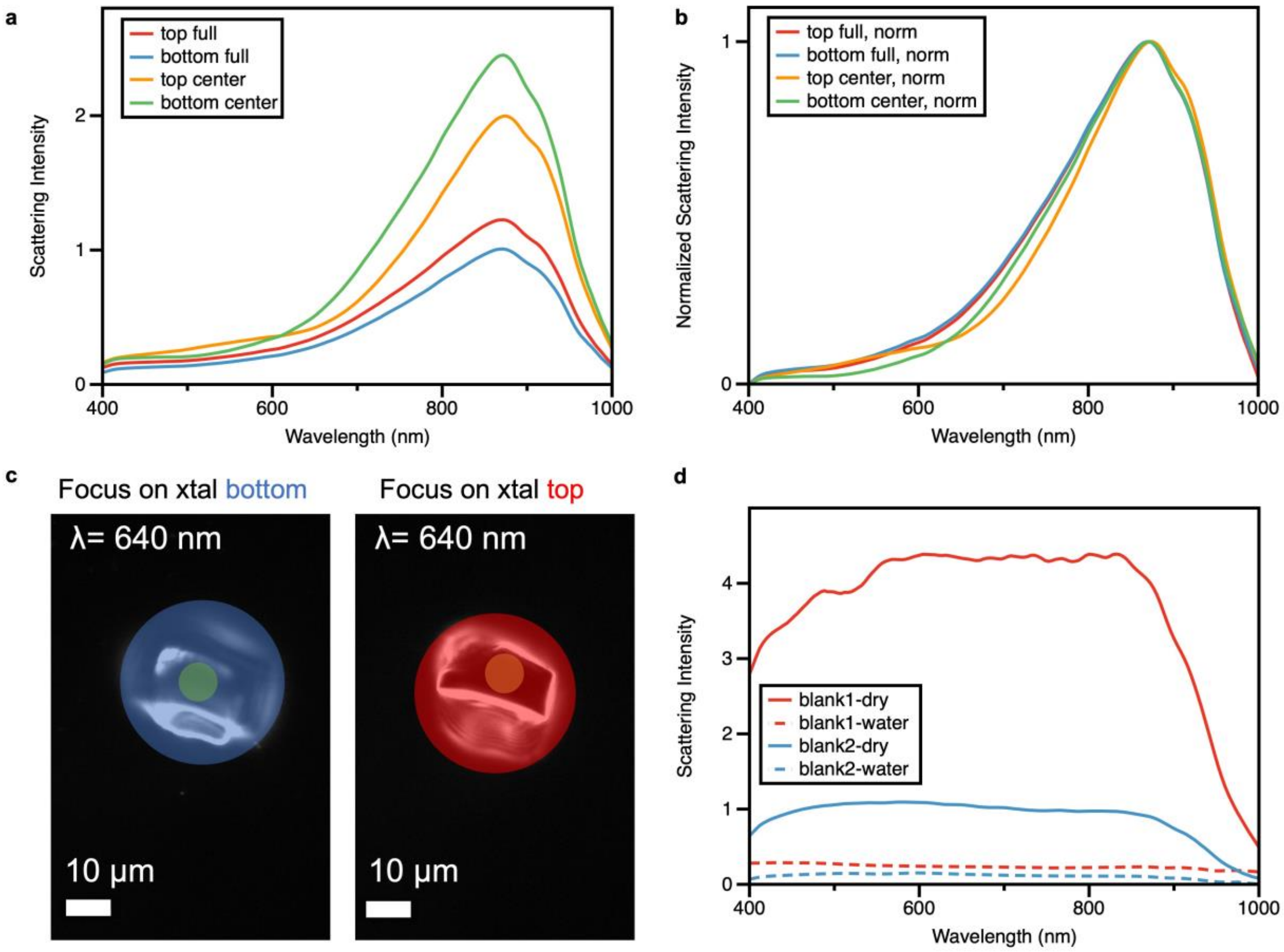

Supplementary Figure 27. Control hyperspectral dark field scattering spectra for crystal measurements. a-c, Hyperspectral scattering at different imaging focus height of the same crystal, with different area selection for averaging. For the imaging focus and selections in **c**, the scattering intensity varied (**a**) while the peak position was maintained for spectra with normalized intensity (**b**). **d**, Crystals without gold nanoparticles exhibited no characteristic scattering peaks for both dry and hydrated states.

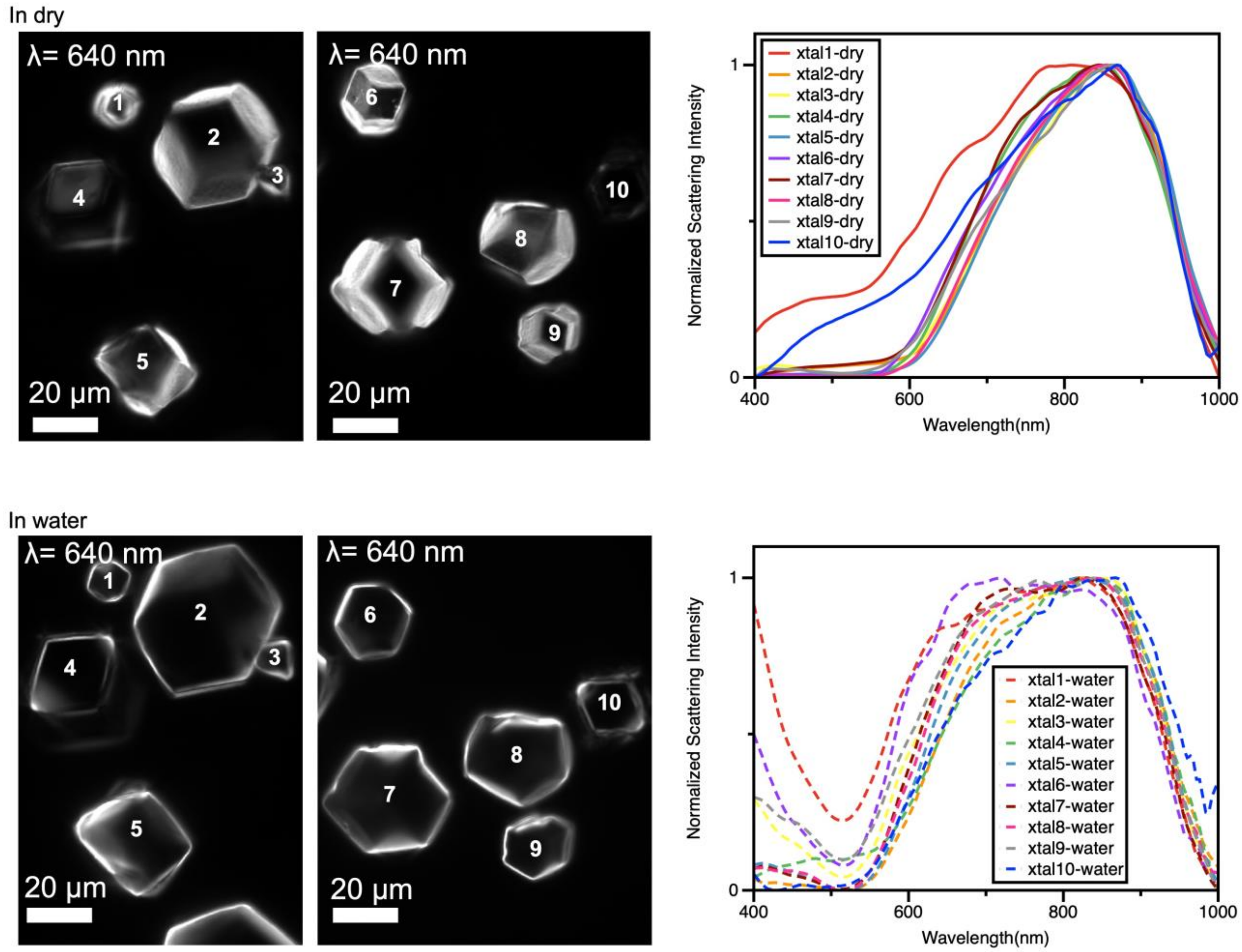

Supplementary Figure 28. Hyperspectral dark field scattering spectra for different crystals with the same composition. Ten crystals with 13% gold nanoparticle encapsulation of varying size and orientations resulted in similar spectra in both dry and hydrated states.

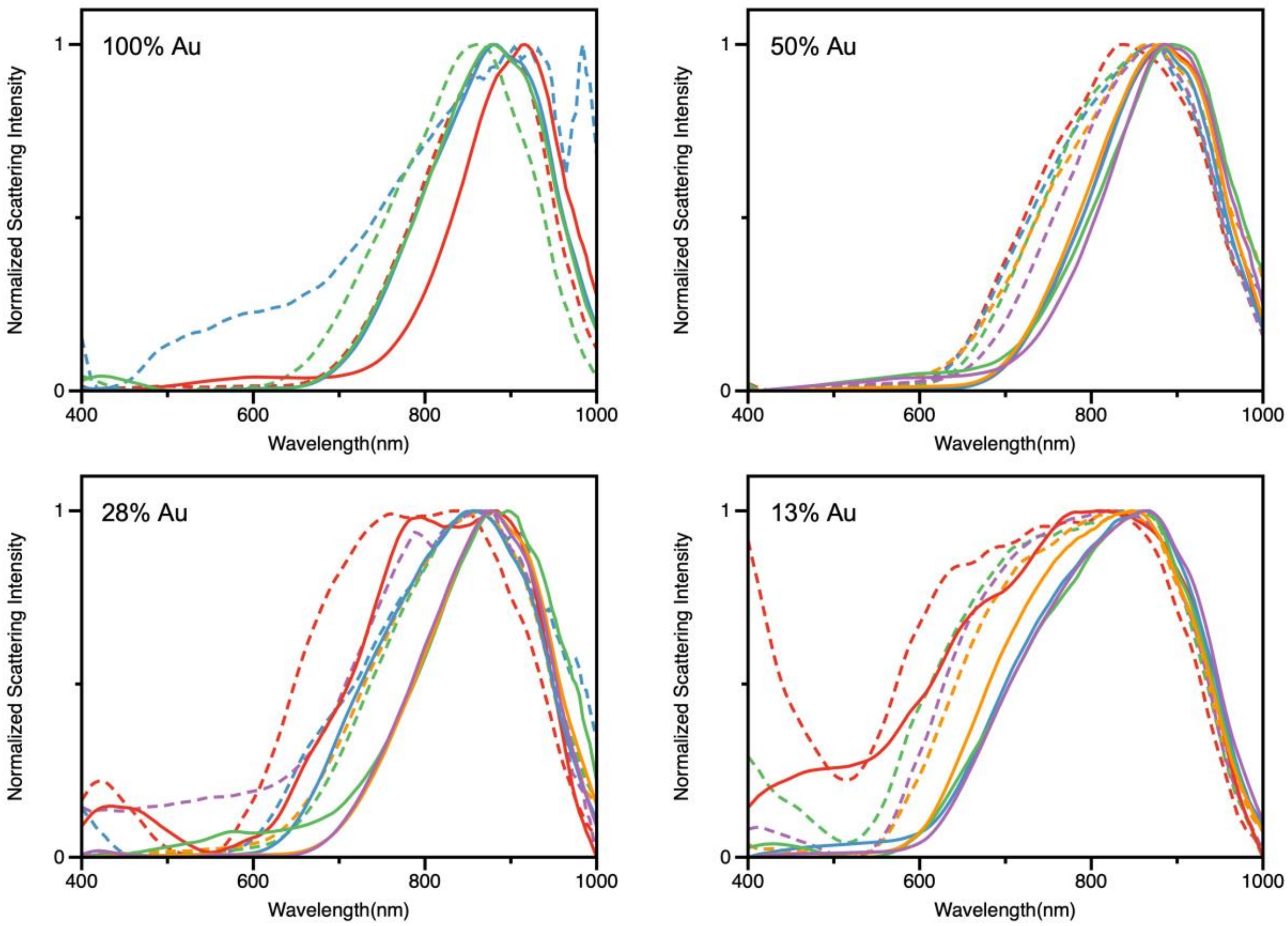

Supplementary Figure 29. Hyperspectral dark field scattering spectra for crystals with different gold nanoparticle ratios in dry (solid lines) and hydrated states (dashed line). For each ratio, spectra of 3 to 5 different crystals were plotted to demonstrate the general trend of scattering.

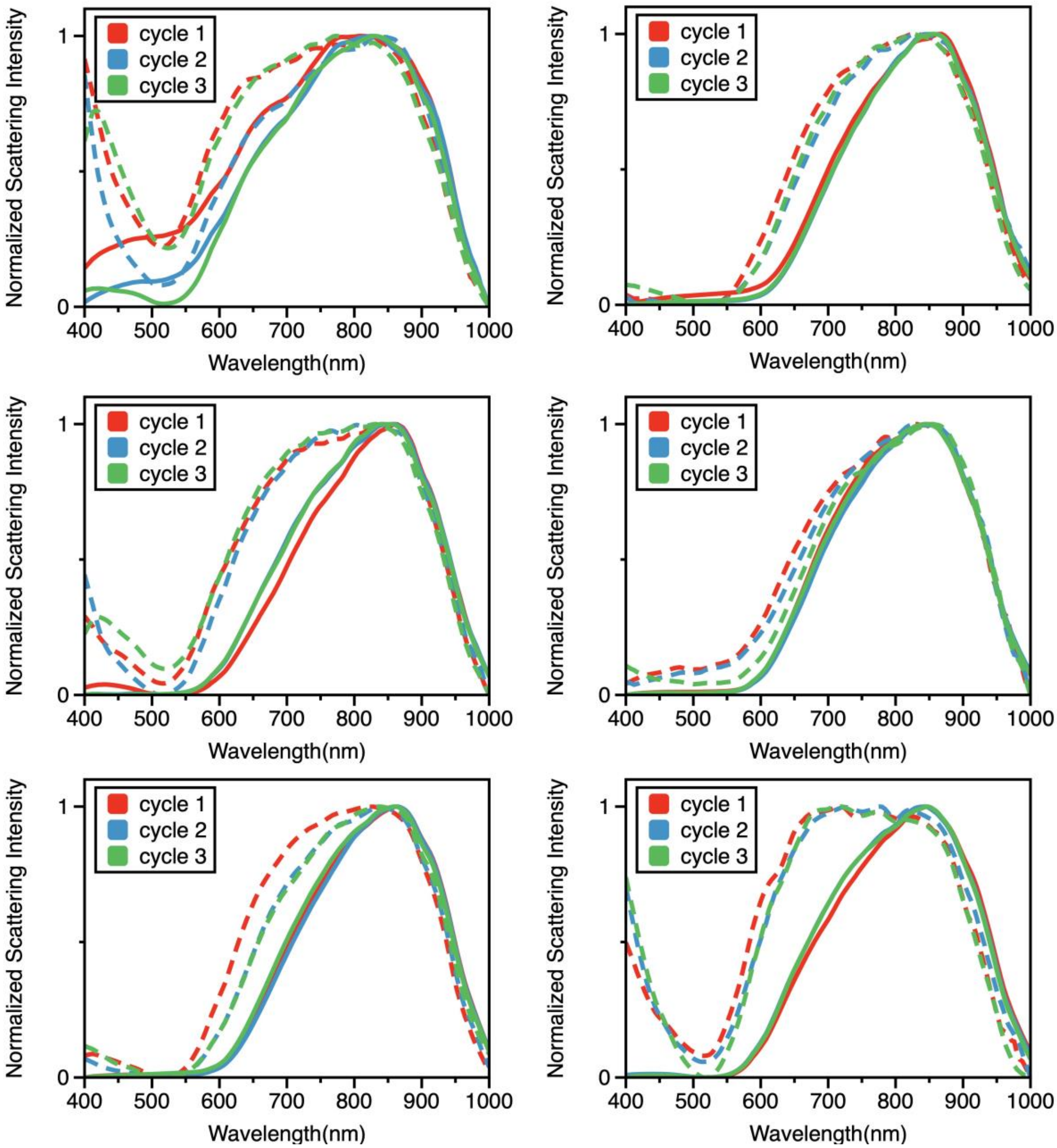

Supplementary Figure 30. Hyperspectral dark field scattering spectra for crystals with 13% gold nanoparticles over three cycles of dry (solid lines) and rehydration (dashed lines).

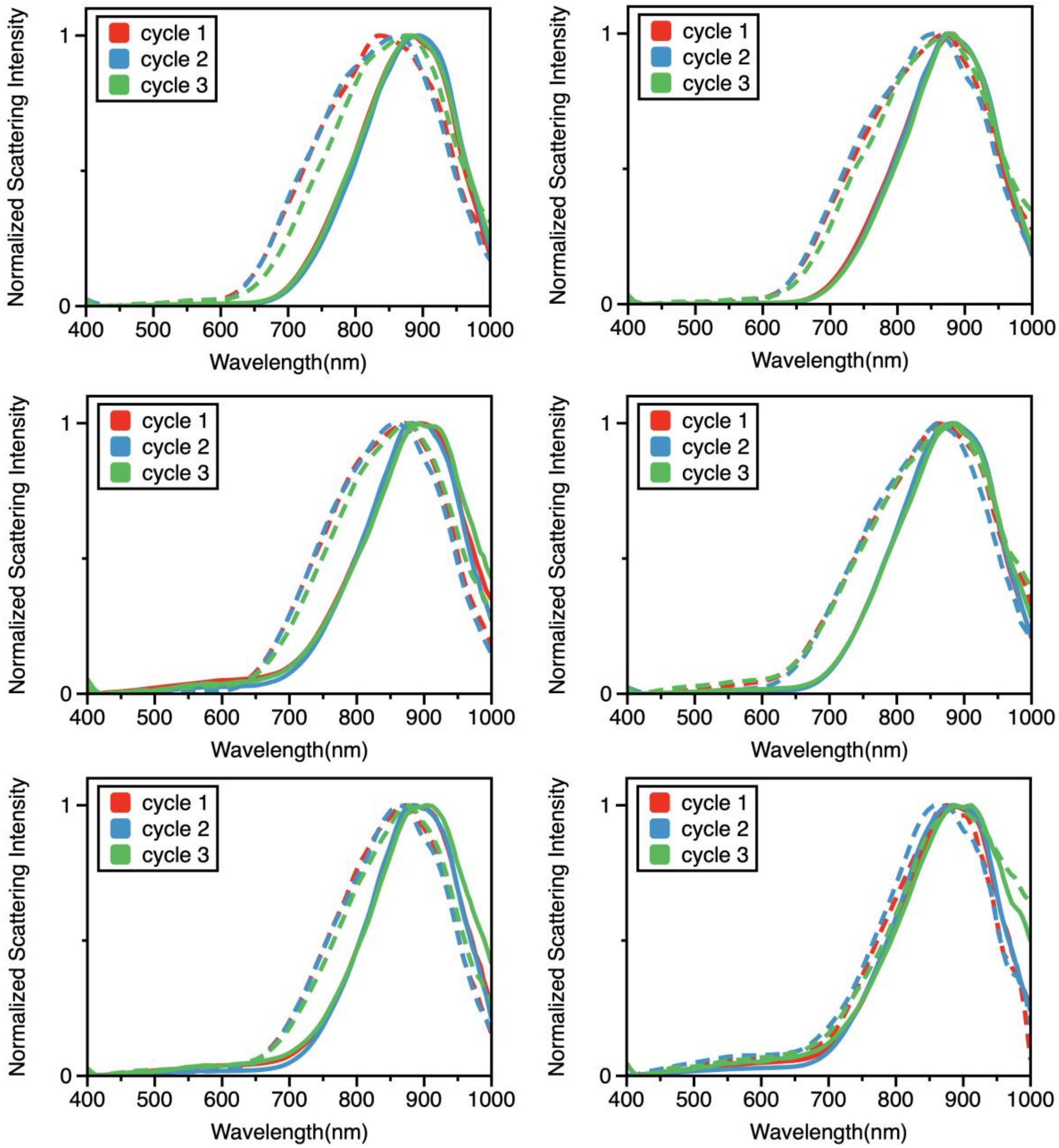

Supplementary Figure 31. Hyperspectral dark field scattering spectra for crystals with 50% gold nanoparticles over three cycles of dry (solid lines) and rehydration (dashed lines).

Gold Nanoparticle Occupancy (%)	Hydrated Volume Fraction (%Au)	Dehydrated Volume Fraction (%Au)
100	1.40	6.30
50	0.70	3.15
28	0.39	1.76
13	0.18	0.82

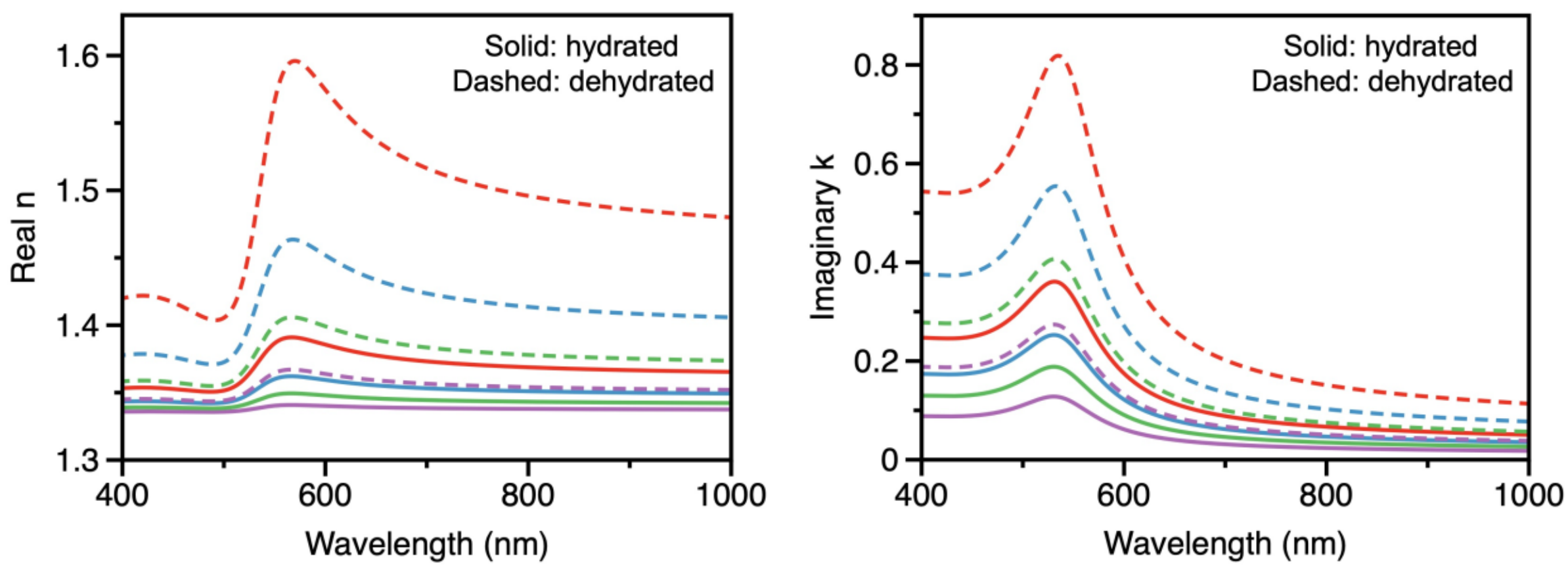

Supplementary Figure 32. Simulation of the refractive index of AuNP encapsulated crystals by effective medium theory (EMT). The top table summarizes the gold volume fractions in different crystals used for this study. The refractive indices are simulated as the real part n (left, bottom plot) and the imaginary part k (right, bottom plot), describing light dispersion and light loss/absorption (plasmonic here) respectively. When samples go from hydration state (solid lines) to dry state (dashed lines), there is clear increase in n at the near infrared wavelength regions, enhancing the photonic scattering. When the Au occupancy increases from 13 to 100%, n also significantly increases for more scattering.

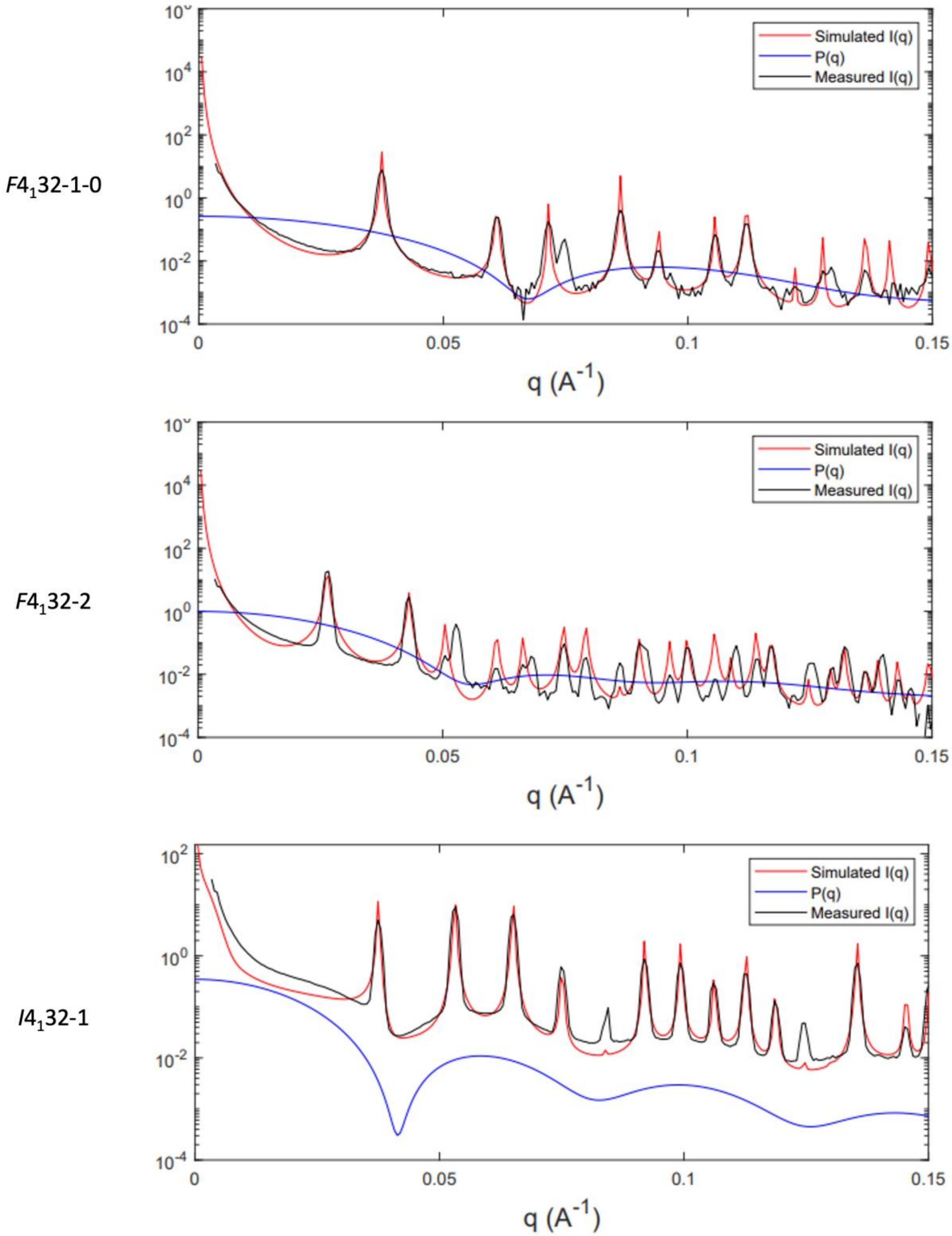

Supplementary Figure 33. SAXS simulation of protein cage form factor and calculated crystal intensity as compared to experimental data. P(q) represents the calculated form factor of individual cages.

Supplementary Table 1. Crystallization conditions of all designs

“Mixing” refers to directly mixing the two cage components in their purification buffer of 150 mM NaCl, 25 mM Tris pH 8.0. “Hanging drops” and “batch” crystallizations were previously described in the methods section.

Design name	Method	Cage concentration	NaCl concentration	Time	Crystal Size
$F4_132$-1-0	hanging drop	50 µM	2.5-3 M	in a week	~100 µm
$F4_132$-1	mixing	25 µM	-	overnight	~100 µm
$F4_132$-2-6H	hanging drop	50 µM	1-2 M	3-4 days	~100 µm
$F4_132$-2	hanging drop	50 µM	1-2 M	3-4 days	~100 µm
$F4_132$-2	batch	50-100 µM	5 M	overnight	~10 µm
*I*432-1	mixing	25 µM	-	overnight	~100 µm
*I*432-1	mixing at 50 mM NaCl	25 µM	-	in a week	~100 µm
*I*432-1-CC	hanging drop without extra NaCl	25-100 µM	0.5 M NaCl	3-4 days	~100 µm
*I*432-1-CC	batch	25-50 µM	0.5-1 M NaCl	minutes	~10 µm
$F4_132$-1-2	hanging drop	50 µM	4 M NaCl	in a week	20-50 µm
$F4_132$-1-3	hanging drop	50 µM	2.5-3 M	in a week	~100 µm
$F4_132$-2-1	hanging drop	50 µM	3 M NaCl	3-4 days	20-50 µm
$F4_132$-2-3	hanging drop	50 µM	3 M NaCl	3-4 days	20-50 µm
$F4_132$-2-12	hanging drop	50 µM	0.5 M	3-4 days	~100 µm
$F4_132$-2-14	hanging drop	50 µM	0.5 NaCl	3-4 days	20-50 µm
$F4_132$-2-15	hanging drop	50 µM	1 M NaCl	overnight	~10 µm
*I*432-1-7	mixing	25 µM	-	minutes	~10 µm
*I*432-1-8	hanging drop	25 µM	1 M NaCl	3-4 days	20-50 µm
*I*432-1-X3	hanging drop	25 µM	0.5 M NaCl	3-4 days	~100 µm
*I*432-1-X6	mixing	25 µM	-	minutes	~10 µm

*I*432-1-X9	hanging drop	25 µM	1 M NaCl	3-4 days	20-50 µm
$F4_1$32-2-ex1	mixing	25 µM	-	overnight	20-50 µm
$F4_1$32-2-ex2	hanging drop	10-25 µM	0.5 M NaCl	3-4 days	20-50 µm
$F4_1$32-2-ex3	hanging drop	10-25 µM	0.5 M NaCl	3-4 days	20-50 µm

Supplementary Table 2. Experimental tested mutagenesis of crystal contacts

Design name	Mutations	Soluble component?	Crystallization?
$F4_132$-1	T56R	Y	Y, 150 mM NaCl
$F4_132$-1-2	T56A	Y	Y, 4 M NaCl
$F4_132$-1-3	I59V	Y	Y, 3 M NaCl
$F4_132$-1-4	I64V	Y	N, ppts in 5 M NaCl
$F4_132$-1-5	I59V, I64V	Y	N, ppts in 5 M NaCl
$F4_132$-1-6	D63A	N	-
$F4_132$-1-7	T56A, D63M	N	-
$F4_132$-1-8	D63M	N	-
$F4_132$-1-9	K52D	N	-
$F4_132$-1-10	K60E	Y	N, ppts in 5 M NaCl
$F4_132$-1-11	R124D	N	-
$F4_132$-1-12	K50D, R124D	N	-
$F4_132$-1-13	E55K, D63A, D93K	N	-
$F4_132$-2-1	A6S	Y	Y, 3 M NaCl
$F4_132$-2-2	L13V	Y	N, clear
$F4_132$-2-3	L9I	Y	Y, 3 M NaCl
$F4_132$-2-4	L9V	Y	N, clear
$F4_132$-2-5	L9V, L13I	Y	N, clear
$F4_132$-2-6	E16L, R20D	Y	N, ppts
$F4_132$-2-7	E16I, R20D	Y	Y, microcrystals
$F4_132$-2-8	E16I, R20Q	Y	N, polycrystals
$F4_132$-2-9	Ntrim2-4, A6D, E16I, R20Q	Y	N, polycrystals
$F4_132$-2-10	Ntrim2-4, A6D, L9S, E16S, R20Q	Y	N, clear

$F4_132$-2-11	Ntrim2-4, A6D, L9T, E16I, R20Q	Y	N, clear
$F4_132$-2-12	Ntrim2-4, A6D, E16T, R20Q	Y	Y, 0.5 M NaCl
$F4_132$-2-13	Ntrim2-4, A6D, L9T, E16T, R20Q	Y	N, clear
$F4_132$-2-14	Ntrim2-4, A6D, E16A, R20Q	Y	Y, 0.5 M NaCl
$F4_132$-2-15	Ntrim2-4, A6D, E16T, R20N	Y	Y, 1 M NaCl
$F4_132$-2-16	Ntrim2-4, A6D, E16T, R20D	Y	Y, 3 M NaCl
*I*432-2-1	Q88N, N114N	Y	N, clear
*I*432-2-2	Q88A, N114A	N	-
*I*432-2-3	Q88S, N114S	Y	N, clear
*I*432-2-4	Q88T, N114T	Y	N, clear
*I*432-2-5	Q88L, N114L	Y	N, clear
*I*432-2-6	Q88C, N114C	N	-
*I*432-2-7	Q88K, N114E	Y	Y, 150 mM NaCl

Supplementary Table 3. Protein sequences experimentally tested in this study

*His-tag, Trp for A280 absorbance, and N terminal Met are included in all ordered sequences

Name	Sequence*	Notes
T33-15A	MVRGIRGAITVNSDTPTSIIIATILLLEKMLEANGIQSYEELAAV IFTVTEDLTSAFPAEAARQIGMHRVPLLSAREVPVPGSLPRVIRV LALWNTDTPQDRVRHVYLSEAVRLRPDLESAQLEHHHHHH	T33-15 cage C3-A/ $F4_1 32$-1 chain A
T33-15B	MSKAKIGIVTVSDRASAGITADISGKAIILALNLYLTSEWEPIYQ VIPDEQDVIETTLIKMADEQDCCLIVTTGGTGPAKRDVTPEATEA VCDRMMPGFGELMRAESLKEVPTAILSRQTAGLRGDSLIVNLPGD PASISDCLLAVFPAIPYCIDLMEGPYLECNEAMIKPFRPKAKLEH HHHHH	T33-15 cage C3-B
T33-15-D3-4B	MSKAKIGIVTVSDRASAGITADISGKAIILALNLYLTSEWEPIYQ VIPDEQKVIETTLIKMADIQDCCLIVTTGGTGPAKRDVTPEATEA VCDRMMPGFGELMRAESLKEVPTAILSRQTAGLRGDSLIVNLPGD PASISDCLLAVFPAIPYCIDLMEGPYLECNEAMIKPFRPKAKLEH HHHHH	$F4_1 32$-1-0 chain B
T33-15-D3-4B-1	MSKAKIGIVTVSDRASAGITADISGKAIILALNLYLTSEWEPIYQ VIPDEQKVIERTLIKMADIQDCCLIVTTGGTGPAKRDVTPEATEA VCDRMMPGFGELMRAESLKEVPTAILSRQTAGLRGDSLIVNLPGD PASISDCLLAVFPAIPYCIDLMEGPYLECNEAMIKPFRPKAKLEH HHHHH	$F4_1 32$-1 chain B
T33-15-D3-4B-2	MSKAKIGIVTVSDRASAGITADISGKAIILALNLYLTSEWEPIYQ VIPDEQKVIEATLIKMADIQDCCLIVTTGGTGPAKRDVTPEATEA VCDRMMPGFGELMRAESLKEVPTAILSRQTAGLRGDSLIVNLPGD PASISDCLLAVFPAIPYCIDLMEGPYLECNEAMIKPFRPKAKLEH HHHHH	$F4_1 32$-1-2 chain B
T33-15-D3-4B-3	MSKAKIGIVTVSDRASAGITADISGKAIILALNLYLTSEWEPIYQ VIPDEQKVIETTLVKMADIQDCCLIVTTGGTGPAKRDVTPEATEA VCDRMMPGFGELMRAESLKEVPTAILSRQTAGLRGDSLIVNLPGD PASISDCLLAVFPAIPYCIDLMEGPYLECNEAMIKPFRPKAKLEH HHHHH	$F4_1 32$-1-3 chain B
T33-15-D3-4B-10	MSKAKIGIVTVSDRASAGITADISGKAIILALNLYLTSEWEPIYQ VIPDEQKVIETTLIEMADIQDCCLIVTTGGTGPAKRDVTPEATEA VCDRMMPGFGELMRAESLKEVPTAILSRQTAGLRGDSLIVNLPGD PASISDCLLAVFPAIPYCIDLMEGPYLECNEAMIKPFRPKAKLEH HHHHH	$F4_1 32$-1-10 chain B

T33-15-D3-6B	MSKAKIGIVTVSDRASAGITADISGKAIILALNLYLTSEWEPIYQVIPDEQDVIEATLSIMADLQDCCLIVTTGGTGPAKRDVTPEATEAVCDRMMPGFGELMRAESLKEVPTAILSRQTAGLRGDSLIVNLPGDPASISDCLLAVFPAIPYCIDLMEGPYLECNEAMIKPFRPKAKLEHHHHHH	*R*3-1 chain B
2L4HC2_23	TRTEIIRELERSLREQEELAKRLKELLRELERLQREGSSDEDVRELLREIKELVEEIEKLAREQKYLVEELKRQ	C2 scaffold for T32-15A
HFuse_pH192_0046	DEAEEKARRVAEKVERLKRSGTSEDEIAEEVAREISEVIRTLKESGSSYEVIAEIVARIVAEIVEALKRSGTSEDEIAEIVARVISEVIRTLKESGSSYEVIAEIVARIVAEIVEALKRSGTSEDEIAEIVARVISEVIRTLKESGSSYEVIAEIVARIVAEIVEALKRSGTSEDEIAEIVARVISEVIRTLKESGSSYEVIAEIVARIVAEIVEALKRSGTSEDEIAEIVARVISEVIRTLKESGSSYEVIAEIVARIVAEIVEALKRSGTSEDEIAEIVARVISEVIRTLKESGSSYEVIAEIVARIVAEIVEALLRSGTSEEEIAKIVARVMNEVLRTLRESGSDFEVIREILRRILEEINEALKRGGVSEDEIMRIEIKILLMLLRLSTAELERATRSLKAITEELKKNPSEDALVEHNRAIVEHNRIIVFNNILIALVLEAIVRAIK	C3 scaffold for T32-15B
T32-15A	MTRTEIIRELERSLREQEELAKRLMELLLKLLRLQMTGSSDEDVRRLMLRIIELVEEIEELAREQKYLVEELKRQGSWSGLEHHHHHH	T32-15 cage C2 (8 heptads)
T32-15B	MGSWSGHHHHHHGSSDEAEEKARRVAEKVERLKRSGTSEDEIAEEVAREISEVIRTLKESGSSYEVIAEIVARIVAEIVEALKRSGTSEDEIAEIVARVISEVIRTLKESGSSYEVIAEIVARIVAEIVEALKRSGTSEDEIAEIVARVISEVIRTLKESGSSYEVIAEIVARIVAEIVEALKRSGTSEDEIAEIVARVISEVIRTLKESGSSAEVIAEIVARIVAEIVEALKRSGTSEDEIAEIVARVISEVIRTLKESGSSSILIALIVARIVAEIVEALKRSGTSEDEIAEIVARVISEVIRTLKESGSSYEIIALIVAMIVAEIVRALLRSGTSEEEIAKIVARVMNEVLRTLRESGSDFEVIREILRLILAAIRAALQKGGVSEDEIMRIEIKILLMLLRLSTAELERATRSLKAITEELKKNPSEDALVEHNRAIVEHNRIIVFNNILIALVLEAIVRAIK	T32-15 cage C3
T32-15-6HA	MRSLREQEELAKRLMELLLKLLRLQMTGSSDEDVRRLMLRIIELVEEIEELAREQKGSWSGLEHHHHHH	T32-15 cage C2 (6 heptads)\ *F*4$_1$32-2 chain A
T32-15-5HA	MQEELAKRLMELLLKLLRLQMTGSSDEDVRRLMLRIIELVEEIEEGSWSGLEHHHHHH	T32-15 cage C2 (5 heptads)
T32-15-D3-6B	MDEAEAKALRVALKVEELKRSGTSEDEIAEEVAREISEVIRTLKESGSSYEVIAEIVARIVAEIVEALKRSGTSEDEIAEIVARVISEVIRTLKESGSSYEVIAEIVARIVAEIVEALKRSGTSEDEIAEIVARV	*F*4$_1$32-2 chain B

	ISEVIRTLKESGSSYEVIAEIVARIVAEIVEALKRSGTSEDEIAE<br>IVARVISEVIRTLKESGSSAEVIAEIVARIVAEIVEALKRSGTSE<br>DEIAEIVARVISEVIRTLKESGSSSILIALIVARIVAEIVEALKR<br>SGTSEDEIAEIVARVISEVIRTLKESGSSYEIIALIVAMIVAEIV<br>RALLRSGTSEEEIAKIVARVMNEVLRTLRESGSDFEVIREILRLI<br>LAAIRAALQKGGVSEDEIMRIEIKILLMLLRLSTAELERATRSLK<br>AITEELKKNPSEDALVEHNRAIVEHNRIIVFNNILIALVLEAIVR<br>AIKGSWGSLEHHHHHH	
T32-15-D3-6B-1	MDEAESKALRVALKVEELKRSGTSEDEIAEEVAREISEVIRTLKE<br>SGSSYEVIAEIVARIVAEIVEALKRSGTSEDEIAEIVARVISEVI<br>RTLKESGSSYEVIAEIVARIVAEIVEALKRSGTSEDEIAEIVARV<br>ISEVIRTLKESGSSYEVIAEIVARIVAEIVEALKRSGTSEDEIAE<br>IVARVISEVIRTLKESGSSAEVIAEIVARIVAEIVEALKRSGTSE<br>DEIAEIVARVISEVIRTLKESGSSSILIALIVARIVAEIVEALKR<br>SGTSEDEIAEIVARVISEVIRTLKESGSSYEIIALIVAMIVAEIV<br>RALLRSGTSEEEIAKIVARVMNEVLRTLRESGSDFEVIREILRLI<br>LAAIRAALQKGGVSEDEIMRIEIKILLMLLRLSTAELERATRSLK<br>AITEELKKNPSEDALVEHNRAIVEHNRIIVFNNILIALVLEAIVR<br>AIKGSWGSLEHHHHHH	$F4_{1}32$-2-1 chain B
T32-15-D3-6B-3	MDEAEAKAIRVALKVEELKRSGTSEDEIAEEVAREISEVIRTLKE<br>SGSSYEVIAEIVARIVAEIVEALKRSGTSEDEIAEIVARVISEVI<br>RTLKESGSSYEVIAEIVARIVAEIVEALKRSGTSEDEIAEIVARV<br>ISEVIRTLKESGSSYEVIAEIVARIVAEIVEALKRSGTSEDEIAE<br>IVARVISEVIRTLKESGSSAEVIAEIVARIVAEIVEALKRSGTSE<br>DEIAEIVARVISEVIRTLKESGSSSILIALIVARIVAEIVEALKR<br>SGTSEDEIAEIVARVISEVIRTLKESGSSYEIIALIVAMIVAEIV<br>RALLRSGTSEEEIAKIVARVMNEVLRTLRESGSDFEVIREILRLI<br>LAAIRAALQKGGVSEDEIMRIEIKILLMLLRLSTAELERATRSLK<br>AITEELKKNPSEDALVEHNRAIVEHNRIIVFNNILIALVLEAIVR<br>AIKGSWGSLEHHHHHH	$F4_{1}32$-2-3 chain B
T32-15-D3-6B-7	MDEAEAKALRVALKVIELKDSGTSEDEIAEEVAREISEVIRTLKE<br>SGSSYEVIAEIVARIVAEIVEALKRSGTSEDEIAEIVARVISEVI<br>RTLKESGSSYEVIAEIVARIVAEIVEALKRSGTSEDEIAEIVARV<br>ISEVIRTLKESGSSYEVIAEIVARIVAEIVEALKRSGTSEDEIAE<br>IVARVISEVIRTLKESGSSAEVIAEIVARIVAEIVEALKRSGTSE<br>DEIAEIVARVISEVIRTLKESGSSSILIALIVARIVAEIVEALKR<br>SGTSEDEIAEIVARVISEVIRTLKESGSSYEIIALIVAMIVAEIV<br>RALLRSGTSEEEIAKIVARVMNEVLRTLRESGSDFEVIREILRLI<br>LAAIRAALQKGGVSEDEIMRIEIKILLMLLRLSTAELERATRSLK<br>AITEELKKNPSEDALVEHNRAIVEHNRIIVFNNILIALVLEAIVR<br>AIKGSWGSLEHHHHHH	$F4_{1}32$-2-7 chain B
T32-15-D3-6B-8	MDEAEAKALRVALKVIELKQSGTSEDEIAEEVAREISEVIRTLKE<br>SGSSYEVIAEIVARIVAEIVEALKRSGTSEDEIAEIVARVISEVI<br>RTLKESGSSYEVIAEIVARIVAEIVEALKRSGTSEDEIAEIVARV<br>ISEVIRTLKESGSSYEVIAEIVARIVAEIVEALKRSGTSEDEIAE	$F4_{1}32$-2-8 chain B

	IVARVISEVIRTLKESGSSAEVIAEIVARIVAEIVEALKRSGTSE DEIAEIVARVISEVIRTLKESGSSSILIALIVARIVAEIVEALKR SGTSEDEIAEIVARVISEVIRTLKESGSSYEIIALIVAMIVAEIV RALLRSGTSEEEIAKIVARVMNEVLRTLRESGSDFEVIREILRLI LAAIRAALQKGGVSEDEIMRIEIKILLMLLRLSTAELERATRSLK AITEELKKNPSEDALVEHNRAIVEHNRIIVFNNILIALVLEAIVR AIKGSWGSLEHHHHHH	
T32-15-D3-6B-12	MEDKALRVALKVTELKQSGTSEDEIAEEVAREISEVIRTLKESGS SYEVIAEIVARIVAEIVEALKRSGTSEDEIAEIVARVISEVIRTL KESGSSYEVIAEIVARIVAEIVEALKRSGTSEDEIAEIVARVISE VIR TLKESGSSYEVIAEIVARIVAEIVEALKRSGTSEDEIAEIVARVI SEVIRTLKESGSSAEVIAEIVARIVAEIVEALKRSGTSEDEIAEI VARVISEVIRTLKESGSSSILIALIVARIVAEIVEALKRSGTSED EIAEIVARVISEVIRTLKESGSSYEIIALIVAMIVAEIVRALLRS GTSEEEIAKIVARVMNEVLRTLRESGSDFEVIREILRLILAAIRA ALQKGGVSEDEIMRIEIKILLMLLRLSTAELERATRSLKAITEEL KKNPSEDALVEHNRAIVEHNRIIVFNNILIALVLEAIVRAIKGSW GSLEHHHHHH	*F*$4_1$32-2-12 chain B
T32-15-D3-6B-14	MEDKALRVALKVAELKQSGTSEDEIAEEVAREISEVIRTLKESGS SYEVIAEIVARIVAEIVEALKRSGTSEDEIAEIVARVISEVIRTL KESGSSYEVIAEIVARIVAEIVEALKRSGTSEDEIAEIVARVISE VIR TLKESGSSYEVIAEIVARIVAEIVEALKRSGTSEDEIAEIVARVI SEVIRTLKESGSSAEVIAEIVARIVAEIVEALKRSGTSEDEIAEI VARVISEVIRTLKESGSSSILIALIVARIVAEIVEALKRSGTSED EIAEIVARVISEVIRTLKESGSSYEIIALIVAMIVAEIVRALLRS GTSEEEIAKIVARVMNEVLRTLRESGSDFEVIREILRLILAAIRA ALQKGGVSEDEIMRIEIKILLMLLRLSTAELERATRSLKAITEEL KKNPSEDALVEHNRAIVEHNRIIVFNNILIALVLEAIVRAIKGSW GSLEHHHHHH	*F*$4_1$32-2-14 chain B
T32-15-D3-6B-15	MEDKALRVALKVTELKNSGTSEDEIAEEVAREISEVIRTLKESGS SYEVIAEIVARIVAEIVEALKRSGTSEDEIAEIVARVISEVIRTL KESGSSYEVIAEIVARIVAEIVEALKRSGTSEDEIAEIVARVISE VIR TLKESGSSYEVIAEIVARIVAEIVEALKRSGTSEDEIAEIVARVI SEVIRTLKESGSSAEVIAEIVARIVAEIVEALKRSGTSEDEIAEI VARVISEVIRTLKESGSSSILIALIVARIVAEIVEALKRSGTSED EIAEIVARVISEVIRTLKESGSSYEIIALIVAMIVAEIVRALLRS GTSEEEIAKIVARVMNEVLRTLRESGSDFEVIREILRLILAAIRA ALQKGGVSEDEIMRIEIKILLMLLRLSTAELERATRSLKAITEEL KKNPSEDALVEHNRAIVEHNRIIVFNNILIALVLEAIVRAIKGSW GSLEHHHHHH	*F*$4_1$32-2-15 chain B
T32-15-D3-6B-16	MEDKALRVALKVTELKDSGTSEDEIAEEVAREISEVIRTLKESGS SYEVIAEIVARIVAEIVEALKRSGTSEDEIAEIVARVISEVIRTL	*F*$4_1$32-2-16 chain B

	KESGSSYEVIAEIVARIVAEIVEALKRSGTSEDEIAEIVARVISEVIRTLKESGSSYEVIAEIVARIVAEIVEALKRSGTSEDEIAEIVARVISEVIRTLKESGSSAEVIAEIVARIVAEIVEALKRSGTSEDEIAEIVARVISEVIRTLKESGSSSILIALIVARIVAEIVEALKRSGTSEDEIAEIVARVISEVIRTLKESGSSYEIIALIVAMIVAEIVRALLRSGTSEEEIAKIVARVMNEVLRTLRESGSDFEVIREILRLILAAIRAALQKGGVSEDEIMRIEIKILLMLLRLSTAELERATRSLKAITEELKKNPSEDALVEHNRAIVEHNRIIVFNNILIALVLEAIVRAIKGSWGSLEHHHHHH	
T32-15-D3-6B-ex1	MEEIVEKAERKLKFLLQEAEEGGKEDALEIAEKLAELAKEALRVLAEAGGSPELMLRLMETAARALARIARLGGDELREEIKKIMAELVAKAISLLIRMLKRSGSSYEEIAEAVAKAVAKIVEAAKESGMSEDEIAEIVARVISEVIRTLKESGSSAEVIAEIVARIVAEIVEALKRSGTSEDEIAEIVARVISEVIRTLKESGSSSILIALIVARIVAEIVEALKRSGTSEDEIAEIVARVISEVIRTLKESGSSYEIIALIVAMIVAEIVRALLRSGTSEEEIAKIVARVMNEVLRTLRESGSDFEVIREILRLILAAIRAALQKGGVSEDEIMRIEIKILLMLLRLSTAELERATRSLKAITEELKKNPSEDALVEHNRAIVEHNRIIVFNNILIALVLEAIVRAIKGSWSGLEHHHHHH	$F4_1$32-2-ex1 chain B
T32-15-D3-6B-ex2	MDEKRKRAQKALERAQEALKKGDVEEAVRAAEEAVKAAAEAGDDEMLELVALQAEAIALAAQQQGNDEVKRKAKMVARAAKVAAEIIKLIKELKRNGASYEEIAEEVAKRVAEIVEELKKQGTSEEEIAFIVAAVIAAVIAALKKSGSSAEVIAEIVARIVAEIVEALKRSGTSEDEIAEIVARVISAVIRALKESGSSSILIALIVARIVAEIVEALKRSGTSEDEIAEIVARVISEVIRTLKESGSSYEIIALIVAMIVAEIVRALLRSGTSEEEIAKIVARVMNEVLRTLRESGSDFEVIREILRLILAAIRAALQKGGVSEDEIMRIEIKILLMLLRLSTAELERATRSLKAITEELKKNPSEDALVEHNRAIVEHNRIIVFNNILIALVLEAIVRAIGSWSGLEHHHHHH	$F4_1$32-2-ex2 chain B
T32-15-D3-6B-ex3	MDEKRKRAEKALQRAMKALEKGDVEEAVRAAEEAVKAAAEAGDDEMLKKVAAAAELIAMAAQQQGNDEVKRKAKMVARAAKVAAEIIKLIKELKRNGASYEEIAEEVAKRVAEIVEELKKQGTSEEEIAFIVAAVIAAVIAALKKSGSSAEVIAEIVARIVAEIVEALKRSGTSEDEIAEIVARVISAVIRALKESGSSSILIALIVARIVAEIVEALKRSGTSEDEIAEIVARVISEVIRTLKESGSSYEIIALIVAMIVAEIVRALLRSGTSEEEIAKIVARVMNEVLRTLRESGSDFEVIREILRLILAAIRAALQKGGVSEDEIMRIEIKILLMLLRLSTAELERATRSLKAITEELKKNPSEDALVEHNRAIVEHNRIIVFNNILIALVLEAIVRAIKGSWSGLEHHHHHH	$F4_1$32-2-ex3 chain B
tpr1C4_2	ALAYVMLGLLLSLLNRLSLAAEAYKKAIELDPNDALAWLLLGSVLEKLKRLDEAAEAYKKAIELKPNDASAWKELGKVLEKLGRLDEAAEAYKKAIELDPEDAEAWKELGKVLEKLGRLDEAAEAYKKAIELDPND	C4 scaffold for O43-2A

1wa3	KMEELFKKHKIVAVLRANSVEEAKEKALAVFEGGVHLIEITFTVP DADTVIKELSFLKEKGAIIGAGTVTSVEQCRKAVESGAEFIVSPH LDEEISQFCKEKGVFYMPGVMTPTELVKAMKLGHTILKLFPGEVV GPQFVKAMKGPFPNVKFVPTGGVNLDNVCEWFKAGVLAVGVGSAL VKGTPDEVREKAKAFVEKIRGCTE	C3 scaffold for O43-2B
O43-2A	MRGHHHHHHGSSALAYVMLGLLLSLLNRLSLAAEAYKKAIELDPN DALAWLLLGSVLEKLKRLDEAAEAYKKAIELKPNDASAWKELGKV LEKLGRLDEAAKAYAEAIKLDPSDAEAAKELGKVLEKLGQLELAE RAYQLAIELDPND	O43-2 cage C4/ *I*432-1 chain A
O43-2B	MRGHHHHHHGSSKMEELFKKHKIVAVLRANSVEEAKEKALAVFRG GVHLIEITFTVPDADTVIKELSFLKEKGAIIGAGTVTSVEQCRKA VESGAEFIVSPHLDEEISQFCKEKGVFYMPGVMTPTELVKAMKLG HTILKLFPGEVVGPQFVKAMKGPFPNVKFVPTGGVNDQNVCEWFK AGVLAVGVGSALVKGTPEQVEMLAVLFVAKIAGCTE	O43-2 cage C3
O43-2-D3-6B	MRGHHHHHHGSSKMEELFKKHKIVAVLRANSVEEAKEKALAVFRG GVHLIEITFTVPDADTVIKELSFLKEKGAIIGAGTVTSLEQCQKA VESGAEFIVSPHLDPEISKFCKINGVFYMPGVMTPTELVKAMKLG HTILKLFPGEVVGPQFVKAMKGPFPNVKFVPTGGVNDQNVCEWFK AGVLAVGVGSALVKGTPEQVEMLAVLFVAKIAGCTE	*I*432-1 chain B
O43-2A-CH	MALAYVMLGLLLSLLNRLSLAAEAYKKAIELDPNDALAWLLLGSV LEKLKRLDEAAEAYKKAIELKPNDASAWKELGKVLEKLGRLDEAA KAYAEAIKLDPSDAEAAKELGKVLEKLGQLELAERAYQLAIELDP NDLEHHHHHH	*I*432-1-CC chain A
O43-2-D3-6B-CH	MKMEELFKKHKIVAVLRANSVEEAKEKALAVFRGGVHLIEITFTV PDADTVIKELSFLKEKGAIIGAGTVTSLEQCQKAVESGAEFIVSP HLDPEISKFCKINGVFYMPGVMTPTELVKAMKLGHTILKLFPGEV VGPQFVKAMKGPFPNVKFVPTGGVNDQNVCEWFKAGVLAVGVGSA LVKGTPEQVEMLAVLFVAKIAGCTELEHHHHHH	*I*432-1-CC chain B
O43-2-D3-6B-7	MRGHHHHHHGSSKMEELFKKHKIVAVLRANSVEEAKEKALAVFRG GVHLIEITFTVPDADTVIKELSFLKEKGAIIGAGTVTSLEQCKKA VESGAEFIVSPHLDPEISKFCKIEGVFYMPGVMTPTELVKAMKLG HTILKLFPGEVVGPQFVKAMKGPFPNVKFVPTGGVNDQNVCEWFK AGVLAVGVGSALVKGTPEQVEMLAVLFVAKIAGCTE	*I*432-1-7 chain B
O43-2-D3-6B-8	MRGHHHHHHGSSKMEELFKKHKIVAVLRANSVEEAKEKALAVFRG GVHLIEITFTVPDADTVIKELSFLKEKGAIIGAGTVTSLEACRKA VESGAEFIVSPHLDPEISKFCKIAGVFYMPGVMTPTELVKAMKLG HTILKLFPGEVVGPQFVKAMKGPFPNVKFVPTGGVNDQNVCEWFK AGVLAVGVGSALVKGTPEQVEMLAVLFVAKIAGCTE	*I*432-1-13 chain B
O43-2-D3-6B-X3	MRGHHHHHHGSSKMEELFKKHKIVAVLRANSVEEAKEKALAVFRG GVHLIEITFTVPDADTVIKELSFLKEKGAIIGAGTVTDAKECARA VRSGAEFIVSPHLDPEISKFCKAQGVFYMPGVMTPTELVKAMKLG HTILKLFPGEVVGPQFVKAMKGPFPNVKFVPTGGVNDQNVCEWFK	*I*432-1-X3 chain B

	AGVLAVGVGSALVKGTPEQVEMLAVLFVAKIAGCTE	
O43-2-D3-6B-X6	MRGHHHHHHGSSKMEELFKKHKIVAVLRANSVEEAKEKALAVFRG GVHLIEITFTVPDADTVIKELSFLKEKGAIIGAGTVTDKRQCKKA VESGAEFIVSPHLDPEISEFCKMEGVFYMPGVMTPTELVKAMKLG HTILKLFPGEVVGPQFVKAMKGPFPNVKFVPTGGVNDQNVCEWFK AGVLAVGVGSALVKGTPEQVEMLAVLFVAKIAGCTE	*I*432-1-X6 chain B
O43-2-D3-6B-X9	MRGHHHHHHGSSKMEELFKKHKIVAVLRANSVEEAKEKALAVFRG GVHLIEITFTVPDADTVIKELSFLKEKGAIIGAGTVTDKRQCKKA VESGAEFIVSPHLDPEISEFCKMMGVFYMPGVMTPTELVKAMKLG HTILKLFPGEVVGPQFVKAMKGPFPNVKFVPTGGVNDQNVCEWFK AGVLAVGVGSALVKGTPEQVEMLAVLFVAKIAGCTE	*I*432-1-X9 chain B

Supplementary Table 4. Rosetta interface metrics for interfaces of crystals (orange), cages (blue) and cyclic oligomers (green). The crystal interfaces were evaluated from experimental X-ray crystallography models before and after Rosetta relax protocol for energy minimization. The cage and cyclic oligomer interfaces were evaluated from Rosetta design models. The values in gray with unrealistic deviations after Rosetta relax, which were potentially artifacts by low-resolution crystal models, were not used in the following analysis. SC: shape complementarity. SASA: solvent-accessible surface area. CMS: contact molecular surface.

Interfaces	SC	SASA/ Å^2	CMS/ Å^2	ddG/ kcal/mol
$F4_132$-1	0.556	839.013	127.595	-2.437
$F4_132$-1 relaxed	0.361	665.964	66.527	-9.280
$F4_132$-2	0.580	1197.138	230.042	2.751
$F4_132$-2 relaxed	0.135	778.201	40.452	-3.764
I432-1	0.516	634.632	92.920	-6.754
I432-1 relaxed	0.593	619.430	88.425	-6.170
T33-15	0.713	1355.685	216.105	-54.51
T32-15	0.536	1538.608	218.214	-50.677
O43-2	0.655	1308.395	218.662	-69.685
T33-ECY54	0.453	1162.437	235.631	-50.809
T33-ECY55	0.676	1062.419	175.44	-55.302
T33-ECY59	0.671	1086.636	179.141	-59.631
T33-ECY66	0.645	927.653	122.051	-44.382
T33-ECY67	0.594	1532.665	271.135	-77.667
O43-UWN38	0.679	987.774	145.462	-41.749
O43-UWN453	0.661	1185.968	216.101	-55.634
O43-ZL1	0.717	1193.553	243.974	-57.413
O43-ZL7	0.692	1286.934	274.764	-71.355
O32-ZL4	0.606	1358.55	239.474	-62.689
O43-2B	0.712	1304.045	367.563	-47.54
T32-15B	0.542	2383.041	863.529	-86.095
T33-ECY54B	0.721	1797.885	751.444	-58.498
T33-ECY59B	0.731	1969.575	797.62	-93.378
T33-ECY66B	0.739	2207.319	1070.902	-107.88

Supplementary Table 5. All-atom RMSDs between design model and X-ray crystallography model for each symmetry units of the designed crystals

Design	Cyclic-A	Cyclic-B	Cage	Dihedral
$F4_132$-1	0.42 Å	0.93Å	0.73 Å	1.19 Å
$F4_132$-2	1.73 Å	1.98 Å	1.86 Å	3.00 Å
*I*432-1	1.10 Å	1.00 Å	1.21 Å	1.34Å

Supplementary Table 6. Crystal structure data collection and refinement statistics

	**$F4_132$-1-0 (8CUU)**	**$F4_132$-1 (8CUV)**	**$F4_132$-1-3 (8CUW)**	**R3-1 (8CUX)**
**Data Collection**				
Space group	F 41 3 2	F 41 3 2	F 41 3 2	R 3 :H
Cell parameters				
a,b,c (Å)	289.61, 289.61, 289.61	293.55, 293.55, 293.55	285.99, 285.99, 285.99	50.22, 50.22, 116.00
α, β, γ (°)	90, 90, 90	90, 90, 90	90, 90, 90	90, 90, 120
Resolution (Å)	87.32 - 2.91 (3.01 - 2.91)	49.62 - 2.8 (2.9 - 2.8)	101.10 - 3.55 (3.67 - 3.55)	34.8 - 1.85 (1.91 - 1.85)
R_{merge}	0.241 (6.326)	0.122 (3.125)	0.258 (3.625)	0.114 (1.222)
R_{pim}	0.027 (0.700)	0.032 (0.521)	0.042 (0.572)	0.057 (0.618)
$I/\sigma(I)$	27.00 (0.93)	22.00 (0.98)	15.93 (1.06)	7.73 (1.31)
$CC_{1/2}$	1.000 (0.403)	0.999 (0.645)	0.999 (0.643)	0.995 (0.663)
Completeness (%)	99.94 (99.52)	99.17 (94.52)	99.95 (99.68)	99.94 (100.00)
Redundancy	79.4 (82.9)	39.7 (40.7)	39.2 (41.5)	4.9 (4.9)
**Refinement**				
Resolution (Å)	87.32 - 2.91 (3.01 - 2.91)	49.62 - 2.8 (2.9 - 2.8)	101.10 - 3.55 (3.67 - 3.55)	34.8 - 1.85 (1.91 - 1.85)
No. reflections	23382 (2286)	26999 (2537)	12632 (1235)	9321 (935)
R_{work} / R_{free} (%)	19.28 (31.57)/ 20.40 (36.33)	19.57 (28.63)/ 22.35 (34.31)	22.40 (33.47)/ 24.80 (39.34)	21.44 (27.40) /26.21 (31.97)
No. atoms				
Protein	2252	2207	2232	932
Ramachandran Favored/allowed/ Outlier (%)	95.21/4.45/0.34	97.55/2.45 /0.00	92.76/6.90 /0.34	96.58/3.42 /0.00
R.m.s. deviations				
Bond lengths (Å)	0.008	0.002	0.004	0.007
Bond angles (°)	1.08	0.50	0.66	0.84
$B_{factors}$ (Å^2)				
Protein	107.63	39.12	156.87	26.07

	$F4_132$-2 (8CWS)	$F4_132$-2-12
**Data Collection**		
Space group	F 41 3 2	F 41 3 2
Cell parameters		
a,b,c (Å)	405.41, 405.41, 405.41	403.65, 403.65, 403.65
α, β, γ (°)	90, 90, 90	90, 90, 90
Resolution (Å)	47.78 - 4.4 (4.55 - 4.4)	121.70 - 7.37 (7.63 - 7.37)
R_{merge}	0.381	0.1701 (1.716)
R_{pim}	0.381	0.040 (0.366)
$I/\sigma(I)$	1.00	18.73 (2.60)
$CC_{1/2}$	0.995	0.796 (0.847)
Completeness (%)	99.46 (100.00)	99.93 (100.00)
Redundancy	2.0 (2.0)	20.9 (22.8)
**Refinement**		
Resolution (Å)	47.78 - 4.4 (4.55 - 4.4)	121.70 - 7.37 (7.63 - 7.37)
No. reflections	18577 (1819)	4158 (403)
R_{work} / R_{free} (%)	33.59 (41.67)/ 35.14 (40.63)	23.29 (28.79)/ 27.88 (28.15)
No. atoms		
Protein	3930	3906
Ramachandran Favored/allowed/ Outlier (%)	99.20/0.60/0.20	98.40/1.60 /0.00
R.m.s. deviations		
Bond lengths (Å)	0.002	0.003
Bond angles (˚)	0.37	0.55
$B_{factors}$ (Å^2)		
Protein	170.49	484.38

	**I432-1(NaCl)(8CUS)**	**I432-1(Imd) (8CUT)**	**I432-1-X3 (8CWZ)**	**I432-1-CC (8FAR)**
**Data Collection**				
Space group	I 4 3 2	I 4 3 2	I 4 3 2	I 4 3 2
Cell parameters				
a,b,c (Å)	235.72, 235.72, 235.72	236.47, 236.47, 236.47	239.29, 239.29, 239.29	234.24, 234.24, 234.24
α, β, γ (°)	90, 90, 90	90, 90, 90	90, 90, 90	90, 90, 90
Resolution (Å)	48.12 - 3.98 (4.12 - 3.98)	46.38 - 4.00 (4.14 - 4.00)	169.2 - 5.53 (5.72 - 5.53)	82.82 - 3.66 (3.81 - 3.66)
R_{merge}	0.193 (3.678)	0.054 (0.165)	0.104 (1.169)	0.508 (0.651)
R_{pim}	0.031 (0.577)	0.05457 (0.1658)	0.023 (0.547)	0.188 (0.249)
$I/\sigma(I)$	24.51 (1.95)	22.06 (2.95)	23.35 (3.96)	22.98 (5.10)
$CC_{1/2}$	1.000 (0.680)	0.991 (0.917)	0.997 (0.861)	0.959 (0.891)
Completeness (%)	99.83 (100.00)	99.41 (96.46)	99.2 (97.3)	99.69 (97.82)
Redundancy	39.5 (41.1)	1.8 (1.3)	20.9 (15.4)	6.6 (6.8)
**Refinement**				
Resolution (Å)	48.12 - 3.98 (4.12 - 3.98)	46.38 - 4.00 (4.14 - 4.00)	169.2 - 5.53 (5.72 - 5.53)	82.82 - 3.66 (3.81 - 3.66)
No. reflections	9910 (969)	9814 (926)	3415 (243)	12399 (1330)
R_{work} / R_{free} (%)	17.71 (21.48)/ 21.66 (29.71)	18.75 (26.23)/ 22.49 (30.94)	24.11 (36.46)/ 28.72 (29.03)	19.60 (35.15)/ 23.47 (35.19)
No. atoms				
Protein	2552	2560	2562	2578
Ramachandran Favored/allowed/ Outlier (%)	93.07/6.02/0.90	96.70/3.30/0.00	87.43/9.88 /2.69	97.31/ 2.60/ 0.00
R.m.s. deviations				
Bond lengths (Å)	0.003	0.002	0.008	0.002
Bond angles (˚)	0.65	0.40	1.21	0.43
$B_{factors}$ (Å^2)				
Protein	61.06	71.57	286.140	201.40

Supplementary Table 7. CryoEM Data Collection Statistics

	**T32-15**	**O32-ZL4**
Microscope	Titan Krios	Titan Krios
Voltage (kV)	300	300
Detector	Gatan K3 Summit	Gatan K3 Summit
Recording mode	Super Resolution	Counting
Magnification	22,500 X	105,000 X
Movie micrograph pixel size (Å)	0.5144	0.8400
Dose rate ($e^{-}/Å^{2}/s$)	20	12.5
No. of frames per movie micrograph	50	75
Frame exposure time (ms)	50	40
Movie micrograph exposure time (s)	2.5	4.0
Total dose ($e^{-}/Å^{2}$)	50	60
Under focus range (μm)	0.8 - 2.2	0.8 - 1.8
Number of movie micrographs	4202	8821

Uncropped scans of gels in the SI

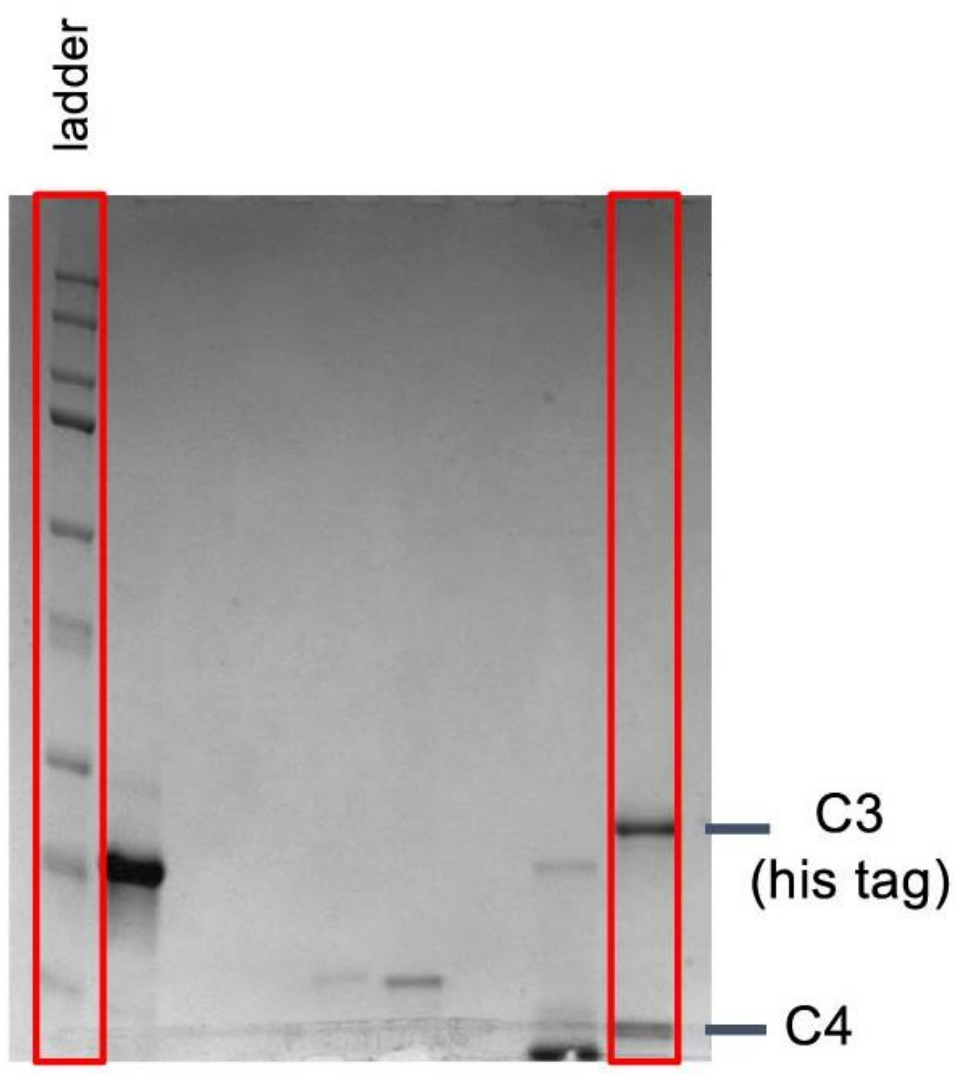

Supplementary Fig 10e

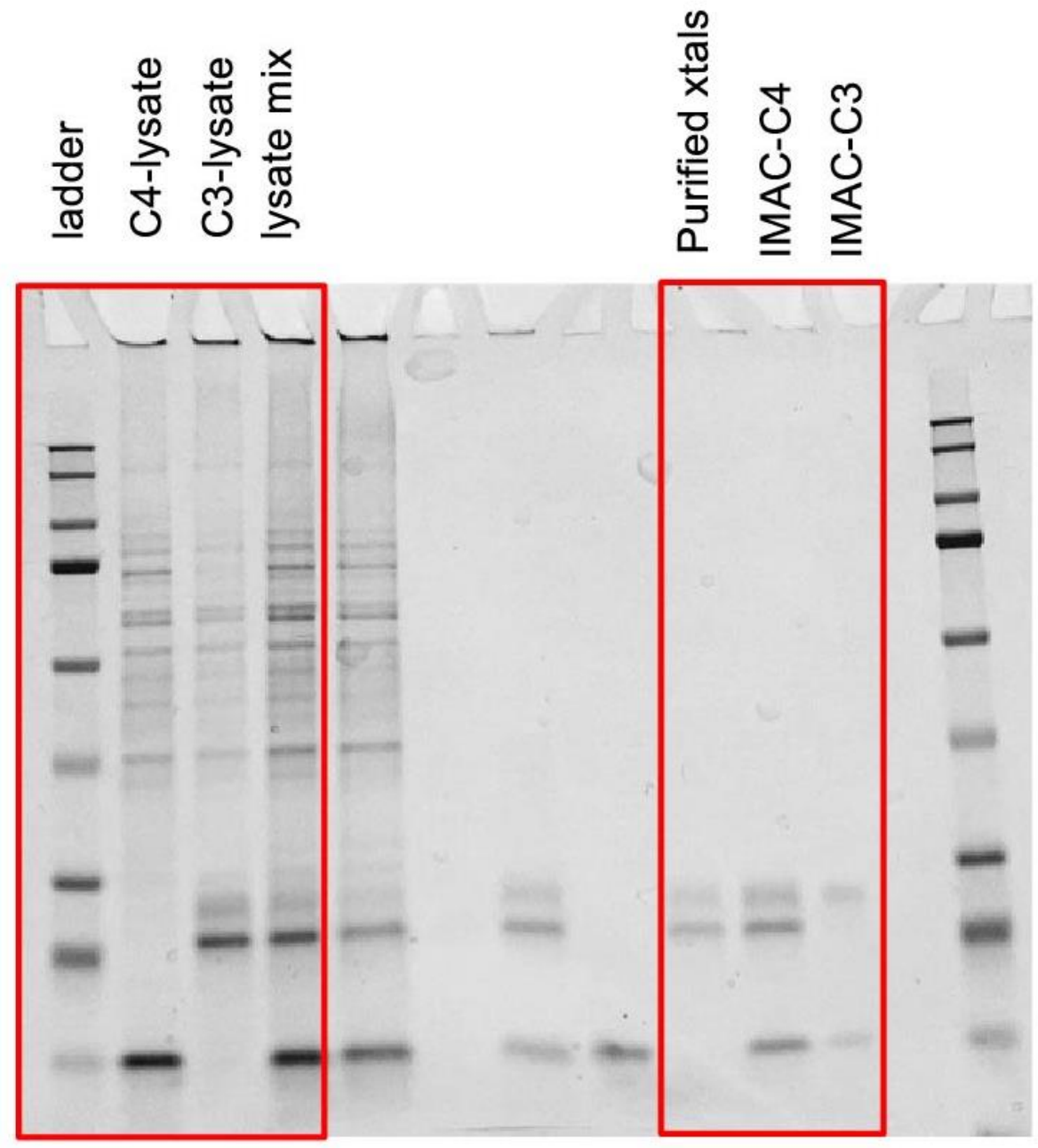

Supplementary Fig 15c
